# Design of a thermally controlled sequence of triazolinedione-based click and transclick reactions†
†Electronic supplementary information (ESI) available: Additional figures, experimental details, synthesis and analysis of all the model compounds and polymers, computational methods and relevant theoretical data. See DOI: 10.1039/c7sc00119c



**DOI:** 10.1039/c7sc00119c

**Published:** 2017-02-16

**Authors:** Hannes A. Houck, Kevin De Bruycker, Stijn Billiet, Bastiaan Dhanis, Hannelore Goossens, Saron Catak, Veronique Van Speybroeck, Johan M. Winne, Filip E. Du Prez

**Affiliations:** a Department of Organic and Macromolecular Chemistry , Polymer Chemistry Research Group and Laboratory for Organic Synthesis , Ghent University , Krijgslaan 281 S4-bis , 9000 Ghent , Belgium . Email: johan.winne@ugent.be ; Email: filip.duprez@ugent.be; b Preparative Macromolecular Chemistry , Institut für Technische Chemie und Polymerchemie , Karlsruhe Institute of Technology (KIT) , Engesserstraße 18 , 76131 Karlsruhe , Germany; c Center for Molecular Modeling , Ghent University , Technologiepark 903 , 9052 Zwijnaarde , Belgium; d Department of Chemistry , Bogazici University , 34342 Bebek , Turkey

## Abstract

The reaction of triazolinediones (TADs) and indoles is of particular interest for polymer chemistry applications, as it is a very fast and irreversible additive-free process at room temperature, but can be turned into a dynamic covalent bond forming process at elevated temperatures, giving a reliable bond exchange or ‘transclick’ reaction. In this paper, we report an in-depth study aimed at controlling the TAD–indole reversible click reactions through rational design of modified indole reaction partners. This has resulted in the identification of a novel class of easily accessible indole derivatives that give dynamic TAD-adduct formation at significantly lower temperatures. We further demonstrate that these new substrates can be used to design a directed cascade of click reactions of a functionalized TAD moiety from an initial indole reaction partner to a second indole, and finally to an irreversible reaction partner. This controlled sequence of click and transclick reactions of a single TAD reagent between three different substrates has been demonstrated both on small molecule and macromolecular level, and the factors that control the reversibility profiles have been rationalized and guided by mechanistic considerations supported by theoretical calculations.

## Abstract 2

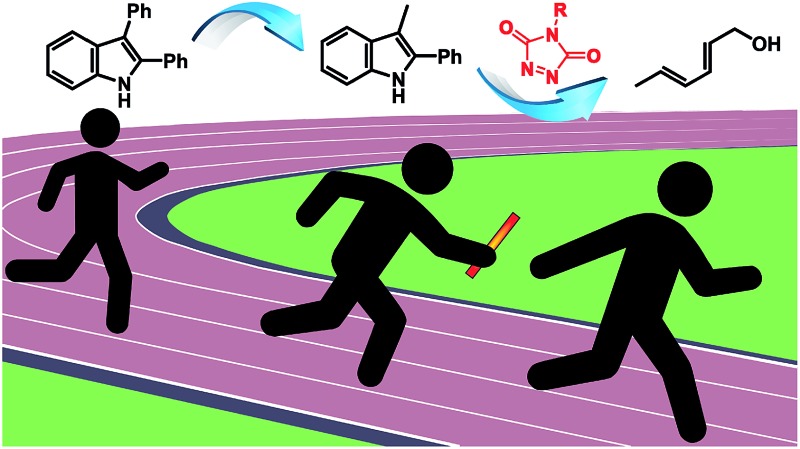
An unprecedented relay of triazolinedione-based transclick reactions between three different substrates has been demonstrated both on small molecule and macromolecular level.

## Introduction

The introduction of ‘click’ chemistry by Sharpless and coworkers,^1^ made a huge impact on the chemical community.^2^ The desirable characteristics that such reactions should possess (simple conditions, modular, wide in scope, high yielding, fast reaction rates, chemoselective, stable reaction products, simple isolation, a single reaction trajectory, thermodynamically spring loaded and benign or no byproducts), became widely accepted in the search for ‘new’ covalent bond-forming chemistries that can have very wide applications.^3^ As a result, the click chemistry concept has been rapidly picked up by multiple research disciplines, in particular in polymer chemistry and materials science.^4^ Eventually, this resulted in additional key requirements to achieve ‘true’ click behavior in a macromolecular context (scalability, equimolarity).^5^

Since the original introduction of the click chemistry concept, a whole range of different reactions have appeared that can meet all or most of the desired characteristics, with a central role for copper-catalyzed azide–alkyne cycloadditions (CuAAC)^6^ and metal-free alternatives thereof.^7,8^

An emerging theme in click-inspired reactions, especially in the field of polymer chemistry, is the use of reactions that can also be efficiently reversed or ‘unclicked’.^9–15^ This reversibility feature at first seems contradictory to some of the original Sharpless criteria such as the need for stable reaction products and the ‘spring loaded’ thermodynamic driving force. Nevertheless, there are many attractive applications in materials science that require a certain extent of reversible behavior for covalent bond forming reactions. Indeed, dynamic covalent chemistry is now a very active research topic in the design of functional materials with the ability to be healed, repaired, recycled or that can respond to external triggers.^16,17^

**Scheme 1:**
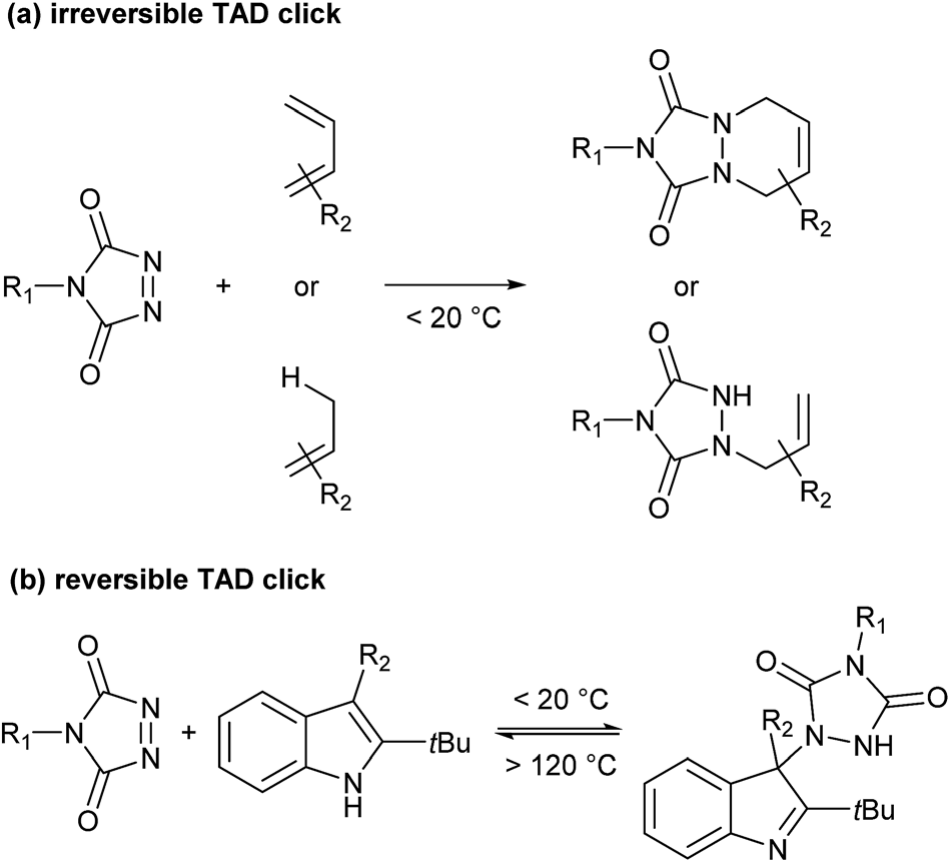
(a) Irreversible and (b) reversible TAD click reactions.

Recently, Du Prez and coworkers reported a simple polymer conjugation strategy based on the unique reactivity of the triazolinedione (TAD) moiety.^18–21^ TAD is well-known to undergo a range of very fast Diels–Alder- or Alder–ene-type bond-forming reactions with various olefinic reaction partners, at or even below room temperature, and under equimolar conditions (Scheme 1a). Moreover, TAD was found to be a reactant that can combine the original click-type characteristics with the possibility to form a stable but thermally reversible covalent bond. Because of the very low forward reaction barriers of TAD reactions – reactions are often almost instantaneous at or below room temperature – a backward reaction can indeed become feasible at higher temperatures, even for highly exergonic reactions.

**Scheme 2:**
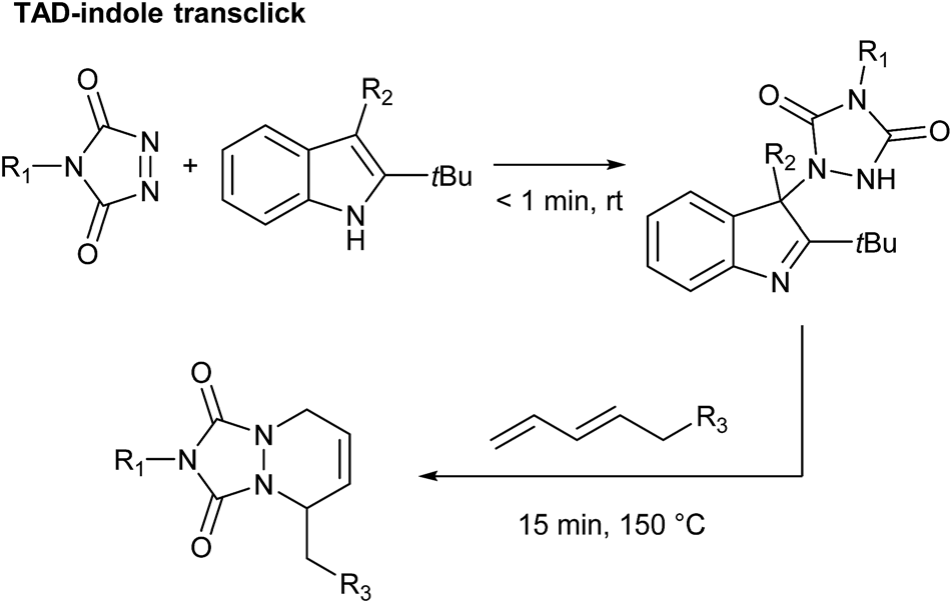
TAD–indole transclick reaction: the click-like transfer of a TAD reagent from one reaction partner to another.

By carefully choosing the olefinic bond forming partner, a reversible TAD click reaction can be obtained.^22–24^ Indoles for instance, were found to be excellent substrates for the development of reversible TAD-based click reactions (see Scheme 1b). These robust and easily prepared heterocyclic scaffolds smoothly react with TAD in a click-like manner at room temperature with reaction half-lives in the order of minutes or less. When the resulting adduct is heated above ∼100 °C, the reversible nature of the bond forming process establishes itself, providing reshaping and healing properties to TAD–indole-based materials. The dynamic high temperature reaction of the TAD–indole adduct formation was not only found to be a very clean and reliable transformation, it was also discovered that the liberated TAD moiety could be reacted (*in situ*) with another substrate, such as a conjugated diene, in a highly orthogonal way. Trying to generalize this remarkable dynamic behavior, *i.e.* the exchange of a ‘clickable’ TAD group between two different molecules – indole and conjugated diene – we coined the concept of a ‘transclick’ reaction (see Scheme 2). A transclick reaction has been defined as ‘any covalent linking process that can subsequently be triggered to form a new bond with an alternative or orthogonal reaction partner, and at the same time release one of the original binding partners, in which both bond forming steps meet the usual requirements for ‘click’ reactions'.^18^

In this paper, we now present a full account of our investigations into the TAD–indole click and transclick reaction platform, of which we have expanded the scope quite significantly. Guided by the objective to be able to control the temperature at which the TAD–indole conjugation becomes reversible, novel simple indole reaction partners were rationally designed and explored. It was found that further lowering the temperature at which the retro-reaction proceeds, is indeed possible by judiciously designing the indole scaffold. This ‘molecular engineering’ of the thermal behavior of the TAD–indole click reaction has been further rationalized and supported by Density Functional Theory (DFT) based calculations. Finally, we demonstrate the practicality of our approach by designing cascades of thermally controllable (trans)click reactions, covalently relaying a dye-functionalized TAD-compound between various substrates, including macromolecular structures.

## Results and discussion

### Synthesis and exploration of novel indole substrates for TAD transclick reactions

**Scheme 3:**
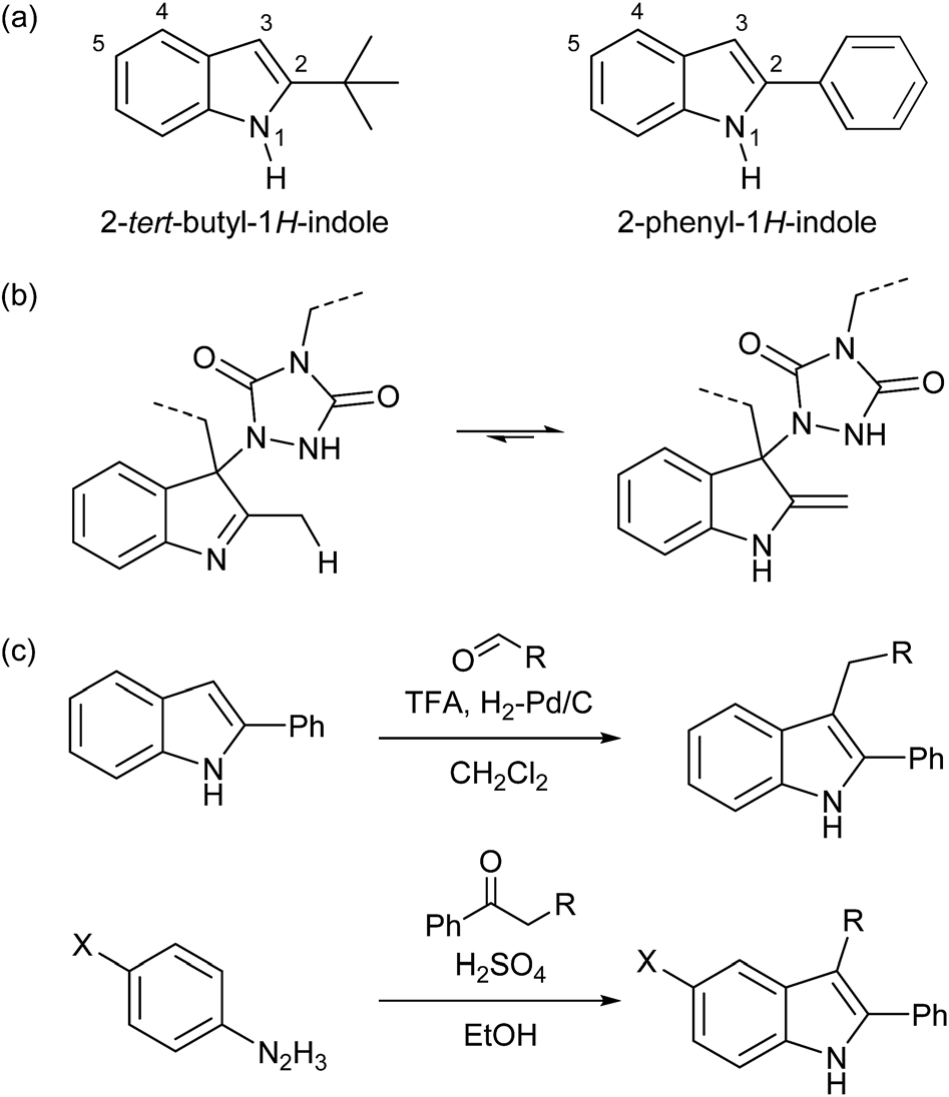
(a) Former (left) and new (right) heterocyclic scaffold for suitable TAD-reactive indoles, (b) undesirable imine–enamine tautomerization in indole substrates with a C2-substituent that has acidic protons, (c) straightforward synthesis procedure for functionalized 2-phenyl-1*H*-indoles used in this work.

For our original explorations of TAD–indole click reactions, substituted 2-*tert*-butylindoles (*cf.* Scheme 1b) were used as readily available model substrates.^18^ The 2-*tert*-butyl-1*H*-indole building block (Schemes 3a and S1†) from which these were derived is commercially available and can also be prepared on a relatively large lab scale (∼50 g). However, as we now identified 2-phenyl-1*H*-indole as an even more easily accessible (and cheaper) starting product, this compound was used as a versatile building block in this work. Interestingly, 2-phenyl-1*H*-indole is in fact a common additive for bulk PVC materials (stabilizer), including food packaging plastics, often used for more than 1% w/w.^25^ As for 2-*tert*-butyl-1*H*-indoles, their 2-phenyl-1*H*-indole analogues are unable to undergo imine–enamine tautomerization (Scheme 3b, also see Scheme S2†), which gives it the same reliable TAD-reactive properties, without risking undesired side reactions arising from the enamine tautomer. Furthermore, reversibility can only be ensured by the presence of a substituent on the indole C3-position, thereby excluding re-aromatization to occur. Such a functionalization can be achieved by exploiting the nucleophilicity of the C3-position, for example in a simple reductive alkylation with aldehydes, giving access to a range of suitable indole derivatives (Scheme 3c).

An additional advantage of the 2-phenyl-1*H*-indole as a new TAD-reactive scaffold introduced in this work, is its alternative synthesis from readily available bulk building blocks (aromatic hydrazines and ketones) *via* the well-known Fischer indole synthesis (Schemes 3c and S3†).^26,27^ In this way, a wide range of 2-phenyl-1*H*-indoles can be readily prepared *via* a one-step process, making it possible to introduce different substituents at once, both on the C2/C3- and C5-positions, by changing the phenylketone or phenylhydrazine reaction partner, respectively.

**Fig. 1:**
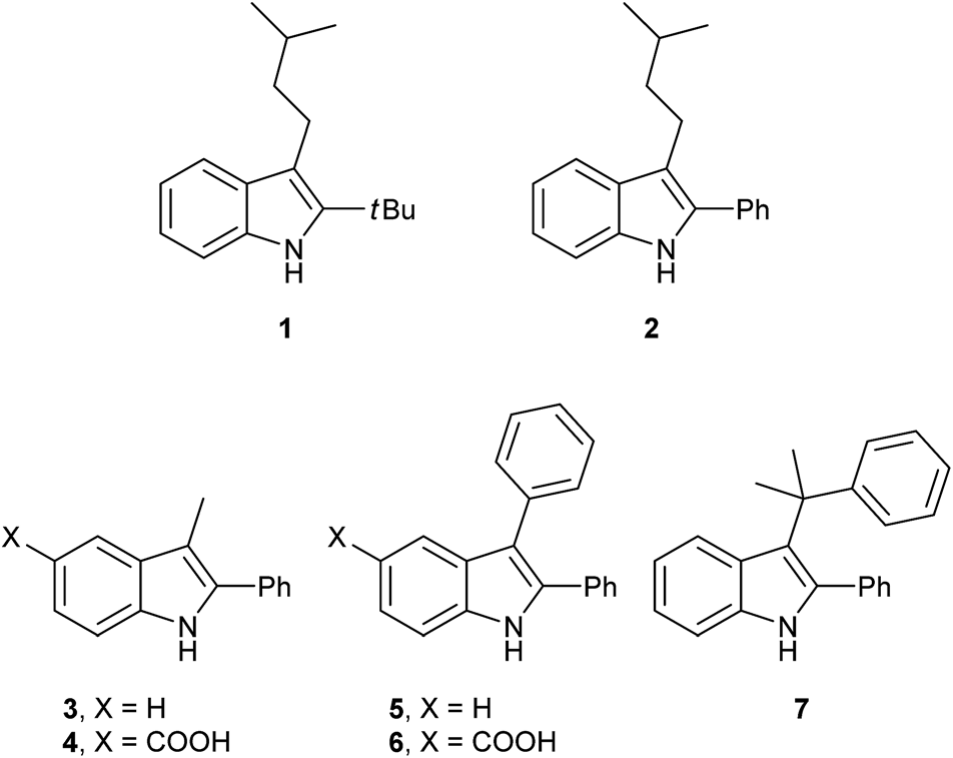
Investigated indole substrates in TAD click reactions.

Having synthesized a range of different indole compounds according to the above described methods (Fig. 1, also see Table S1†), their reactivity toward 4-*n*-butyl-1,2,4-triazoline-3,5-dione (BuTAD) was investigated. Prior to exploring the effect of the different substitution pattern on the reversibility of the TAD–indole reaction, the corresponding forward reaction was briefly investigated to verify the retention of its typical click characteristics.

First, the effect of replacing the 2-*tert*-butyl group in our previously applied indole **1** (Fig. 1) by a phenyl group was investigated in detail. Therefore, 2-phenyl-3-isopentyl-1*H*-indole (**2**) was reacted with an equimolar amount of BuTAD in DMSO-*d*_6_ and verified *via* ^1^H-NMR. The reaction went to completion in a few seconds, as judged by the disappearance of the red color, and resulted in a single reaction product (see Fig. S1† for typical rate constants). This TAD–indole reaction proceeded at a very similar rate compared to the reaction between 2-*t*Bu-3-isopentyl-1*H*-indole (**1**) and BuTAD, which goes to completion in less than a minute at room temperature. Similar results were obtained when the 3-isopentyl substituent in **2** is replaced with a methyl group and when an electron withdrawing group is present on the indole core, *cf.* 3-methyl-2-phenyl-1*H*-indole (**3**) and 5-carboxy-3-methyl-2-phenyl-1*H*-indole (**4**), respectively. Although electron withdrawing substituents might lead to a reduced nucleophilicity of the indole core, no significant difference in forward reaction rate and efficiency was observed. Thus, 2-phenyl-1*H*-indoles react in the orders of seconds with TAD, independent of the aliphatic chain length on the C3 or the presence of an electron withdrawing functional group at the C5-position.

**Fig. 2:**
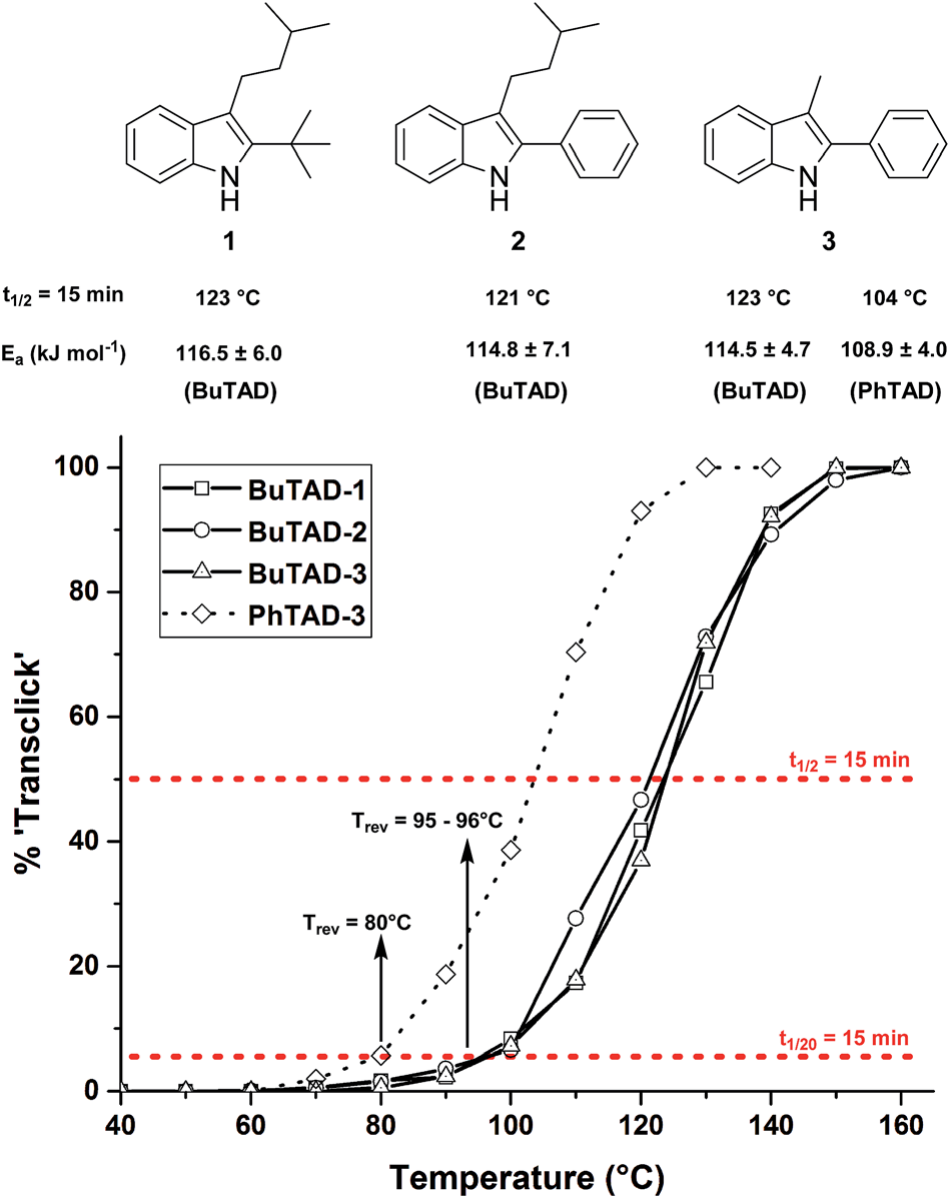
Investigated 2-*tert*-butyl- and 2-phenylindoles **1–3** and their respective reversibility curves ([TAD-adduct]_0_ = 0.04 M). The red lines represent the 5% and 50% level from which the reversibility temperature and half-life temperature are determined for each indole.

Guided by further mechanistic considerations (*vide infra*), we reasoned that increasing the steric bulk at the reactive C3-center of the indole might have a more pronounced effect on the reactivity with TAD reagents, both for the forward and, in particular, the backward reaction barriers. Thus, the reactions of BuTAD with the sterically more demanding 2,3-diphenylindoles **5–6** were investigated. When the plain 2,3-diphenyl-1*H*-indole (**5**) was reacted with BuTAD, the reaction did go to completion, but only after a significantly extended reaction time, *i.e.* up to 3 hours instead of just a few seconds (Fig. S2†). Again, introducing a carboxylic acid as functional group on the indole core did not affect the reactivity toward TADs, as qualitatively judged by the comparable efficiency (single reaction product) and forward reaction rate of indole **6**. Finally, by further increasing the steric bulk at the C3-position, as for indole **7**, the reaction with TAD no longer occurred at room temperature nor at elevated temperatures, as not even a trace of the expected adduct was formed. A range of other related indoles with varying steric bulk at C3 were also prepared and investigated, but none performed better than the 2,3-Ph_2_-substituted indoles (see ESI, Fig. S3†). Encouraged by the observed differences in reaction rate for the forward reaction, and the retention of click-like characteristics even for sterically hindered indoles (*i.e.* high yielding and single product under equimolar conditions), the newly synthesized TAD–indole adducts obtained from BuTAD and indoles **2–6** were subjected to kinetic reversibility tests to assess differences in backward reaction barriers. We had previously designed a straightforward protocol to kinetically examine the reversible adduct formation between indoles and TADs.^18^ By simply reacting equimolar amounts of BuTAD and an indole in DMSO-*d*_6_, and subsequently adding a slight excess of *E*,*E*-2,4-hexadien-1-ol (HDEO) to the resulting reaction mixture after completion, a kinetic and thermodynamic ‘trap’ for any released TAD reagents is assured. Indeed, this trapping diene reacts irreversibly with TADs with a reaction rate that is several orders of magnitude higher than that of the reaction between TAD and indoles (*k*(diene)/*k*(indole) > 10^3^, Fig. S1†). Thus, by heating aliquots of this mixture for 15 minutes at a fixed temperature, a simple thermal reversibility profile can be obtained from the integration of the proton NMR spectrum of the resulting reaction mixture (see Fig. S13† for details). These profiles show the total amount (%) of TAD that has been released from its indole adduct at a given temperature over a 15 minute period (Fig. 2). Because the forward reaction is a first order fragmentation, and the backward TAD–indole recombination reaction can be neglected, these profiles also readily show the temperature at which the reaction half-life is 15 minutes (intersection of the S-curve and the 50% release line). These data points thus allow for the calculation of the experimental *k*-values and activation energies. For the 2-*tert*-butylindoles used in our previous study such as **1**, the reaction has a half-life of 15 minutes at about 121 °C, and a calculated activation energy (by fitting ln *k* to 1/*T*) of 116.5 ± 6.0 kJ mol^–1^. As a useful qualitative indicator of the reversibility, we can also estimate the temperature at which 5% of TAD reagents have been released over a 15 minute period (*i.e. t*_1/20_ = 15 min), which gives a macroscopic ‘reversibility temperature’.^28^ This can be seen as the temperature where the reversibility of the TAD–indole reaction will become significant for most applications (leading to stress relaxation or other dynamic properties).

The two 2-phenyl-3-alkylindoles **2** and **3** were found to show reversibility profiles that are almost superimposable with those obtained for the 2-*tert*-butylindole (**1**) in their adduct formation with BuTAD, and show an onset temperature of reversibility (*t*_1/20_ = 15 min) of 95 (indole **2**) and 96 °C (indole **3**), which is in perfect agreement with the 95 °C observed for indole **1** (Fig. 2). Kinetic fitting of a first order reaction to the obtained data points gave statistically indistinguishable activation energies, *i.e.* 114.8 ± 7.1 and 114.5 ± 4.7 kJ mol^–1^ for indoles **2** and **3**, respectively. Thus, we can conclude that the small differences in steric bulk around C2 (phenyl *vs. tert*-butyl) does not lead to a significant effect on either the forward or the reverse reaction barriers. Nevertheless, this study identifies 2-phenylindoles as preferred substrates for TAD–indole (trans)click reactions because of their more straightforward synthetic design and availability. Remarkably, in this study we have also found that these indoles give a reversible adduct formation with PhTAD with a significant shift in the thermal reversibility profile of about 15–20 °C (reaction half-life of 15 min at 104 °C, with a lower experimental activation energy, see Fig. 2). This unexpected result is a first clear indication of the ‘tunability’ of the reversible TAD–indole click reaction.

**Fig. 3:**
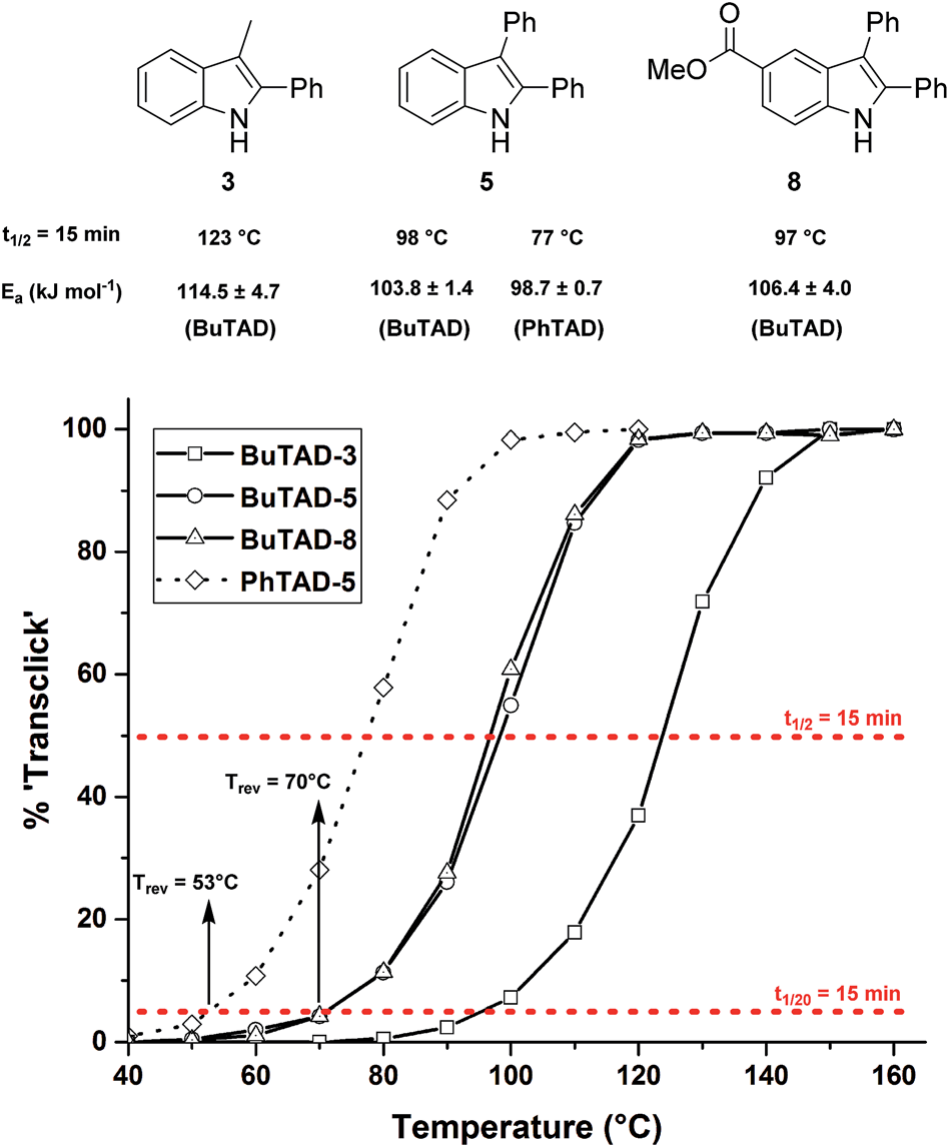
Kinetic comparison of the retro-reaction of indoles with more bulky C3-substituents ([TAD-adduct]_0_ = 0.04 M). The red lines represent the 5% and 50% level from which the reversibility temperature and half-life temperature are determined for each indole.

We next explored the indole substrates **5–7** with a significantly increased steric bulk at the C3-position. From theoretical rationalizations (*vide infra*), it is expected that this steric crowding could have an accelerating effect on the backward reaction. Indeed, with BuTAD, the 2,3-diphenyl-1*H*-indole (**5**) was found to show a remarkably different reversibility profile, shifted by 20–25 °C compared to the less hindered 3-methyl-2-phenyl-1*H*-indole (**3**) (Fig. 3). A reaction half-life of 15 minutes is now observed at 98 °C (compared to 123 °C for **3**), whereas the onset reversibility temperature dropped to 70 °C (compared to 96 °C for **3**). Kinetic fitting of a first order reaction gives an experimental activation energy of 103.8 ± 1.4 kJ mol^–1^ compared to 114.5 ± 4.7 kJ mol^–1^ for the release of **3**. Furthermore, the TAD indole-to-diene transclick reaction is completed in 15 minutes when heated at 120 °C. This very clear increase in reactivity of the TAD–indole adduct should be quite useful for transclick-type applications and shows that control of the reversibility temperature is feasible. As an additional test for the applicability of these reactions, the effect of a carboxylic ester substituent, which can serve as a synthetic handle to incorporate these indoles into various compounds and materials, was investigated. As can be seen in Fig. 3, the ester-functionalized indole **8** shows an almost superimposable thermal reversibility profile as the unsubstituted parent indole **5**. Finally, we again found a remarkable and very similar shift in the reversibility profile by switching from BuTAD to PhTAD. Thus, by simple structural changes in the indole and TAD reaction partners, their dynamic click reaction can be determined, almost at will, over a 50 °C range. An overview of the main structural types of indoles investigated herein and their reactivity parameters can be found in Table 1.

**Table 1:** Overview of substituted indole substrates investigated, their reaction time for quantitative BuTAD-addition at room temperature and the temperature range in which the transclick reaction can be affected (0.04 M, DMSO-*d*_6_)^*a*^

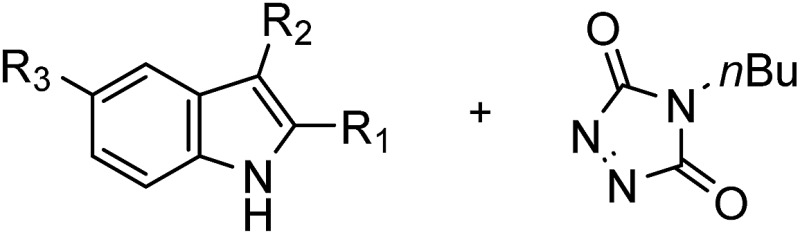					
	R_1_	R_2_	R_3_	Click reaction time	Transclick temperature
**1**	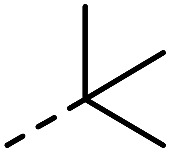	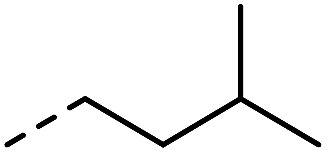	–H	1 min	95–150 °C
**2**	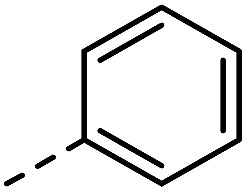	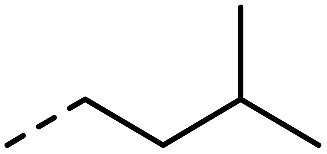	–H	10 s	95–150 °C
**3**	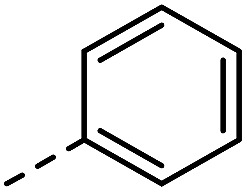	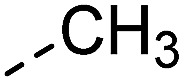	–H	10 s	95–150 °C
**4**			–COOH		n.d.
**5**	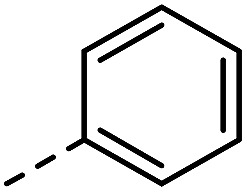	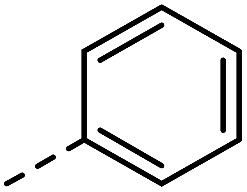	–H	3 h	70–120 °C
**6**			–COOH		n.d.
**8**			–COOMe		70–120 °C
**7**	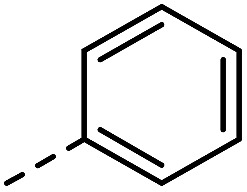	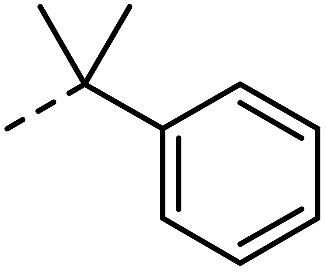	–H	n.r.	n.a.

^*a*^n.d.: not determined. n.r.: no reaction. n.a.: not applicable.

### Theoretical rationalization

**Fig. 4:**
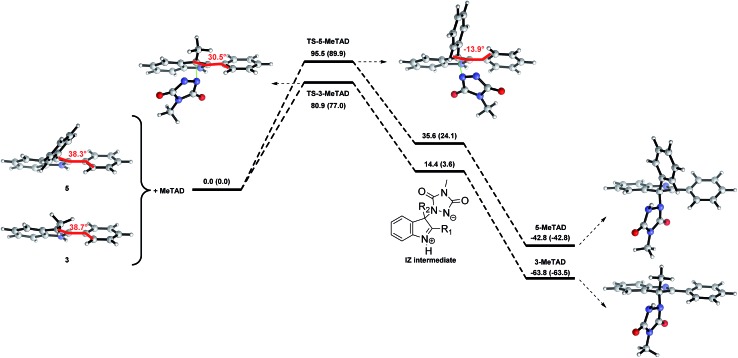
Gibbs free energy profiles (kJ mol^–1^) for the reactions of 3-methyl-2-phenyl-1*H*-indole (**3**) and 2,3-diphenyl-1*H*-indole (**5**) with MeTAD, calculated at 298 K and 1 atm. PCM (*ε* = 8.93 (CH_2_Cl_2_, forward reaction) or 46.7 (DMSO, reverse reaction, in parenthesis)), M06-2X/6-31++G(d,p), IZ = iminium–urazolide zwitterionic intermediate, dihedral angles are shown in red.

In our previous studies, we proposed a reaction mechanism for the TAD–indole reaction *via* an open iminium–urazolide zwitterionic intermediate based on theoretical calculations.^18^ This earlier mechanistic proposal is in agreement with the experimental findings and it offered useful insights and guidance for the design of new experiments. We have now expanded this theoretical work to include the various types of modified indole substrates investigated here (*vide supra*), in order to rationalize the remarkable effects of the indole substitution patterns on the reaction kinetics. The calculated Gibbs free activation energies for the forward and the backward reactions of the indoles **1–6** are shown in Table 2. The experimentally observed lower forward reaction rates for 2,3-diphenylindoles **5** and **6** *versus* 2-*tert*-butyl-3-alkylindoles **1** and 2-phenyl-3-alkylindoles **2**, **3** and **4** are nicely reproduced by the calculations (Fig. 4). However, while experimentally no qualitative difference in forward reaction rates was found by adding a carboxyl group on the C5-position, calculations predict a somewhat slower reaction for 5-COOH-indoles **4** and **6** *versus* indoles **3** and **5**, respectively.

**Table 2:** Gibbs free activation energies (kJ mol^–1^) for the forward and the backward reactions of substituted indoles **1–6** with MeTAD and their comparison with the experimentally obtained activation energies (kJ mol^–1^) for the backward reaction^*a*^

Indole	Δ*G*‡forward	Δ*G*‡backward	*E*_a_
**1**	77.5	135.2	116.5 ± 6.0
**2**	78.9	140.6	114.8 ± 7.1
**3**	80.9	140.4	114.5 ± 4.7
**4**	89.5	141.8	n.d.
**5**	95.5	132.8	103.8 ± 1.4
**6**	102.2	132.9	106.4 ± 4.0

^*a*^PCM (*ε* = 8.93 (CH_2_Cl_2_, forward reaction) or 46.7 (DMSO, backward reaction)) M06-2X/6-31++G(d,p). n.d.: not determined.

Because the backward reactions of the reversible TAD–indole conjugation have been kinetically examined, with the determination of experimental activation energies, a more useful comparison can be made by comparing trends between observed activation barriers and predicted backward free energy reaction barriers. The two groups of indoles, having either a simple primary alkyl chain or a sterically demanding phenyl group at the nucleophilic C3-position, which are separated experimentally by nearly 10 kJ mol^–1^ in activation barriers (see Fig. 4) also clearly show a difference in calculated backward free energy barriers (Table 2). Thus, the experimentally observed trends in backward reaction rates for the TAD-adducts of indoles **1–6** are qualitatively reproduced by the calculations. From our computational results, the lower backward reaction barrier for indoles **5** and **6** (*cf.* **8** with COOMe instead of COOH on the C5-position) can be attributed to a significant reduction in the exergonicity of the forward reaction: the reaction of indole **5** with TAD is calculated to be 21 kJ mol^–1^ less exergonic compared to the reaction of indole **3**, while the relative destabilization of the transition state is only 13 kJ mol^–1^ (Fig. 4). The resulting net effect of lowering the backward barrier can also be related to the simple principle put forward in the Hammond postulate, as the steric hindrance should be less important for the ‘earlier’ transition state of a more exothermic process, as compared to the steric hindrance in the final product. These findings confirm a straightforward design strategy to decrease the backward reaction barriers, thus achieving the desired control of the temperature at which the TAD–indole click reaction becomes dynamic.

Interestingly, this theoretical work also points towards a possible additional electronic effect of the C3-phenyl substituent. Indeed, the optimized geometries for the transition states of 3-methyl-2-phenylindoles (**3**) and 2,3-diphenylindoles (**5**) show a remarkable difference in conformation (Fig. 4). In the ground states of indoles **3** and **5**, the C3-phenyl substituent is not coplanar with the indole ring. However, in the calculated transition states of the addition to the TAD reagent, these two aromatic rings become significantly more coplanar for indole **5**, implying an increased electronic conjugation between the two rings. Electron donating or withdrawing substituents on the C3-phenyl ring may thus be expected to further alter the reaction kinetics, even if there is little electronic conjugation in the ground states of the indoles and the TAD–indole adducts. The 2-phenyl moiety becomes almost coplanar with the indole in the transition state (dihedral angle of –14° for **TS-5-MeTAD**), while this is much less the case in the TS of indole **3** (+31°), or even in the isolated indole **5** (+38°). Moreover, in the transition states for the 2,3-diphenylindole **5** we have found that there is a significant preference for the orientation of the TAD ring towards the 2-phenyl ring, which is almost coplanar with the indole ring. In contrast, no such rotational preference is observed in the transition states between TAD and 3-methyl-2-phenylindole **3**. This again points towards a possible electronic effect of the 2-phenyl substituent.

### Investigation of indole-to-indole TAD transclick reactions

After the successful identification of the novel class of reactive 2-phenylindoles that have a ‘tunable’ forward and backward reactivity with TADs depending on their C3-substituent, we decided to study the possible exchange reaction of a TAD compound between two indole substrates. We had previously used reversible TAD–indole click reactions as dynamic exchange reactions for the production of covalent adaptable networks.^18^ The different reactivity of various 2-phenylindole derivatives now opens the possibility of also having a directed or ‘programmed’ transclick reaction from one indole substrate to another. Such an unprecedented control of the TAD–indole click reactions can have interesting applications for the design of novel dynamic macromolecular systems. We first investigated this intriguing possibility on low MW compounds, using a similar approach as before.

**Fig. 5:**
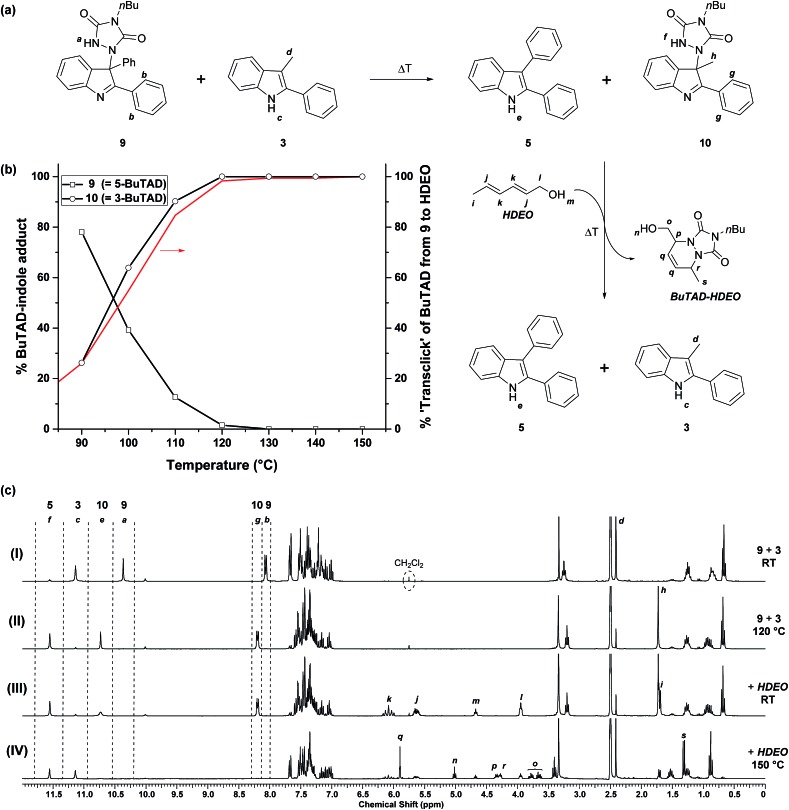
(a) Selective exchange of the TAD–indole adducts when a reaction mixture of ene-adduct **9** and indole **3** is heated, (b) graphical representation of the indole-to-indole transclick reaction as a function of temperature where the decrease of adduct **9** in the reaction mixture is compensated with the increase of adduct **10**. The red line represents the maximal fraction of transfer, as deducted from the kinetic reversibility study of indole **5** and (c) ^1^H-NMR spectra after heating show the exchange of the indole N–H and urazole N–H signals. 15 minutes of heating at 150 °C in the presence of HDEO in the end releases indole **3**.

As a first experiment, a similar protocol for the reversibility experiments as described above was investigated, by using an indole instead of a diene (HDEO) as ‘trapping agent’ for released TAD compounds. Thus, one equivalent of the sterically less hindered ‘receptor’ indole **3** was added to aliquots of a DMSO-*d*_6_ stock solution of the adduct **9**, obtained from a click reaction between BuTAD and the sterically more hindered ‘donor’ indole **5** (Fig. 5a). At room temperature, this two-component mixture is stable and shows that all BuTAD molecules are bonded in the adduct **9** while 3-methyl-2-phenyl-1*H*-indole (**3**) is present as unreacted indole (see Fig. 5c(I)). Heating the sample for a 15 minute period should lead to the release of BuTAD and the donor indole **5** from their adduct **9**. BuTAD can subsequently react with either of the two indoles **3** or **5** present in the reaction mixture, leading to an exchange of the TAD group between different indoles. As the acceptor indole **3** is known to react faster with TADs, and is also more sluggish in the reversible reaction, a net transfer of the TAD moiety from the donor to the acceptor indole can be expected to some extent at lower temperatures (kinetic control), whereas a thermodynamic mixture (also favoring the acceptor indole) should be obtained at higher temperatures.

The obtained reversibility profiles are depicted in Fig. 5b. The profile is almost superimposable with the one observed when HDEO is used as a kinetic trap, suggesting that the transclick reaction of the TAD group between indole **5** and **3** is kinetically controlled. Any released TAD reagent (either from the donor adduct or the acceptor adduct) thus reacts quite selectively with the less hindered indole moiety. This is also supported by the observation that BuTAD reacts exclusively with indole **3** when mixed with an equimolar mixture of **3** and **5** at room temperature (see ESI, Fig. S4†). The efficiency of this clean and high yielding equimolar transclick reaction can be appreciated from the NMR spectra (Fig. 5c) obtained in these reversibility experiments. It can be observed that the indole-to-indole transclick reaction goes to complete conversion (>99%) if the reaction mixture is heated at 120 °C for 15 minutes (spectrum (II), also see Fig. S14†). At higher temperatures, the yield does not decrease, indicative of a strong thermodynamic preference for the newly formed adduct **10**.

In a second low MW experiment on the indole-to-indole transclick reactions, the reaction mixture obtained above, containing the liberated donor indole **5** and the newly formed adduct **10**, was treated with 1 equivalent of HDEO diene as an irreversible TAD scavenger. The resulting mixture in DMSO-*d*_6_ at room temperature, shows the expected unreacted starting materials **5**, **10** and HDEO (Fig. 5c(III)). When this mixture is then heated for 15 minutes at 150 °C, a quantitative irreversible transclick reaction is induced. Analysis of the obtained ^1^H-NMR spectrum (IV) shows complete liberation of the previous acceptor indole **3** and formation of the expected BuTAD–HDEO adduct. It should be noted that the reaction temperature for this final transclick reaction should not be increased above 150 °C, since above this temperature TAD-dimerization – with loss of nitrogen gas – can become an important side reaction and hence can severely reduce the efficiency of the transclick reaction (see Scheme S4†).

In conclusion, by using varyingly substituted 2-phenylindoles, a thermally controlled cascade of transclick reactions has been developed that allows for the selective transfer of a TAD group between three consecutive reaction partners.

### Transfer of functionalized TAD compounds *via* transclick reactions

After establishing the efficiency of the newly introduced TAD–indole based reactions on low molecular weight compounds, we aimed to demonstrate the potential of these versatile and robust transclick reactions in a more realistic context, using functionalized TAD compounds.

**Scheme 4:**
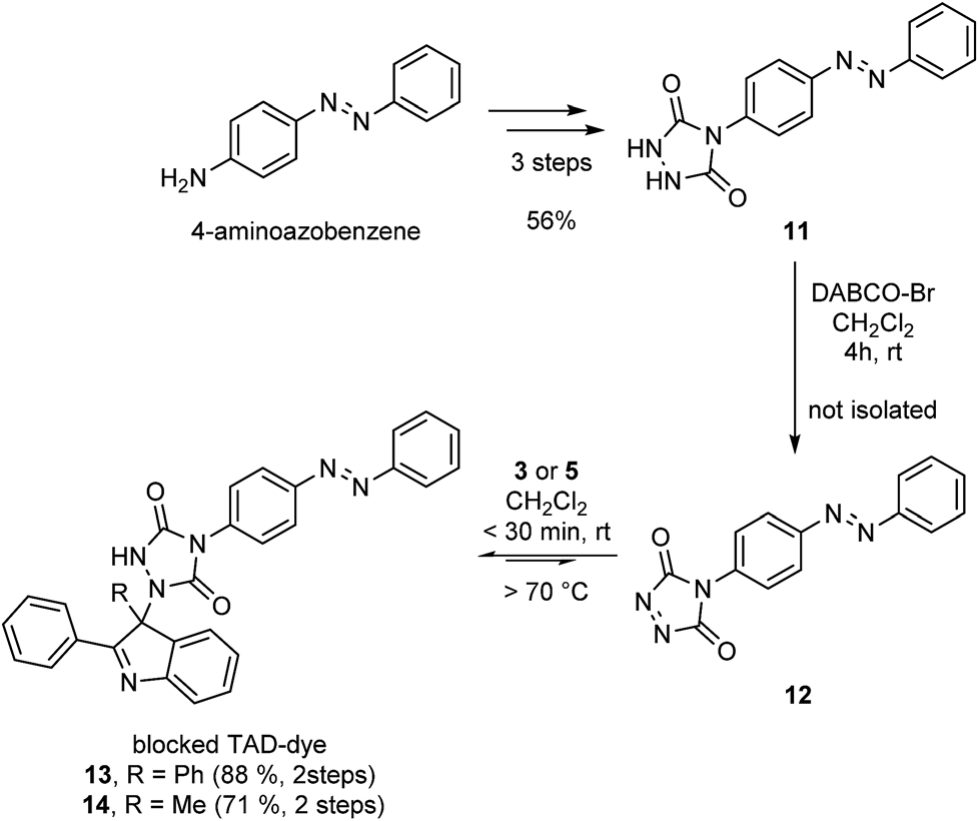
Synthesis of an azobenzene containing TAD-dye **12** and its bench-stable indole blocked derivatives **13** and **14**.

An important example of click chemistry applications involves the covalent introduction of dyes to various substrates. The synthesis of functionalized TAD compounds can be challenging,^29^ but here we introduce a novel and readily accessible dye-containing TAD reagent bearing an azobenzene-type chromophoric unit. The brightly orange 4-azobenzene urazole (**11**, Scheme 4) can be easily obtained in a three-step synthesis from commercial 4-aminoazobenzene. The obtained urazole is then oxidized and converted into the TAD-dye conjugate **13** or **14** *via* the corresponding TAD–indole click reaction.

Typically, functional TAD compounds are very reactive and can self-react over prolonged periods of time, necessitating their generation *in situ* or only just prior to use.^29^ An alternative approach can be offered by first reacting the TAD moiety with a suitable blocking agent that can be removed in a later stage.^24^ The newly developed 2-phenylindoles are attractive blocking agents for this purpose, as they give bench-stable adducts **13** and **14** (Scheme 4) that release TAD moieties in a controlled fashion upon heating, wherein the release temperature can be controlled by judiciously choosing the indole reaction partner (*cf.* Fig. S5†).

To demonstrate the practicality of the indole-blocked TAD-dye reagents, we designed a cascade of transclick reactions on low molecular weight substrates. Thus, a similar experiment as before (*cf.* Fig. 5a) was carried out in which a slight excess of receptor indole **3** was added to a DMSO-*d*_6_ solution of blocked TAD-dye **13**. Upon heating the resulting two-component mixture for 15 minutes to 120 °C, a complete and selective exchange of the TAD-dye to the least sterically hindered indole **3** is observed *via* ^1^H-NMR analysis (Fig. S6†). The resulting mixture, containing the liberated indole **5** and the newly formed blocked TAD-dye **14**, was then treated with a slight excess of HDEO and the final, irreversible transclick reaction is induced (15 minutes at 150 °C).

### Transclicking functionalized TAD compounds from and to macromolecular substrates

The blocked TAD-dyes **13** and **14** were used to functionalize a macromolecular substrate. For this, polyisoprene (*M*_n_ = 35 kDa), which offers suitable TAD-reactive sites along the entire polymer backbone, was selected. Since this polymer is not soluble in DMSO, the indole-to-ene transclick reaction was performed in deuterated chloroform to enable ^1^H-NMR analysis. Thus, the blocked TAD-dye was dissolved with polyisoprene and heated in a pressure tube (outside temperature 120 °C). For the more sterically hindered 2,3-diphenylindole blocked TAD-dye **13**, this resulted in the complete transfer of the dye onto the polymer within 40 min, whilst a longer reaction time of 105 min was needed for its 3-methyl-2-phenylindole analogue **14** to transfer all of the TAD-dye substrate to the macromolecular substrate (Fig. S7–9†). The TAD-dye from adduct **13** could also be completely transferred to polyisoprene at only 60–65 °C (by refluxing in chloroform-*d* for 24 h). Following the complete transclick reaction, the resulting functionalized and dark orange polymers were isolated by precipitation in cold methanol, while the corresponding liberated indole blocking agent was retrieved from the supernatant phase (both evidenced by ^1^H-NMR, Fig. S10†).

**Fig. 6:**
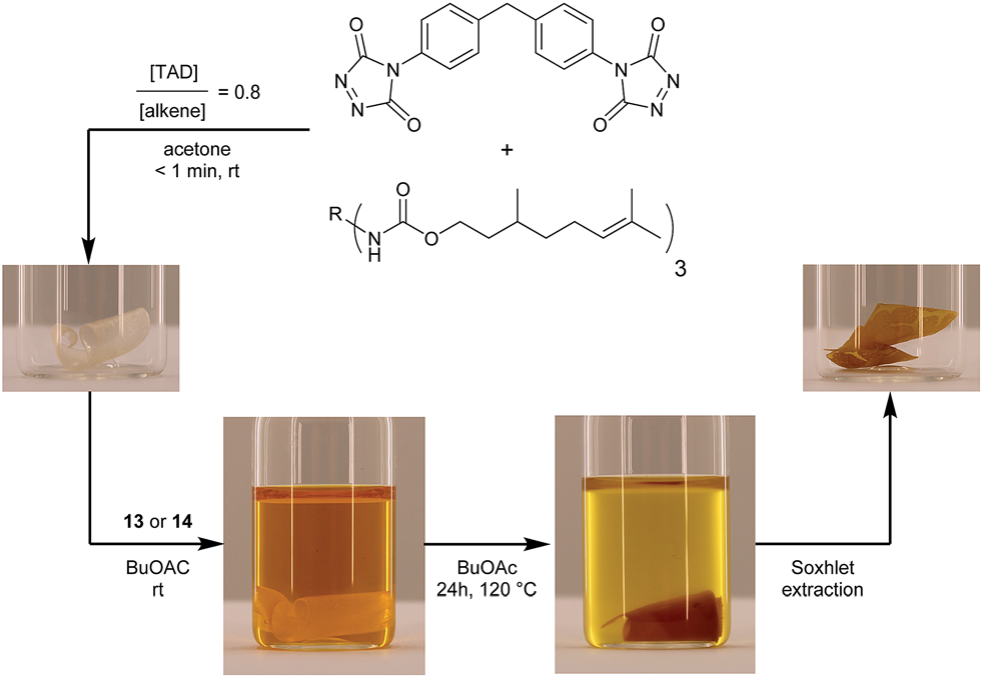
Transfer of a TAD-dye from a solution onto a network, containing residual TAD-reactive sites, upon heating. Besides a clear visual feedback, the covalent attachment of the orange dye is confirmed with a Soxhlet extraction, thus providing evidence of the successful transclick event.

Next, we designed a more challenging – and visually striking – transclick experiment in which the TAD-dye is transferred from a solution onto an insoluble but swollen polymeric resin. To provide a suitable TAD-reactive resin, a network was first synthesized by crosslinking a citronellol trimer (which is derived from an industrially available multi-isocyanate) with a bisfunctional TAD, so that an excess of residual unsaturations is guaranteed (see Fig. 6). After heating this network in a solution of the blocked TAD-dye **13** or **14**, an orange colored material was recovered, showing the covalent attachment of the initial TAD-dye, as this color persisted after a Soxhlet extraction was performed (see Fig. 6).

**Scheme 5:**
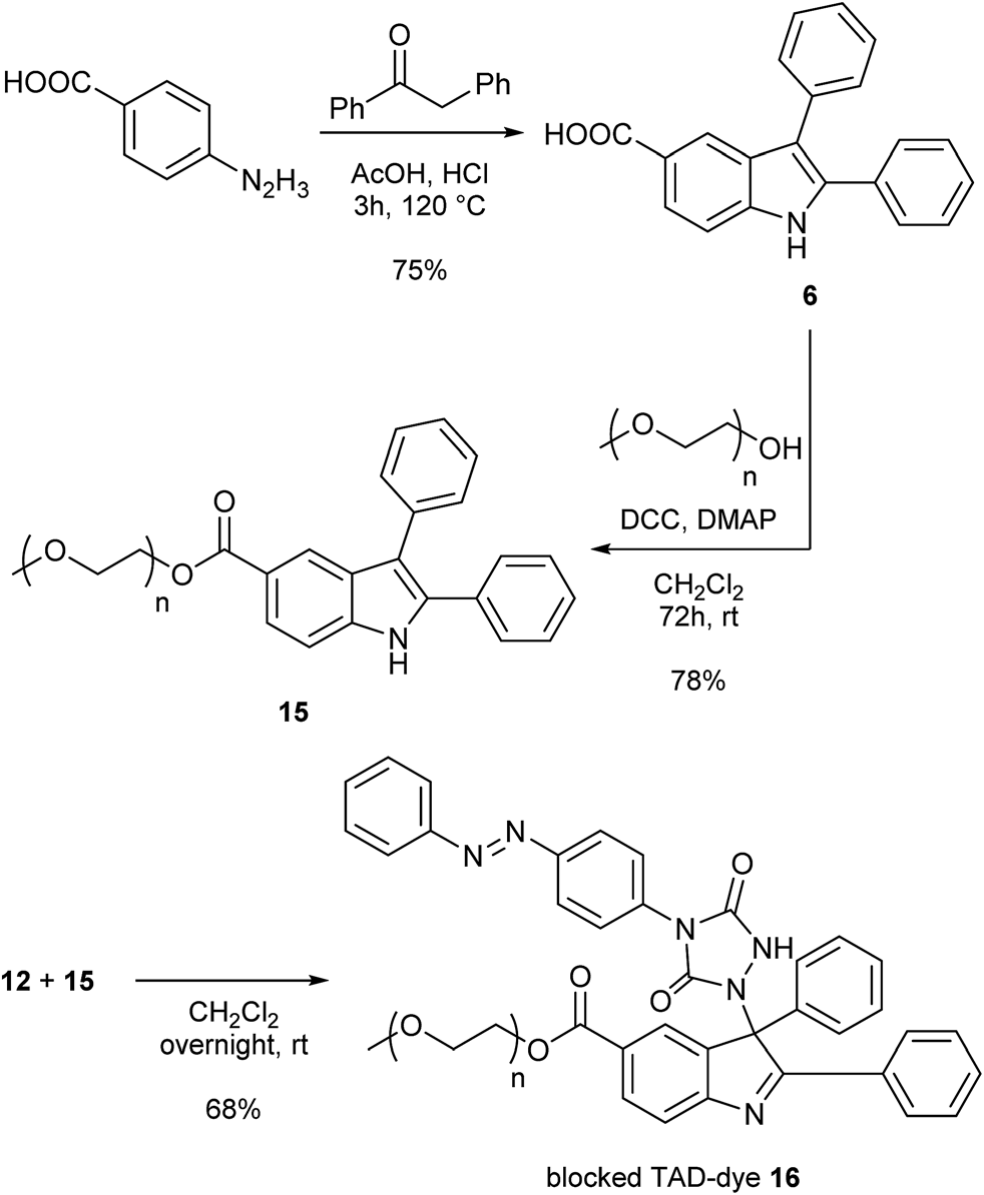
Synthesis of a 2,3-diphenylindole end-functionalized polyethylene glycol substrate (**15**) and subsequent reaction with TAD-dye **12** to give the water soluble blocked TAD-dye **16**.

**Fig. 7:**
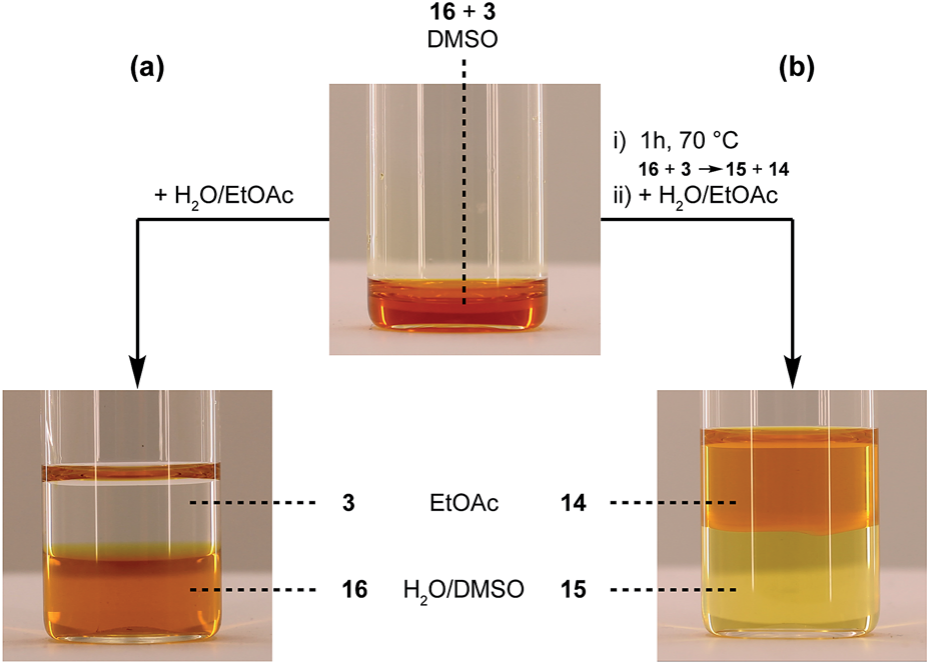
TAD-dye transclick reaction from PEG-conjugate **16** to indole **3**, visualized by the transfer of the orange dye from the water phase before (a), to the organic phase after (b) the transclick reaction.

As a final test for the TAD–indole transclick reaction on macromolecular substrates, we prepared an end-functionalized polymer from a 2,3-diphenylindole. Hence, PEG monomethyl ether (*M*_n_ = 2 kDa) was modified *via* the carboxylic acid of the 2,3-diphenylindole derivative **6** (see Scheme 5), which can be prepared from commercial building blocks in a single step. The resulting PEGylated indole **15** was then reacted with the azobenzene TAD-dye **12** to provide a water-soluble blocked TAD-dye adduct **16** (see Fig. 7a). The resulting dye–PEG conjugate **16** was next incubated in DMSO-*d*_6_ in the presence of a near-equimolar amount of 3-methyl-2-phenylindole **3** and heated at 70 °C for 1 h. ^1^H-NMR analysis of the resulting reaction mixture revealed a clean transclick product with a complete release of the PEG-supported 2,3-diphenylindole **15**, and the conjugation of the TAD-dye to the low molecular weight acceptor indole moiety **3** (Fig. S11†). The obtained reaction mixture was then added to water and phase separated by the addition of ethyl acetate, showing the preferential presence of the newly formed indole–dye conjugate **14** in the organic phase (see Fig. 7b). After extraction, the combined water layers were dried *in vacuo* and the organic phase evaporated to obtain the initial PEGylated indole blocking agent **15** and the newly formed indole-dye adduct **14**, respectively, as verified by ^1^H-NMR analysis (Fig. S12†).

## Conclusions

In this work, we showed the possibility to engineer the TAD–indole transclick platform in order to modulate the forward and backward reactivity, and thus the temperature at which the TAD–indole conjugation becomes reversible. A series of easy-to-introduce substituents were incorporated onto the indole scaffolds and their effect yielded orthogonal reactivity partners. This resulted in the identification of a novel class of upscalable and highly versatile reaction partners for TAD reagents in thermoreversible click reactions. Whilst the introduction of a phenyl group at the C2-position slightly enhances the forward reaction kinetics, the additional introduction of a phenyl group at the C3-position leads to a significant lowering of the backward reaction barriers. In this latter case, the temperature range at which the TAD–indole click reaction can be reversed to release TAD reagents is lowered by more than 20 °C. By further changing the TAD reagent from an alkyl-substituted to an aryl-substituted compound, the reversible behavior can be varied over a range of almost 50 °C, opening up transclick applications from temperatures as low as 40 °C, thus significantly expanding the scope of this reversible click chemistry.

This remarkable and predictable control of the reactivity of TAD–indole click reactions has been rationalized by DFT calculations, and the insights into the reaction mechanism point to additional ways in which the reactivity of indoles toward TADs might be further rationally enhanced or fine-tuned.

Because of the excellent control of forward and backward reaction barriers, we were also able to identify orthogonally reactive indoles that allow for an unprecedented indole-to-indole directed transclick reaction. Thus, a smooth cascade of two consecutive transclick reactions of a single TAD reagent between three different substrates was shown on low MW compounds, as well as on macromolecular substrates. From these, a newly synthesized TAD-dye reagent was transferred from a small molecule indole onto a linear polymer in solution and onto an insoluble polymer resin. The reversible blocking strategy for a functional TAD-dye was also demonstrated using a water-soluble PEG–indole that forms a dynamic conjugate.

The main limitation of the TAD–indole transclick chemistry so far, has been the access to (and synthesis of) functional TAD reagents. Although several interesting TAD compounds have been reported or are commercially available, these often require much synthetic work and expertise. Our ongoing research programs are aimed at further developing such functional and/or modular TAD building blocks, and using them for the rational design of functional and responsive materials, as well as for the design of dynamic macromolecular systems with tunable properties, that can be triggered at different temperatures.
