# Supplementary material for: Design of a thermally controlled sequence of triazolinedione-based click and transclick reactions
Source: Chem Sci. 2017 Feb 16;8(4):3098–108. doi: 10.1039/c7sc00119c (PMC5412480; doi:10.1039/c7sc00119c)
Supplement: Supplementary file 1 [file SC-008-C7SC00119C-s001.pdf]

Electronic Supplementary Material (ESI) for Chemical Science.
This journal is © The Royal Society of Chemistry 2017

Supporting Information:

Design of a thermally controlled sequence of triazolinedione-based click and transclick reactions†

Hannes A. Houck,[a,b] Kevin De Bruycker,[a] Stijn Billiet,[a] Bastiaan Dhanis,[a] Hannelore Goossens,[c] Saron Catak,[c,d] Veronique Van Speybroeck,[c] Johan M. Winne*,[a] and Filip E. Du Prez*,[a]

[a]Department of Organic and Macromolecular Chemistry, Polymer Chemistry Research Group and Laboratory for Organic Synthesis, Ghent University, Krijgslaan 281 S4-bis, 9000 Ghent, Belgium.
[b]Preparative Macromolecular Chemistry, Institut für Technische Chemie und Polymerchemie, Karlsruhe Institute of Technology (KIT), Engesserstraße 18, 76131 Karlsruhe, Germany.
[c]Center for Molecular Modeling, Ghent University, Technologiepark 903, 9052 Zwijnaarde, Belgium.
[d]Department of Chemistry, Bogazici University, 34342 Bebek, Turkey.

E-mail: johan.winne@ugent.be; filip.duprez@ugent.be

ADDITIONAL FIGURES 3
EXPERIMENTAL DETAILS 13
KINETIC REVERSIBILITY STUDIES OF TAD-INDOLE SYSTEMS 13
INDOLE-TO-INDOLE TRANSCLICK REACTION 16
TRANSCLICK REACTION OF BLOCKED TAD-DYES ON POLYISOPRENE 18
TRANSCLICK REACTION OF BLOCKED TAD-DYES ON A POLYMERIC RESIN 18
MULTI-PHASE DEMONSTRATION OF TAD-DYE TRANSCLICK EVENTS 19
SYNTHESIS OF MODEL COMPOUNDS AND POLYMERS 19
MATERIALS 19
SYNTHETIC PROCEDURES 20
Synthesis of 2-tert-butyl-3-isopentyl-1*H*-indole (1) 20
Synthesis of 3-isopentyl-2-phenyl-1*H*-indole (2) 21
Synthesis of 3-methyl-2-phenyl-1*H*-indole (3) 21
Synthesis of 3-methyl-2-phenyl-1*H*-indole-5-carboxylic acid (4) 22
Synthesis of 2,3-diphenyl-1*H*-indole (5) 22
Synthesis of 2,3-diphenyl-1*H*-indole-5-carboxylic acid (6)[7] 23
Synthesis of 2-phenyl-3-(2-phenylpropan-2-yl)-1*H*-indole (7) 23
Synthesis of 3-(1,1-diphenylethyl)-2-phenyl-1*H*-indole 24
Synthesis of 2-phenyl-3-trityl-1*H*-indole 24
Synthesis of methyl 2,3-diphenyl-1*H*-indole-5-carboxylate (8) 25
Synthesis of 4-(4-azobenzene)-1,2,4-triazoline-3,5-dione (TAD-dye) (12) 25
Synthesis of 2,3-diphenyl-1*H*-indole blocked TAD-dye (13) 26
Synthesis of 3-methyl-2-phenyl-1*H*-indole blocked TAD-dye (14) 27
Synthesis of trivalent alkene 28
Synthesis of a TAD-reactive network by crosslinking of the trivalent alkene with MDI-derived bisTAD 28
Synthesis of 2,3-diphenylindole end-functionalized PEG (15) 29
Synthesis of PEG-supported blocked TAD-dye (16) 30
COMPUTATIONAL METHODS AND THEORETICAL DATA 32
COMPUTATIONAL METHODOLOGY 32
CARTESIAN COORDINATES OF TRANSITION STATES 32
TS-1-MeTAD 32
TS-2-MeTAD 33
TS-3-MeTAD 33
TS-4-MeTAD 34
TS-5-MeTAD 34
TS-6-MeTAD 35
TS-1-MeTAD 35
TS-2-MeTAD 36
TS-3-MeTAD 36
TS-4-MeTAD 37
TS-5-MeTAD 37
TS-6-MeTAD 38
REFERENCES 38

ADDITIONAL FIGURES

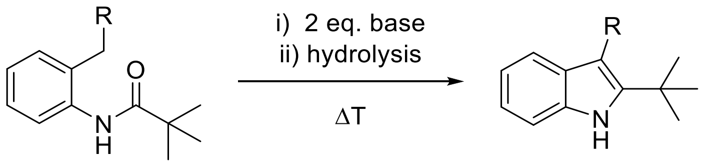

Scheme S1. Madelung synthesis of 2-*tert*-butyl-1*H*-indoles.[1]

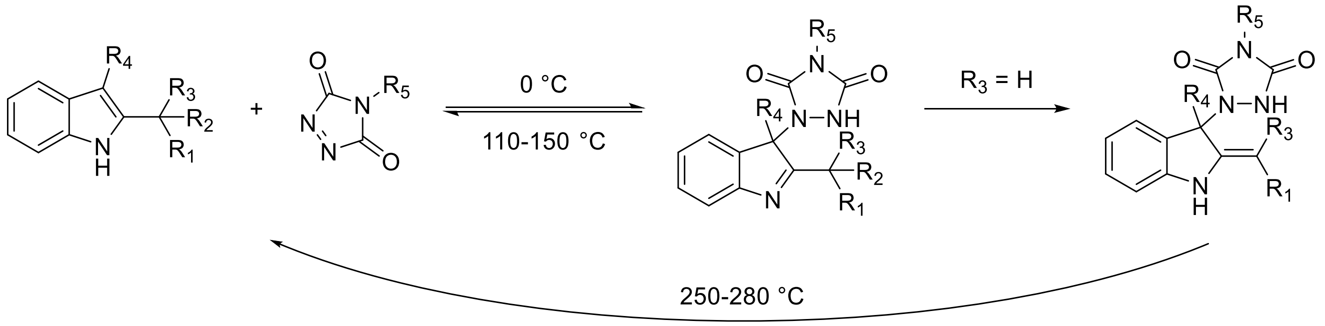

Scheme S2. Reversible click reaction between 1*H*-indole and TAD and subsequent imine-enamine tautomerization in case the indole C2-substituent has acidic protons (e.g. R_3=H). Such a substituent is undesirable, as this results in a much higher temperature of reversibility of the TAD-indole adduct.

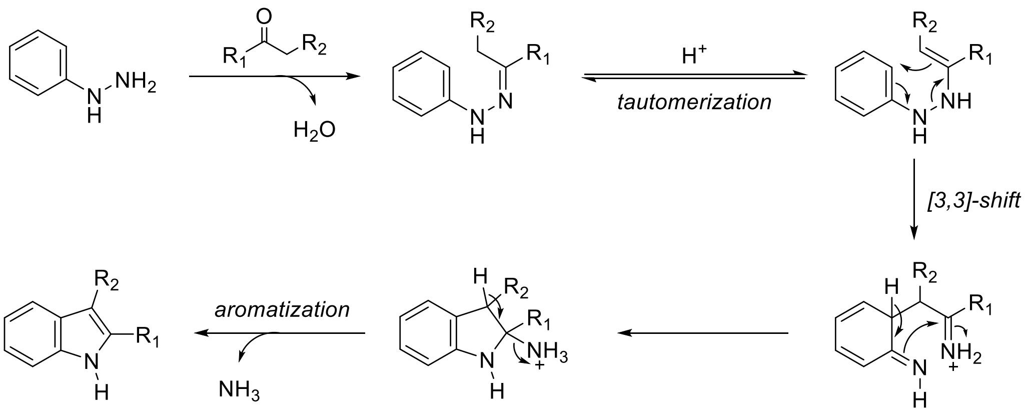

Scheme S3. Mechanism of the Fischer indolization reaction as stated by Robinson and Robinson. After tautomerization of the arylhydrazone, obtained after the first step, a [3,3]-sigmatropic rearrangement takes place. Cyclization and aromatization eventually leads to the desired (di)substituted indole reaction product.[2]

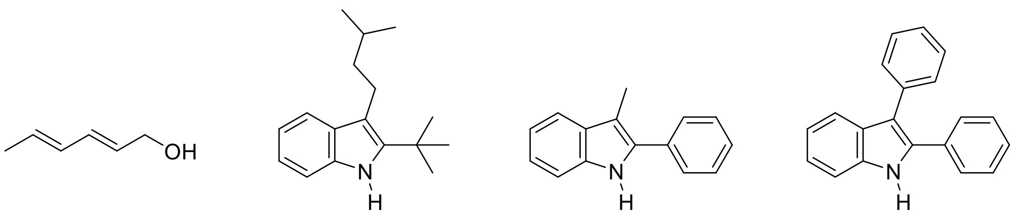

k_2 = 242[a] M^{-1} s^{-1} | k_2 = 0.434[b] M^{-1} s^{-1} | k_2 = 0.378[a] M^{-1} s^{-1} | k_2 = 0.012[a] M^{-1} s^{-1}

Figure S1. Apparent rate constants (k_2) of PhTAD click reactions at room temperature. [a]Acetone. [b]Dichloromethane.

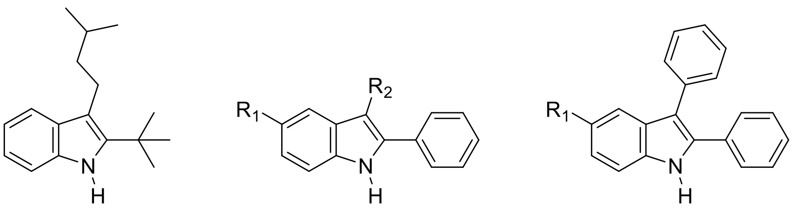

< 1 min | < 10 s | < 3 h

Figure S2. Indicative BuTAD-addition reaction times in DMSO-*d*$_6$ at room temperature (0.04 M) with the substituted indole derivatives investigated in this work, visually determined by the disappearance of the red color originating from the TAD-moiety. R_1 = H, COOH or COOMe; R_2 = Me or isopentyl.

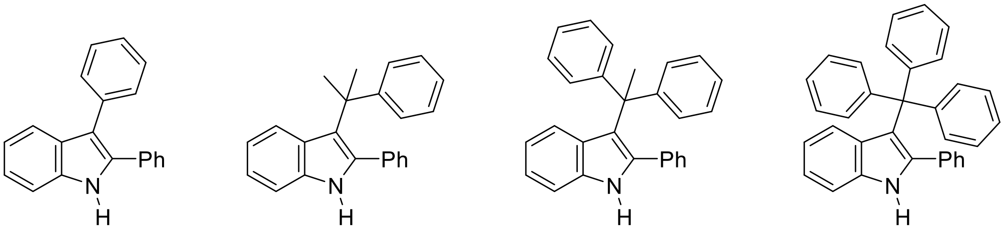

reactive | non-reactive | non-reactive | non-reactive

Figure S3. Effect of increasing steric bulk on the indole C3-position towards the TAD-addition reaction. When a tertiary substituent is present near the indole reaction site, no addition product was observed (equimolar conditions, room temperature, DMSO-*d*$_6$).

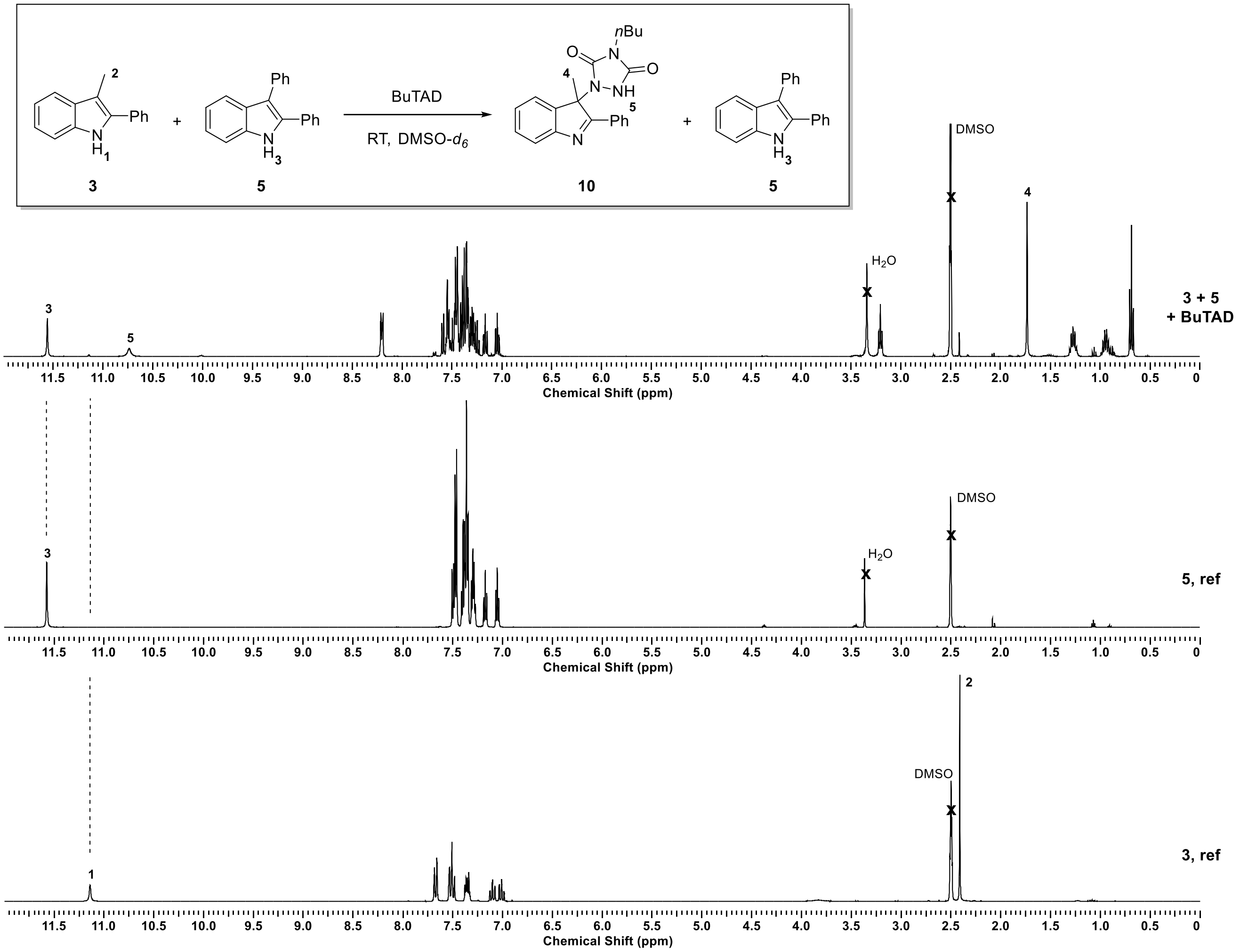

Figure S4. Competition experiment at room temperature in which an equimolar amount of BuTAD is added to a mixture containing 3-methyl-2-phenylindole (3) and 2,3-diphenylindole (5). The resulting ^{1}H-NMR spectrum (DMSO-d_6, top) shows the kinetic preference of TAD-addition to the least sterically hindered indole 3, resulting in the disappearance of the initial indole N-H signal 1 (bottom) to give the corresponding TAD-indole signal 3, whilst the other, 2,3-diphenylindole N-H signal 2 remains unaltered (middle).

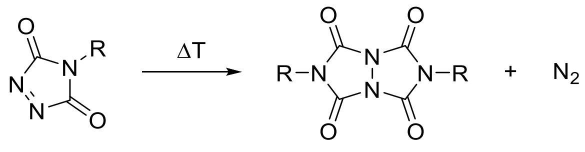

Scheme S4. Possible dimerization reaction when a TAD moiety is heated above 150 °C.[3]

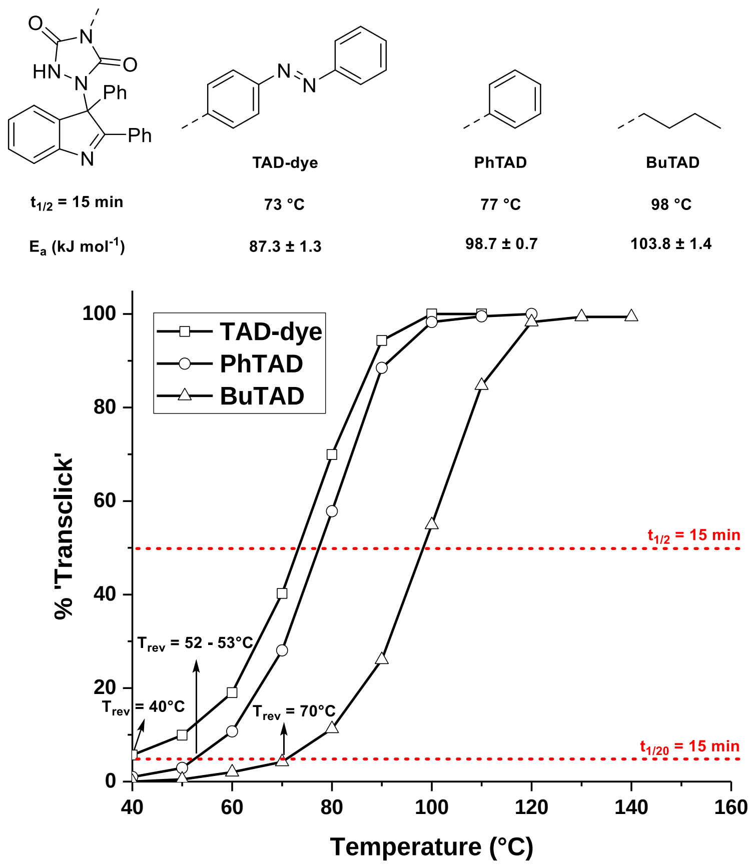

Figure S5. **Kinetic comparison of the retro-reaction of 2,3-diphenylindole conjugates with various TAD-substrates ([TAD-adduct]$_0$ = 0.04 M). The red lines represent the 5% and 50% level from which the reversibility temperature and half-life temperature are determined for each indole.**

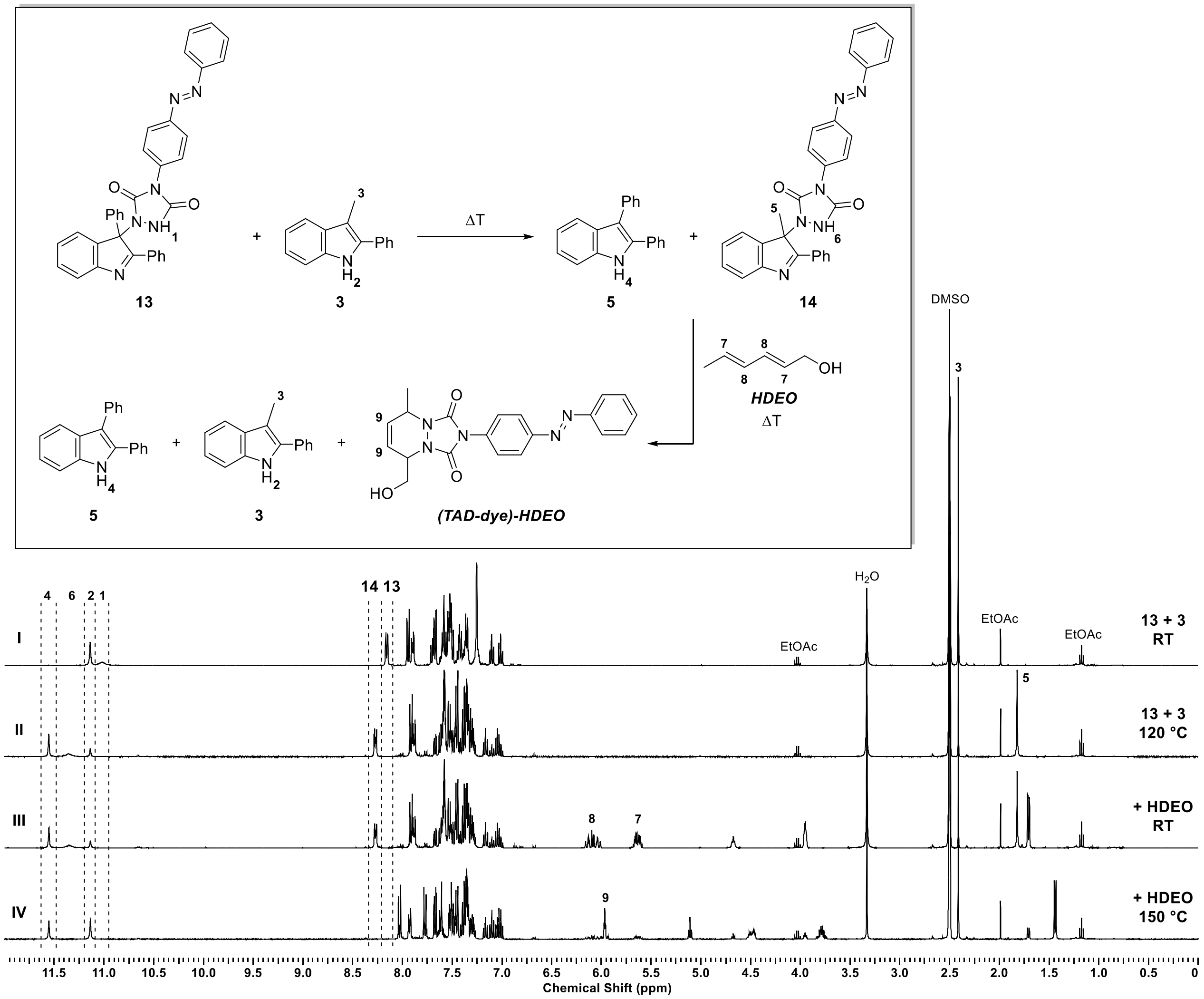

Figure S6. Indole-to-indole transclick reaction of a dye-containing TAD-moiety upon heating TAD-dye conjugate 13 in the presence of receptor indole 3. The resulting ^{1}H-NMR spectra (I and II) show the complete disappearance of the initial urazole N-H signal 1, the concomitant appearance of urazole N-H signal 6 – arising from the newly formed TAD-dye adduct 14 -, and the release of indole 5, which could be evidenced by the corresponding indole N-H signal 4. Upon addition of HDEO to the resulting mixture (spectrum III) and heating at 150 °C, the TAD-dye is finally irreversibly transferred into a Diels-Alder adduct (signal 9, IV).

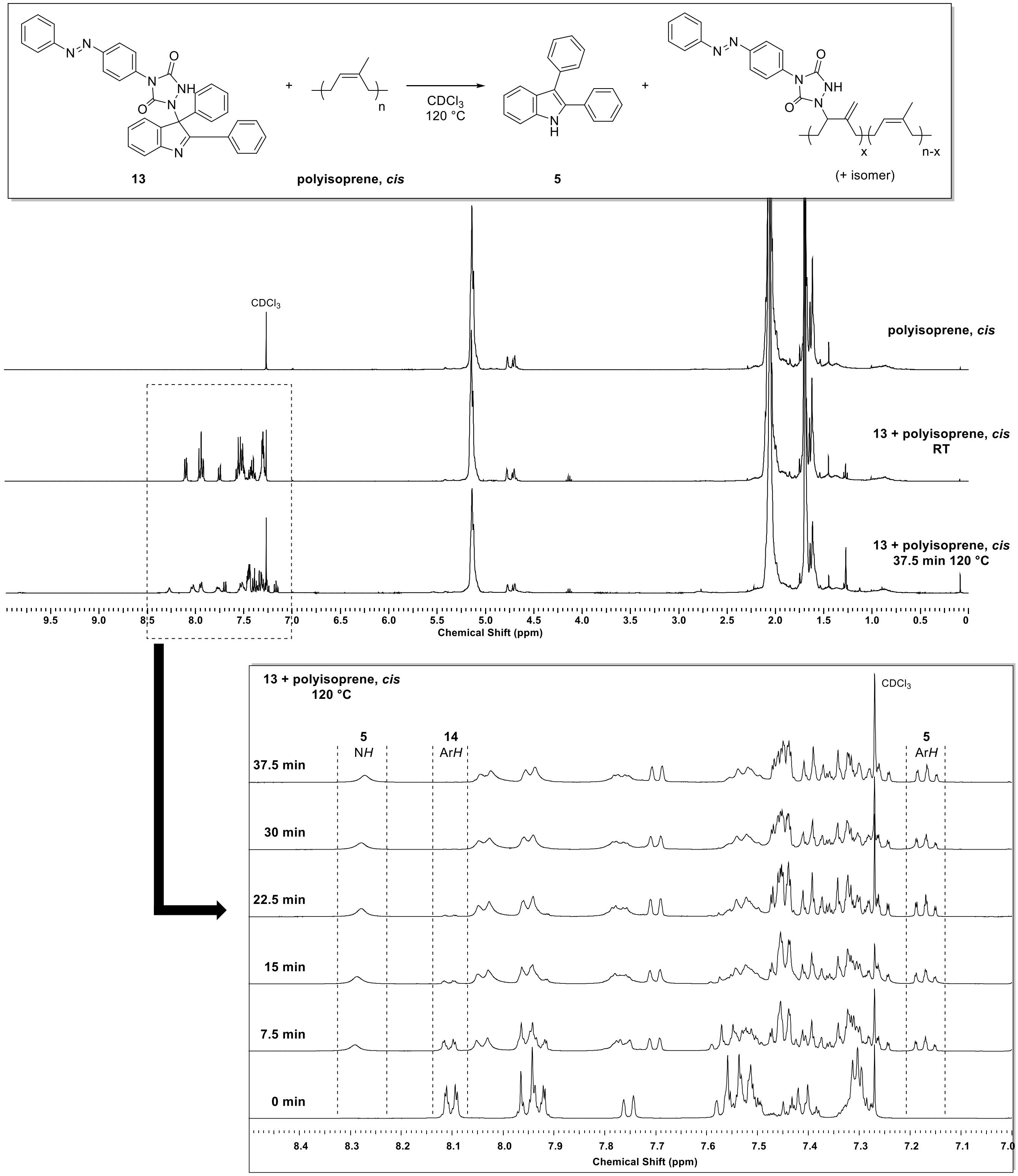

Figure S7. **TAD-dye transfer from its indole-blocked derivative 13 onto a polyisoprene backbone upon heating at 120 °C. ^{1}H-NMR analysis indicates a complete transfer within 40 minutes.**

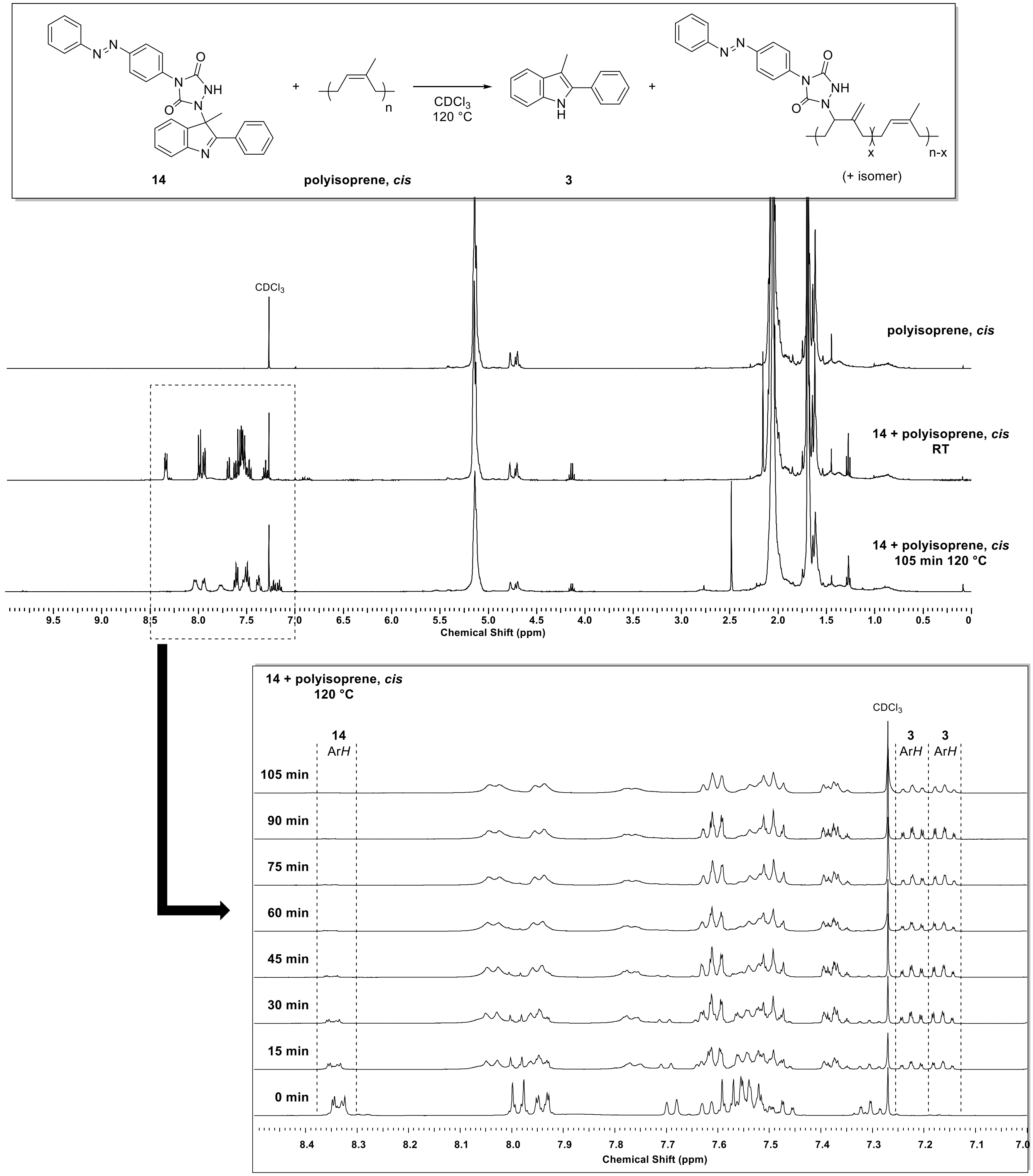

Figure S8. TAD-dye transfer from its indole-blocked derivative 14 onto a polyisoprene backbone upon heating at 120 °C. ^{1}H-NMR analysis indicates a complete transfer after 105 minutes.

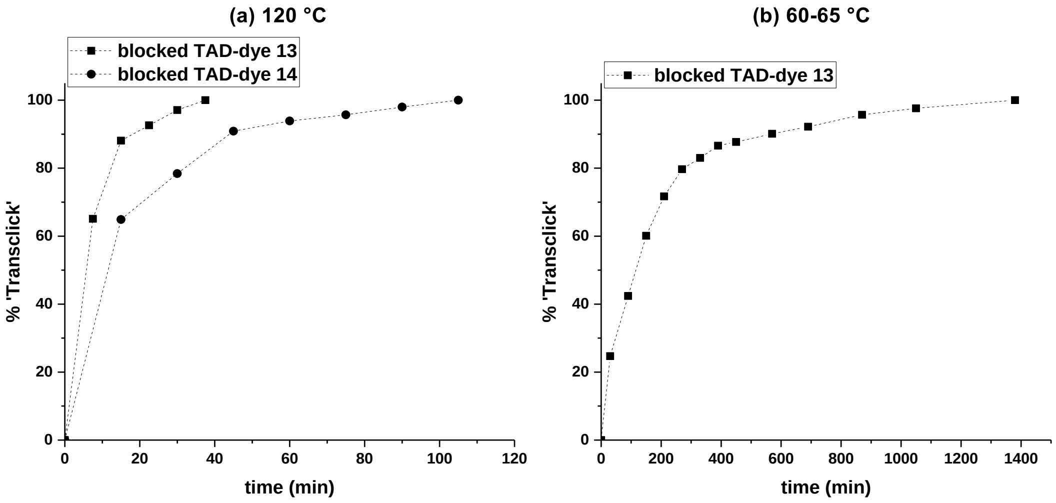

Figure S9. **(a) Transclick reaction kinetics of TAD-dye conjugate 13 or 14 with polyisoprene, upon heating a solution in deuterated chloroform at 120 °C (external temperature). (b) The lower reversible TAD-dye conjugate 13 could also be completely transferred to the polyisoprene backbone after 24h, under reflux conditions (i.e. 60-65 °C).**

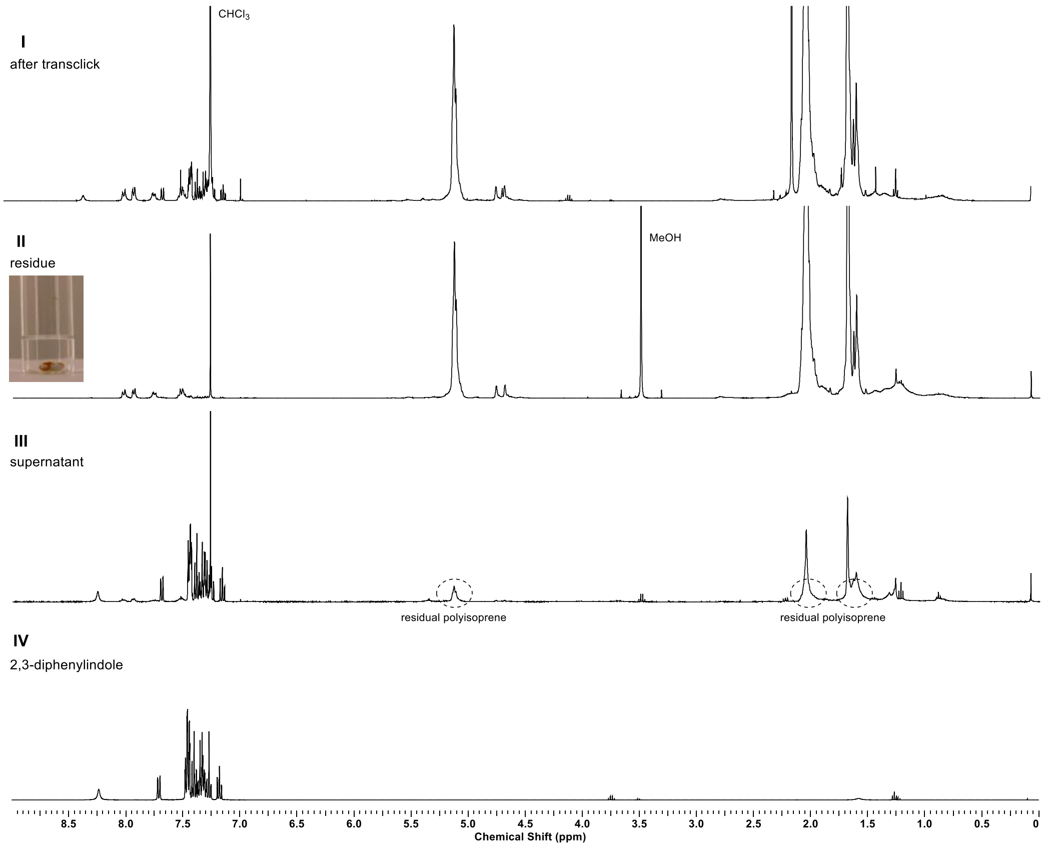

Figure S10. **Following the complete transclick reaction of TAD-dye conjugate 13 to polyisoprene (confirmed via ^{1}H-NMR, spectrum I), the reaction mixture was precipitated in cold methanol to give the dye-functionalized polyisoprene as an orange residue (evidenced by the aromatic signals in spectrum II). The supernatant after evaporation (spectrum III) was shown to contain the initial 2,3-diphenylindole blocking agent (cfr. spectrum IV as a reference), which is cleaved off during the transclick reaction.**

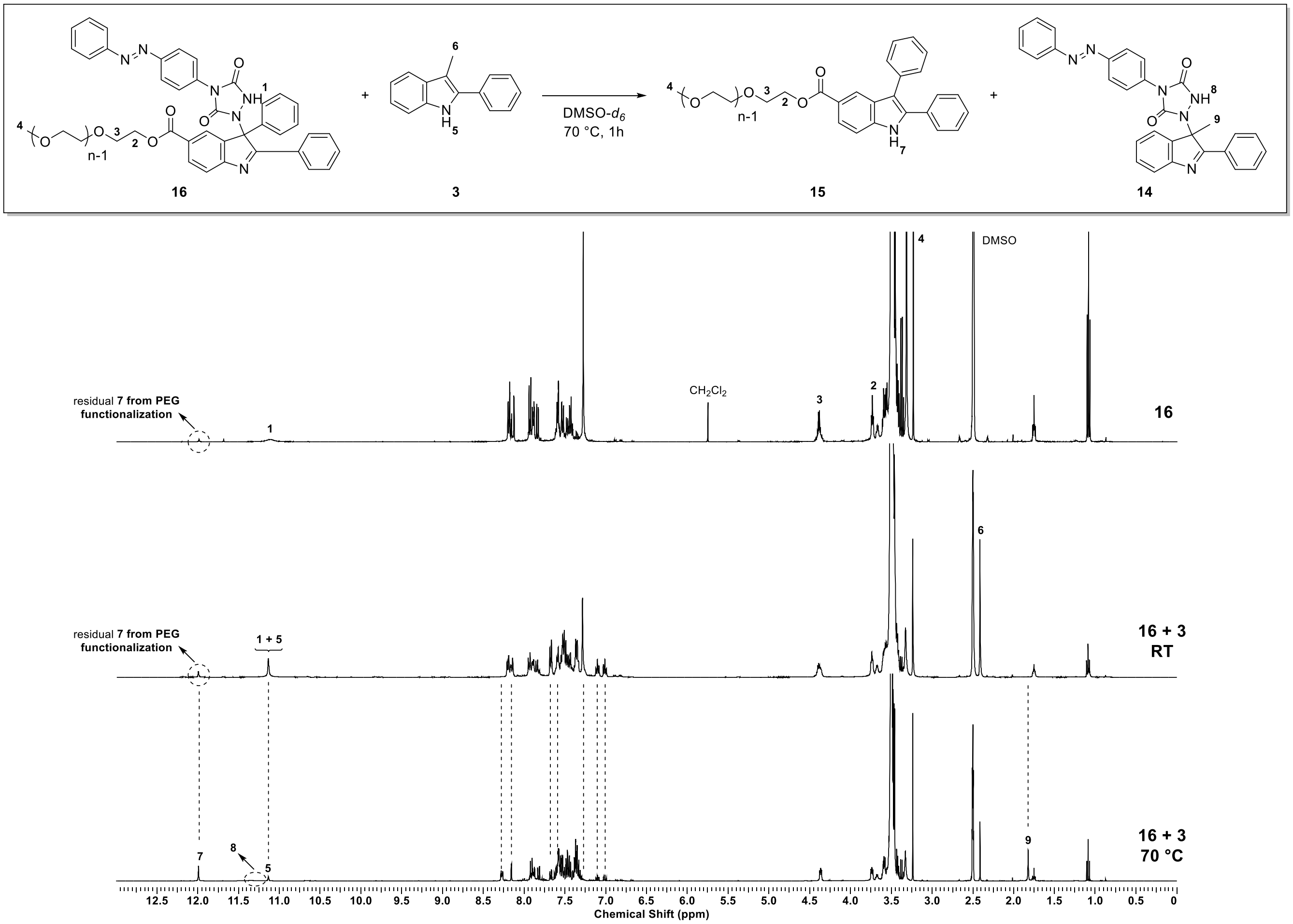

Figure S11. Heating of a solution of dye-PEG conjugate 16 in DMSO-*d*$_6$ in the presence of receptor indole 3 results in a clean transclick reaction to TAD-indole adduct 14 (cfr. signal 9) with the release of PEGylated 2,3-diphenylindole 15 (signal 7).

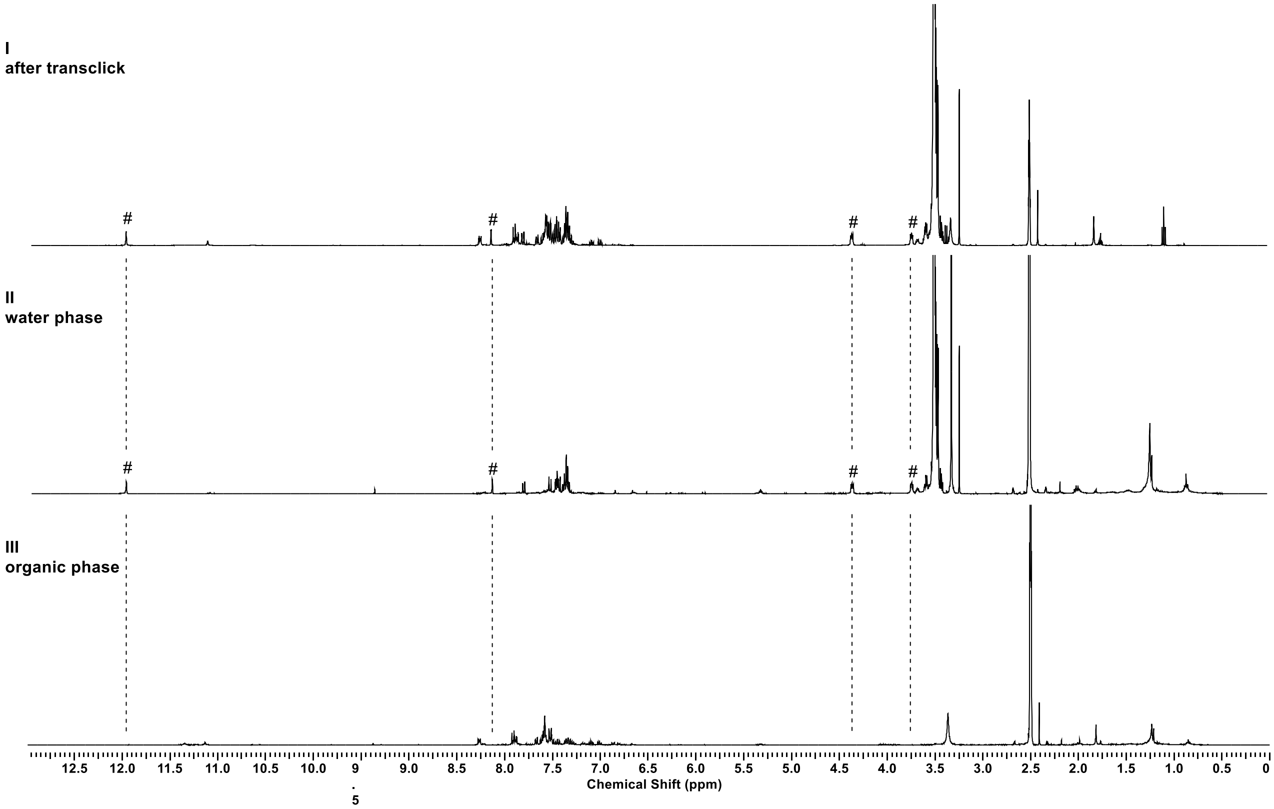

Figure S12. **^{1}H-NMR spectra after the transclick reaction between the dye-PEG-conjugate 16 and the low molecular weight indole 3 (spectrum I, some representative peaks of the PEG polymer are denoted with #). Extraction of the resulting mixture results in the separation of the water soluble PEGylated indole blocking agent 15 (spectrum II, peaks denoted with #) from the organic phase, which solely contains the newly formed indole-TAD-dye conjugate 14 (spectrum III).**

EXPERIMENTAL DETAILS

KINETIC REVERSIBILITY STUDIES OF TAD-INDOLE SYSTEMS

General protocol for the kinetic reversibility studies of indoles **1**, **2**, **3**, **5** and **8** with BuTAD, in the following illustrated for indole **3** in combination with BuTAD.

To a solution of 3-methyl-2-phenyl-1*H*-indole (**3**) (100 mg, 0.48 mmol, 1.0 eq) in 6 mL DMSO-d_6 was added an equimolar amount of a BuTAD (75 mg, 0.48 mmol, 1.0 eq) solution in DMSO-d_6 (6 mL) (Figure S13a). The resulting mixture was stirred at room temperature until the reaction went to full conversion, visually determined by the disappearance of the red TAD color and confirmed via ^{1}H-NMR spectroscopy. **3-BuTAD**: **^{1}H-NMR (400 MHz, DMSO-d_6):** δ (ppm) = 0.69 (t, 3H, CH_2-CH_3), 0.93 (m, 2H, CH_2-CH_3), 1.27 (m, 2H, N-CH_2-CH_2), 1.73 (s, 3H, C-CH_3), 3.20 (t, 2H, N-CH_2), 7.25 (t, 1H, Ar*H*), 7.38-7.46 (m, 2H, Ar*H*), 7.50-7.62 (m, 4H, Ar*H*), 8.16-8.23 (m, 2H, Ar*H*), 10.73 (s, 1H, N*H*). **^{13}C-NMR (100 MHz, DMSO-d_6):** δ (ppm) = 13.22 (CH_3), 18.77 (CH_2), 22.81 (CH_3), 28.97 (CH_2), 37.93 (CH_2), 72.33 (C), 120.88 (CH), 121.16 (CH), 126.44 (CH), 127.94 (CH), 128.67 (CH), 129.53 (CH), 131.18 (CH), 131.58 (C), 139.44 (C), 152.77 (C), 154.66 (C), 154.93 (C), 176.04 (C).

To determine the composition of the reaction mixture at elevated temperature (*vide infra*) *trans*,*trans*-2,4-hexadien-1-ol (HDEO) (52 mg, 0.55 mmol, 1.1 eq) was added after which the resulting stock solution was divided over numerous NMR samples. The corresponding NMR samples were placed in an oil bath for 15 minutes at the desired temperature (from 50 to 150 °C, in steps of 10 °C), after which the composition of the equilibrium mixture could be determined.

Via NMR, different signals could be followed to confirm a successful adduct formation at room temperature (Figure S13b). For instance, a urazole N-H signal (4) appears in the spectrum at the dispense of the original N-H indole signal (1). Furthermore, an ‘up field’ shift of the methyl protons (indole C3-substituent) can be observed upon formation of the BuTAD-indole adduct **10** (i.e. signals (2) and (6), before and after TAD reaction, respectively) and changes in the aromatic region, as well as for the butyl protons, can be detected with regard to the original indole and BuTAD spectrum. The N-methylene signal (3) of the butyl chain for instance, is clearly shifted around the typical water signal (3.33 ppm). This decrease in chemical shift is typical for BuTAD-ene reactions and can be explained by the higher electron density that the triazolinedione entity possesses after the ene-reaction.

An important limitation regarding the analysis of the reverse reaction however, is the quantitative adduct formation at room temperature that occurs with the indole substrate. As a result, fragmentation of the ene-adduct into the initial indole and TAD compounds upon heating of the reaction mixture does not give representative results. To circumvent this limitation, a slight excess of *trans*,*trans*-2,4-hexadien-1-ol (HDEO, see Figure S13a) was added to the adduct at room temperature. This diene shows fast kinetics toward TAD moieties, resulting in the irreversible formation of a Diels-Alder adduct. Thus, when the TAD-indole reaction mixture is heated for 15 minutes, the *in situ* released triazolinedione is subsequently trapped in the BuTAD-HDEO adduct by means of a transclick reaction. As a result, the amount of liberated TAD species from the retro-TAD-indole reaction is now frozen in, thereby enabling offline NMR-analysis. Indeed, as is depicted in Figure S13c, the NMR spectra obtained during the reversibility study show the disappearance of adduct **10** from the reaction mixture (signals 4 and 8) after heating. At the same time, an increase of the initial indole N-H signal (1) and a decrease of the HDEO signals (9 and 10) are observed. This results in the appearance of signals (12) of the newly formed Diels-Alder adduct (BuTAD-HDEO). Qualitative analysis of the spectra showed no retro-reaction below 90 °C, whilst heating for 15 minutes at 150 °C results in the quantitative release of indole, and thus BuTAD, moieties. Since the resulting NMR spectrum after heating at 150 °C is superimposable with

the spectrum of a reference sample in which indole **3** is added to a BuTAD-HDEO solution at room temperature, the existence of side reactions at higher temperatures could be excluded.

A similar protocol was used for the kinetic reversibility studies of indole **3** and **5** in combination with PhTAD and 4-(4-azo-benzene)-TAD-dye **12** (*vide infra*).

3-PhTAD. **^{1}H-NMR (400 MHz, DMSO-*d$_6$*):** δ (ppm) = 1.81 (s, 3H, CH_3), 7.16-7.23 (m, 2H, Ar*H*), 7.27-7.47 (m, 5H, Ar*H*), 7.52-7.64 (m, 5H, Ar*H*), 8.19-8.30 (m, 2H, Ar*H*), 11.17 (s, 1H, N*H*). **^{13}C-NMR (100 MHz, DMSO-*d$_6$*):** δ (ppm) = 22.92 (CH_3), 72.41 (C), 120.95 (CH), 121.20 (CH), 125.72 (CH), 126.72 (CH), 127.99 (CH), 128.10 (CH), 128.77 (CH), 128.89 (CH), 129.67 (CH), 130.91 (C), 131.28 (CH), 131.48 (C), 139.50 (C), 152.75 (C), 153.37 (C), 175.95 (C).

5-PhTAD. **^{1}H-NMR (400 MHz, DMSO-*d$_6$*):** δ (ppm) = 7.15-7.28 (m, 7H, Ar*H*) , 7.31-7.49 (m, 7H, Ar*H*), 7.49-7.58 (m, 2H, Ar*H*), 7.69 (d, 1H, Ar*H*), 8.13 (m, 2H, Ar*H*), 10.81 (s, 1H, N*H*). **^{13}C-NMR (100 MHz, DMSO-*d$_6$*):** δ (ppm) = 81.18 (C), 121.09 (CH), 123.42 (CH), 125.88 (CH), 126.90 (CH), 127.08 (CH), 128.11 (CH), 128.22 (CH), 128.37 (CH), 128.76 (CH), 128.80 (CH), 128.94 (CH), 130.39 (CH), 130.82 (CH), 130.91 (C), 132.20 (C), 134.87 (C), 154.52 (C), 176.16 (C).

3-TAD-dye. **^{1}H-NMR (400 MHz, DMSO-*d$_6$*)**: Major diastereomer: δ (ppm) = 1.82 (s, 3H, CH_3), 7.31 (t, 1H, Ar*H*), 7.44 (t, 1H, Ar*H*), 7.50-7.64 (m, 10H, Ar*H*), 7.85-7.94 (m, 4H, Ar*H*), 8.24-8.30 (m, 2H, Ar*H*), 11.34 (s, 1H, N*H*); Minor diastereomer, some resolved resonances: δ (ppm) = 1.77 (s, 3H, CH_3), 6.81 (m, 2H, Ar*H*), 6.87 (m, 2H, Ar*H*), 7.14 (m, 1H, Ar*H*), 8.19-8.24 (m, 2H, Ar*H*). **^{13}C-NMR (100 MHz, DMSO-*d$_6$*):** δ (ppm) = 22.96 (CH_3), 72.28 (C), 119.77 (CH), 120.40 (CH), 120.97 (CH), 121.27 (CH), 122.64 (CH), 122.93 (CH), 125.84 (CH), 126.03 (CH), 126.78 (CH), 128.02 (CH), 128.79 (CH), 128.94 (CH), 129.49 (CH), 129.70 (CH), 131.31 (CH), 131.47 (C), 131.82 (CH), 133.58 (C), 139.50 (C), 150.51 (C), 151.80 (C), 152.35 (C), 152.75 (C), 152.96 (C), 175.92 (C).

5-TAD-dye. **^{1}H-NMR (500 MHz, DMSO-*d$_6$*)**: Major diastereomer: δ (ppm) = 7.21-7.48 (m, 9H, Ar*H*), 7.49-7.64 (m, 7H, Ar*H*), 7.70 (d, 1H, Ar*H*), 7.87-7.92 (m, 2H, Ar*H*), 7.94 (d, 2H, Ar*H*), 8.16 (d, 2H, Ar*H*), 11.01 (s, 1H, N*H*); Minor diastereomer, some resolved resonances: δ (ppm) = 6.83 (d, 2H, Ar*H*), 6.90 (d, 2H, Ar*H*), 7.68 (d, 1H, Ar*H*), 8.10 (d, 2H, Ar*H*). **^{13}C-NMR (125 MHz, DMSO-*d$_6$*):** δ (ppm) = 80.91 (C), 121.11 (CH), 122.65 (CH), 122.98 (CH), 123.49 (CH), 126.25 (CH), 126.93 (CH), 127.16 (CH), 128.13 (CH), 128.41 (CH), 128.77 (CH), 128.85 (CH), 129.51 (CH), 130.43 (CH), 130.85 (CH), 131.84 (CH), 132.19 (C), 133.52 (C), 134.80 (C), 150.63 (C), 151.81 (C), 154.52 (C), 176.11 (C).

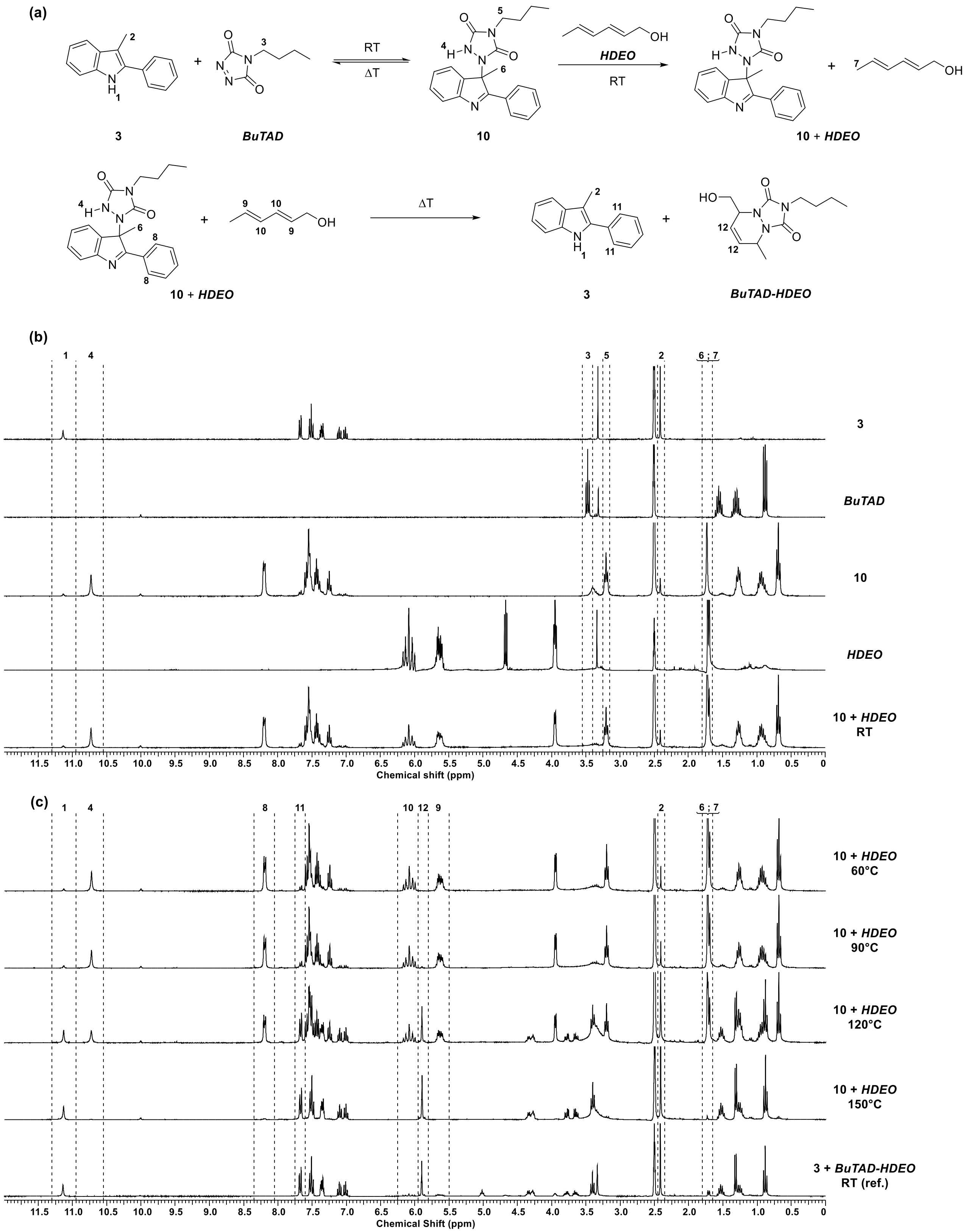

Figure S13. **(a) Reaction scheme for the kinetic reversibility study of indole 3 with BuTAD. (b) The resulting ^{1}H-NMR spectra allow for the characterization of the starting mixture via the disappearance of the indole proton signal 1 and the appearance of signal 4 from the TAD-indole adduct 10. (c) From offline NMR analysis at different temperatures, the backward TAD-indole reaction can be monitored and a reversibility profile can be obtained.**

INDOLE-TO-INDOLE TRANSCLICK REACTION

A solution of BuTAD (85.0 mg, 0.5 mmol, 1.0 eq) in dichloromethane (6 mL) was added to a solution of 2,3-diphenyl-1*H*-indole (**5**) (135 mg, 0.5 mmol, 1.0 eq) in dichloromethane (6 mL). After stirring at room temperature for 30 minutes, the reaction went to full conversion, determined by the visual disappearance of the reddish TAD color. To ensure equimolarity, a few additional drops of a BuTAD solution in dichloromethane were added to the reaction mixture until a slight pinkish color persisted. After solvent removal under reduced pressure, the obtained urazole adduct is dried in a vacuum oven overnight at 40 °C to give pure adduct **9** (210 mg – 99%). **5-BuTAD**: **^{1}H-NMR (500 MHz, DMSO-*d*$_6$):** δ (ppm) = 0.67 (t, 3H, CH_2-CH_3), 0.85 (m, 2H, CH_2-CH_3), 1.25 (m, 2H, N-CH_2-CH_2), 3.25 (t, 2H, N-CH_2), 7.13-7.19 (m, 2H, Ar*H*), 7.19-7.25 (m, 3H, Ar*H*), 7.28 (t, 1H, Ar*H*), 7.34-7.45 (m, 4H, Ar*H*), 7.50 (t, 1H, Ar*H*), 7.67 (d, 1H, Ar*H*), 8.07 (m, 2H, Ar*H*), 10.37 (s, 1H, N*H*). **^{13}C-NMR (125 MHz, DMSO-*d*$_6$):** δ (ppm) = 13.29 (CH_3), 18.63 (CH_2), 28.89 (CH_2), 38.13 (CH_2), 81.35 (C), 120.97 (CH), 123.38 (CH), 126.79 (CH), 128.07 (CH), 128.26 (CH), 128.64 (CH), 128.73 (CH), 130.23 (CH), 130.73 (CH), 132.28 (C), 135.03 (C), 139.03 (C), 154.59 (C), 154.86 (C), 156.53 (C), 176.19 (C). **LC-MS (m/z):** 425.2 $[MH]^+$. **HRMS (m/z):** *calc.*: 425.1972, *found*: 425.1970 $[MH]^+$.

The resulting adduct **9** (100 mg, 0.24 mmol, 1.0 eq) and 3-methyl-2-phenyl-1*H*-indole (**3**) (49 mg, 0.24 mmol, 1.0 eq) were then dissolved in 6 mL DMSO-*d*$_6$ (Figure S14a). The resulting solution is divided into numerous NMR samples and studied in the desired temperature range (90-140 °C, in steps of 10 °C) by heating the sample in an oil bath for 15 minutes at the temperature of interest. Comparison of the obtained reaction mixtures with the original spectra at room temperature, allowed for the characterization of the indole-to-indole transclick reaction (Figure S14b). From the resulting ^{1}H-NMR spectra, a quantitative exchange of the BuTAD moiety from indole **5** to indole **3** can be observed when the initial mixture is heated for 15 minutes at 120 °C.

In a last experiment, the final irreversible transclick reaction of the resulting mixture is evaluated. Therefore, a solution of HDEO (26 mg, 0.26 mmol, 1.1 eq) in 1.5 mL DMSO-*d*$_6$ is added and the mixture is heated at 150 °C for 15 minutes. Again, offline ^{1}H-NMR spectroscopy allowed for the determination of the composition of the resulting reaction mixture, showing a quantitative BuTAD-HDEO adduct formation, together with the recovery of both initial indole substrates **3** and **5** (Figure S14b).

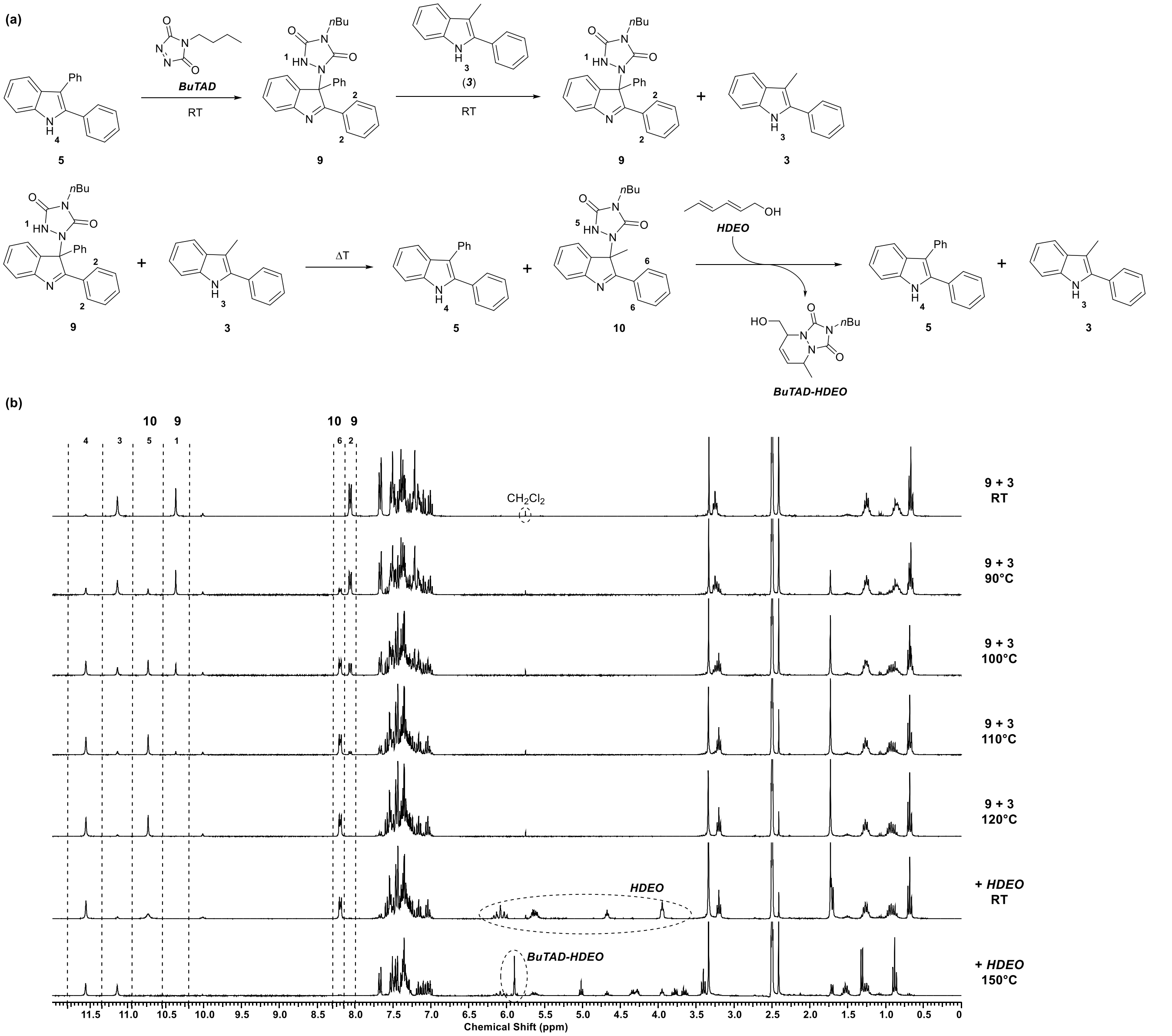

Figure S14. (a) Selective exchange of the TAD-indole adducts when a reaction mixture of adduct 9 and indole 3 is heated, (b) ^{1}H-NMR spectra after heating show the exchange of the indole N-H and urazole N-H signals (e.g. 3 for 4 and 1 for 5, respectively). Finally, 15 minutes of heating at 150 °C in the presence of HDEO in the end releases indole 3 which is evidenced by the reappearing indole N-H signal 3.

TRANSCLICK REACTION OF BLOCKED TAD-DYES ON POLYISOPRENE

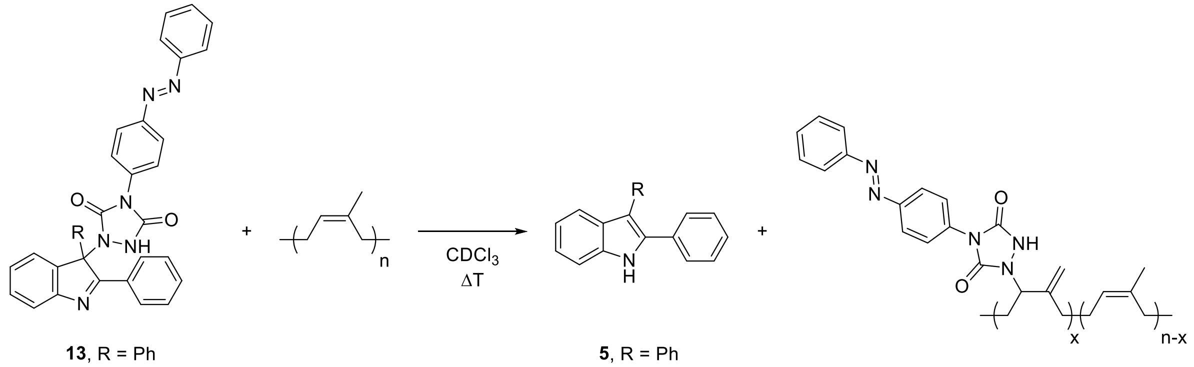

13, R = Ph
14, R = Me

5, R = Ph
3, R = Me

Polyisoprene, cis (40 mg, 1.1 .10^{-6} mol) and indole blocked TAD-dye **13** (10 mg, 2.1 .10^{-5} mol, 25 wt%) or **14** (10 mg, 1.8 .10^{-5} mol, 25 wt%) were solubilized in 0.75 mL $CDCl_3$ and transferred into a pressure tube. The resulting orange solution was then placed in a preheated oil bath at 120 °C (or 70 °C). After certain time intervals, the pressure tube was taken out of the oil bath and cooled under running tap water before subjected to ^{1}H-NMR analysis (see Figure S9). The same sample was then heated again in the oil bath and the procedure was repeated until the transclick reaction has gone to completion (confirmed by ^{1}H-NMR). The resulting solution was added to a 10-fold excess of cold methanol to give an orange precipitate, which was separated from the light yellow supernatant by decantation. The resulting orange residue was dried overnight in a vacuum oven and submitted to ^{1}H-NMR analysis, showing the covalent attachment of the TAD-dye to the polymer (see Figure S 10). The light yellow supernatant was evaporated *in vacuo*, dried in a vacuum oven overnight and analyzed by ^{1}H-NMR spectroscopy, thereby showing the presence of the released indole **5** or **3**.

TRANSCLICK REACTION OF BLOCKED TAD-DYES ON A POLYMERIC RESIN

A solution of indole blocked TAD-dye **13** (6.2 mg, 0.011 mmol) or **14** (5.2 mg, 0.011 mmol) in 10 mL butyl acetate was added to a recipient containing 70 mg of the TAD-reactive network (for synthesis of the network, see p34). The resulting orange mixture was heated in an oil bath at 120 °C for 24h. The orange polymeric resin was then taken out of the mixture and subjected to a Soxhlet extraction (ethyl acetate, 4h) to give a dark orange network, thus demonstrating the covalent attachment of the TAD-dye onto the resin. The remaining light orange mixture was then treated several times (3-5) with extra network (70 mg) to finally give a light yellow clear solution.

MULTI-PHASE DEMONSTRATION OF TAD-DYE TRANSCLICK EVENTS

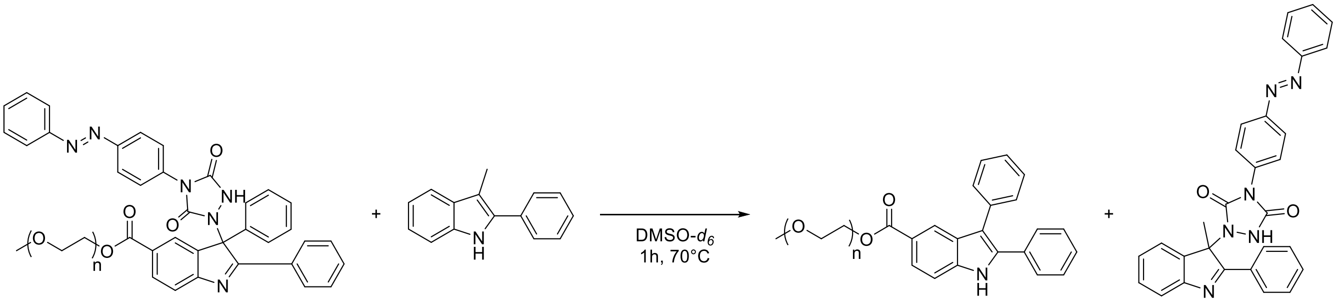

blocked TAD-dye **16** **3** **15** **14**

TAD-dye conjugate **16** (50 mg, 0.019 mmol, 1.0 eq.), obtained from the TAD-indole reaction of PEG-supported 2,3-diphenyl-indole **15** with TAD-dye **12** (*vide infra*), and 3-methyl-2-phenyl-1*H*-indole (**3**) (4,4 mg, 0.021 mmol, 1.1 eq.) were dissolved in 0.75 mL DMSO-d_6. The resulting solution was heated for 1h at 70 °C after which complete transfer of the TAD-dye was confirmed via ^{1}H-NMR spectroscopy. After cooling to room temperature, 2 mL water was added to the mixture to give an orange-yellow mixture after shaking, followed by 2.5 mL of ethyl acetate. The resulting mixture was phase separated with a centrifuge (4500 rpm, 5 min, 20 °C), showing a clear orange organic phase and a light yellow aqueous phase. The aqueous phase was taken up with a syringe to separate both layers. The resulting organic phase was washed with 1 mL water, dried on magnesium sulfate and evaporated to dryness. The resulting dark orange oil was submitted to ^{1}H-NMR analysis, which showed the presence of the newly formed TAD-dye conjugate **14** (see Figure S12). The light yellow water phase was dried *in vacuo* to give a yellow residue, containing the initial PEGylated indole blocking agent **15**, as was evidenced from the NMR-spectrum (Figure S12).

SYNTHESIS OF MODEL COMPOUNDS AND POLYMERS

MATERIALS

o-Toluidine (98%, Sigma-Aldrich), triethylamine (99%, Sigma-Aldrich), trimethylacetyl chloride (99%, Sigma-Aldrich), hydrochloric acid (36%, Chem-Lab), sodium bicarbonate (99%, Roth), *n*-butyllithium (2.5 M in hexane, Sigma-Aldrich), ammonium chloride (99%, Roth), magnesium sulfate (anhydrous, Boom), trifluoroacetic acid (99%, Sigma-Aldrich), palladium on activated charcoal (5% Pd basis, Sigma-Aldrich), 3-methylbutyraldehyde (98%, Sigma-Aldrich), celite (Sigma-Aldrich), 2-phenyl-1*H*-indole (>98%, TCI Europe), phenylhydrazine (97%, Sigma-Aldrich), propiophenone (99%, Sigma-Aldrich), sulfuric acid (99%, Sigma-Aldrich), acetic acid (>99.5 %, Sigma-Aldrich), 4-hydrazino benzoic acid (97%, Sigma-Aldrich), 2-phenylacetophenone (97%, Sigma-Aldrich), 2-phenyl-2-propanol (97%, Sigma-Aldrich), *p*-toluenesulfonic acid monohydrate (97.5%, Acros), 1,1-diphenylethanol (98%, Sigma-Aldrich), triphenylmethanol (97%, Acros), triphosgene (98%, TCI Europe), 4-aminoazobenzene (98%, TCI Europe), ethyl carbazate (97%; Acros Chemicals), potassium hydroxide (99%, Roth), citronellol (95%, Sigma-Aldrich), dibutyltin dilaurate (95%, TCI Europe), PEG monomethyl ether 2000 (Sigma-Aldrich), 4-(dimethylamino)pyridine (>99%; Sigma-Aldrich), *N*,*N*'-dicyclohexylcarbodiimide (>99%, Sigma-Aldrich), SEC Bio-Beads S-X1 Support (Bio-Rad), *trans*,*trans*-2,4-hexadien-1-ol (>97%, Sigma-Aldrich), polyisoprene 35000 (cis, Sigma Aldrich), DMSO-d_6 (Euriso-top), chloroform-*d* (Euriso-top).

All solvents (Sigma-Aldrich) and products were used without any pre-treatment or purification.

Desmodur XP 2489 polyisocyanate (21% isocyanate content) was kindly provided by Bayer MaterialScience and used as received.

DABCO-Br,[4] BuTAD,[5] PhTAD[6] and MDI-derived bisTAD[5] were synthesized according to literature procedures.

SYNTHETIC PROCEDURES

Synthesis of 2-*tert*-butyl-3-isopentyl-1*H*-indole (1)

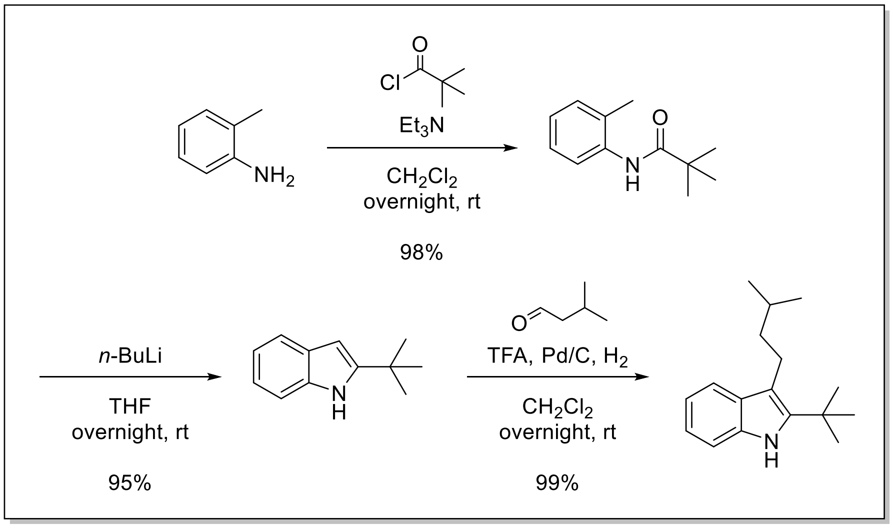

To a solution of *o*-toluidine (30.0 g, 0.280 mol, 1.0 eq.) in 240 mL dichloromethane was added triethylamine (31.2 g, 0.308 mol, 1.1 eq.) and the mixture was cooled in a water bath. To this, trimethylacetyl chloride (37.9 mL, 0.308 mol, 1.1 eq.) was added dropwise. The resulting mixture was stirred overnight under inert atmosphere and subsequently washed with 5 v/v% aqueous HCl-solution (360 mL), saturated aqueous sodium bicarbonate solution (360 mL) and brine (360 mL). After drying on magnesium sulfate *N*-*o*-tolylpivalamide was obtained by removing the organic solvent under reduced pressure (52.4 ivory white powder – 98%). Next, a solution of *N*-*o*-tolylpivalamide (35.0 g, 0.183 mol, 1.0 eq.) in dry THF (120 mL) is placed under inert atmosphere. After cooling in a water bath, *n*-butyllithium (2.5 M in hexane, 220 mL, 0.55 mol, 3.0 eq.) is added dropwise. The reaction mixture is stirred overnight at room temperature, before cooling it in an ice bath. Once cooled, saturated aqueous ammonium chloride solution (340 mL) is added slowly. The water phase is extracted with ethyl acetate (340 mL), after which the combined organic phases are dried on magnesium sulfate and concentrated *in vacuo* to obtain 2-*tert*-butyl-1*H*-indole as a brown solid (30.0 g – 95%). In a final step, a mixture of trifluoroacetic acid (2.30 mL, 30.0 mmol, 1.5 eq.), palladium (5% on activated carbon, 0.30 g) and dichloromethane (40 mL) was put under hydrogen atmosphere and cooled in an ice bath. To this, a solution of 2-*tert*-butyl-1*H*-indole (3.46 g, 20.0 mmol, 1 eq.) and 3-methylbutyraldehyde (1.90 g, 22.0 mmol, 1.1 eq.) in dichloromethane (60 mL) was added dropwise. The mixture was stirred overnight in a water bath, regularly flushing with hydrogen gas. The reaction was monitored via thin layer chromatography (TLC, hexane:ethyl acetate 9:1) until complete consumption of the indole substrate. The mixture was filtered over celite and washed with saturated aqueous sodium bicarbonate solution (100 mL). The organic phases are dried over magnesium sulfate and concentrated *in vacuo* to obtain the title compound **1** (4.83 g – 99 %). **^{1}H-NMR (500 MHz, $CDCl_3$):** δ (ppm) = 0.93 (d, 6H, CH-(C*H*$_3$)$_2$), 1.37 (s, 9H, C(C*H*$_3$)$_3$), 1.47 (m, 2H, *i*-Pr-C*H*$_2$), 1.65 (m, 1H, C*H*-(CH$_3$)$_2$), 2.76 (m, 2H, C=C-C*H*$_2$), 7.00 (m, 2H, Ar*H*), 7.18 (d, 1H, Ar*H*), 7.42 (d, 1H, Ar*H*), 7.70 (s, 1H, N*H*). **^{13}C-NMR (125 MHz, $CDCl_3$):** δ (ppm) = 22.69 (CH_3), 23.43 (CH_2), 28.87 (CH), 30.59 (CH_3), 32.95 (C), 40.81 (CH_2), 110.33 (CH), 111.36 (C), 118.16 (CH), 118.94 (CH), 120.97 (CH), 129.83 (C), 134.07 (C), 141.6 (C). **HRMS (m/z):** *calc.*: 244.2060, *found*: 244.2067 $[MH]^+$.

Synthesis of 3-isopentyl-2-phenyl-1*H*-indole (2)

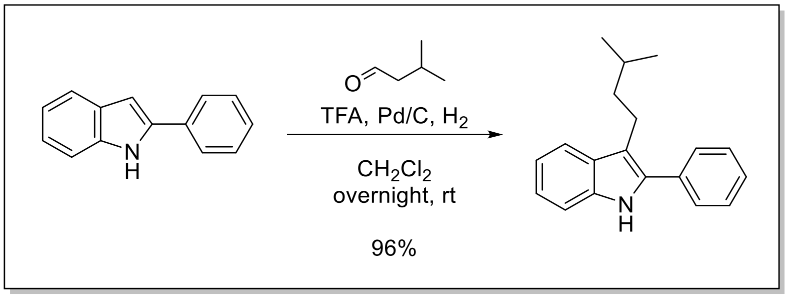

A mixture of trifluoroacetic acid (0.60 mL, 7.76 mmol, 1.5 eq.) and palladium (5% on activated carbon, 0.08 g) in 12 mL dichloromethane was placed under hydrogen atmosphere and stirred in an ice bath. To this, a solution of 2-phenyl-1*H*-indole (1.00 g, 5.17 mmol, 1.0 eq.) and 3-methylbutyraldehyde (0.61 mL, 5.69 mmol, 1.1 eq.) in 18 mL dichloromethane was added dropwise. The mixture was stirred vigorously in a water bath overnight, regularly flushing with hydrogen gas. With the aid of TLC (heptane:ethyl acetate 9:1, R_F = 0.20) the reaction was monitored until completion. After filtration over celite and washing with saturated aqueous sodium bicarbonate solution (30 mL), the organic phases are dried over magnesium sulfate and concentrated *in vacuo* to obtain the title compound **2** as a brown oil (1.34 g – 96 %). **^{1}H-NMR (300 MHz, DMSO-*d$_6$*):** δ (ppm) = 0.92 (d, 6H, C*H*$_3$), 1.55 (m, 2H, *i*-Pr-C*H*$_2$), 1.62 (m, 1H, C*H*-(CH$_3$)$_2$), 2.84 (t, 2H, C*H*$_2$-CH$_2$-*i*-Pr), 7.00 (t, 1H, Ar*H*), 7.10 (t, 1H, Ar*H*), 7.36 (m, 2H, Ar*H*), 7.50 (m, 3H, Ar*H*), 7.62 (m, 2H, CH$_2$-C=C-C=C*H*), 11.11 (s, 1H, N*H*). **LC-MS (m/z):** 264.2 $[MH]^+$. **HRMS (m/z):** *calc.*: 264.1747, *found*: 264.1735 $[MH]^+$.

Synthesis of 3-methyl-2-phenyl-1*H*-indole (3)

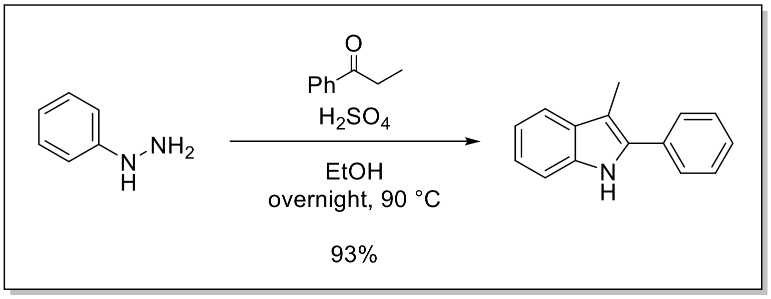

A mixture of phenylhydrazine (2.55 mL, 0.026 mol, 1.0 eq.), propiophenone (3.45 mL, 0.026 mol, 1.0 eq.) and concentrated sulfuric acid (2.77 mL, 0.052 mol, 2.0 eq.) in 100 mL ethanol was placed under inert atmosphere and heated to reflux at 90 °C. The reaction was monitored by TLC (heptane:ethyl acetate 9:1, R_F = 0.23). After completion, the reaction mixture was precipitated in a 5-fold excess of ice in water and filtered off. The obtained residue was recrystalized from water:ethanol 1:2 to give 3-methyl-2-phenyl-1*H*-indole (**3**) as greenish needles (5.02 g – 93 %). **^{1}H-NMR (500 MHz, DMSO-*d$_6$*):** δ (ppm) = 2.41 (s, 3H, C*H*$_3$), 7.01 (t, 1H, Ar*H*), 7.11 (t, 1H, Ar*H*), 7.35 (t, 2H, Ar*H*), 7.51 (t, 3H, Ar*H*), 7.67 (dd, 2H, Ar*H*), 11.15 (s, 1H, N*H*). **^{13}C-NMR (500 MHz, DMSO-*d$_6$*):** δ (ppm) = 9.82 (CH$_3$), 106.74 (C), 110.99 (CH), 118.39 (CH), 118.54 (CH), 121.52 (CH), 126.94 (CH), 127.46 (CH), 128.68 (CH), 129.36 (C), 133.06 (C), 133.71 (C), 135.90 (C). **LC-MS (m/z):** 208.1 $[MH]^+$. **HRMS (m/z):** *calc.*: 208.1121, *found*: 208.1113 $[MH]^+$.

Synthesis of 3-methyl-2-phenyl-1*H*-indole-5-carboxylic acid (4)

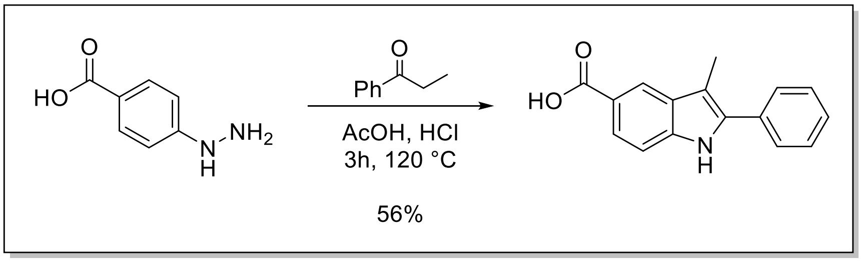

A mixture of 50 mL concentrated acetic acid and 15 mL concentrated hydrochloric acid was added to 4-hydrazino benzoic acid (1.00 g, 6.57 mmol, 1.0 eq.) and propiophenone (0.87 mL, 6.57 mmol, 1.0 eq.). The mixture was refluxed (at 120 °C) for 3 hours (monitored by TLC, heptane:ethyl acetate 9:1, R_F = 0.23) and then cooled to room temperature. By slow addition of water (60 mL), the title compound precipitated out of solution. The pure indole **4** was obtained by filtration and thoroughly dried overnight in a vacuum oven to yield a green powder (0.92 g, 56%). **^{1}H-NMR (500 MHz, DMSO-*d₆*):** δ (ppm) = 2.44 (s, 3H, C*H*$_3$), 7.35-7.43 (m, 2H, Ar*H*), 7.53 (t, 2H, Ar*H*), 7.65-7.71 (m, 2H, Ar*H*), 7.74 (d, 1H, Ar*H*), 8.21 (s, 1H, HOOC-C-C*H*-C), 11.54 (s, 1H, N*H*), 12.40 (s, 1H, COO*H*). **^{13}C-NMR (125 MHz, DMSO-d_6):** *δ* (ppm) = 9.62 (CH_3), 108.06 (C), 110.70 (CH), 121.07 (CH), 121.13 (C), 122.85 (CH), 127.40 (CH), 127.62 (CH), 128.76 (CH), 128.88 (C), 132.45 (C), 135.27 (C), 138.38 (C), 168.37 (C). **LC-MS (m/z):** 252.1 $[MH]^+$.

Synthesis of 2,3-diphenyl-1*H*-indole (5)

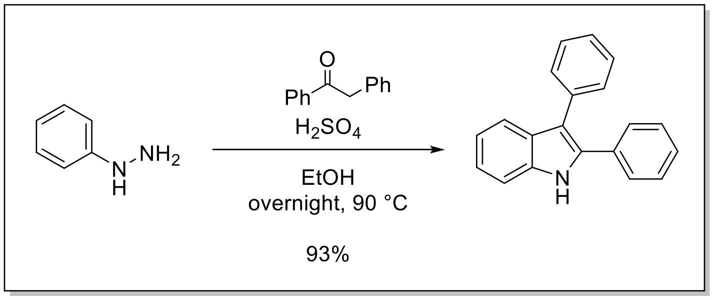

A mixture of phenylhydrazine (10.2 mL, 0.10 mol, 1.0 eq.), 2-phenylacetophenone (20.3 g, 0.10 mol, 1.0 eq.) and concentrated sulfuric acid (11 mL, 0,21 mol, 2.0 eq.) in 100 mL ethanol was placed under inert atmosphere and heated to reflux at 90 °C. The reaction was monitored by TLC (heptane:ethyl acetate 9:1, R_F = 0.30). After completion, the resulting mixture was pre-cipitated in a 5-fold excess of ice in water and filtrated. The residue was recrystalized from water:ethanol 1:2 to give 2,3-diphenyl-1*H*-indole (**5**) as brown crystals (25.94 g – 93%). **^{1}H-NMR (500 MHz, DMSO-d_6):** *δ* (ppm) = 7.04 (t, 1H, Ar*H*), 7.16 (t, 1H, Ar*H*), 7.25-7.44 (m, 8H, Ar*H*) 7.45-7.51 (m, 4H, Ar*H*), 11.56 (s, 1H, N*H*). **^{13}C-NMR (125 MHz, DMSO-d_6):** *δ* (ppm) = 111.40 (CH), 113.21 (C), 118.48 (CH), 119.61 (CH), 121.89 (CH), 125.95 (CH), 127.38 (CH), 127.89 (C), 128.08 (CH), 128.37 (CH), 128.51 (CH), 129.66 (CH), 132.39 (C), 133.99 (C), 135.21 (C), 136.02 (C). **LC-MS (m/z):** 270.1 $[MH]^+$. **HRMS (m/z):** *calc.*: 270.1277, *found*: 270.1285 $[MH]^+$.

Synthesis of 2,3-diphenyl-1*H*-indole-5-carboxylic acid (6)[7]

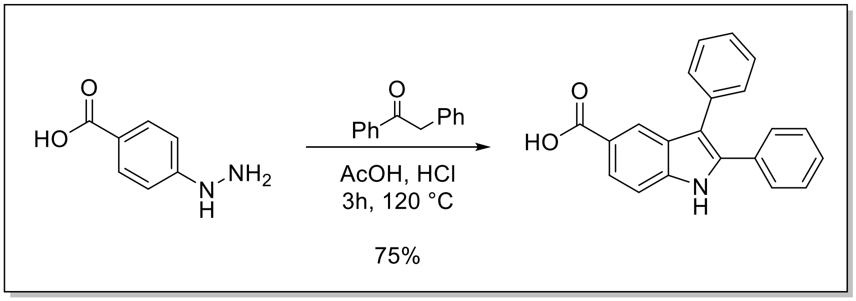

A mixture of 200 mL concentrated acetic acid and 60 mL concentrated hydrochloric acid is added to 4-hydrazino benzoic acid (3.00 g, 19.7 mmol, 1.0 eq.) and 2-phenylacetophenone (3.87 g, 19.7 mmol, 1.0 eq.). The mixture was refluxed (at 120 °C) for 3 hours (monitored by TLC, heptane:ethyl acetate 9:1, R_F = 0.21) and then cooled to room temperature. By slow addition of water (250 mL), the title compound precipitates out of solution. The pure product was obtained by filtration and thoroughly dried overnight in a vacuum oven to yield the title compound **6** as a brown powder (4.62 g – 75%). **^{1}H-NMR (500 MHz, DMSO-*d*$_6$):** δ (ppm) = 7.28-7.54 (m, 11H, Ar*H*), 7.80 (d, 1H, Ar*H*), 8.13 (s, 1H, HOOC-C-C*H*-C), 11.94 (s, 1H, N*H*), 12.48 (s, 1H, COO*H*). **^{13}C-NMR (125 MHz, DMSO-*d*$_6$):** *δ* (ppm) = 111.28 (CH), 114.41 (C), 121.28 (CH), 122.23 (C), 123.27 (CH), 126.53 (CH), 127.71 (C), 127.90 (CH), 128.18 (CH), 128.59 (CH), 128.81 (CH), 129.82 (CH), 131.88 (C), 134.61 (C), 135.54 (C), 138.54 (C), 168.23 (C). **LC-MS (m/z):** 312.0 $[M-H]^-$.

Synthesis of 2-phenyl-3-(2-phenylpropan-2-yl)-1*H*-indole (7)

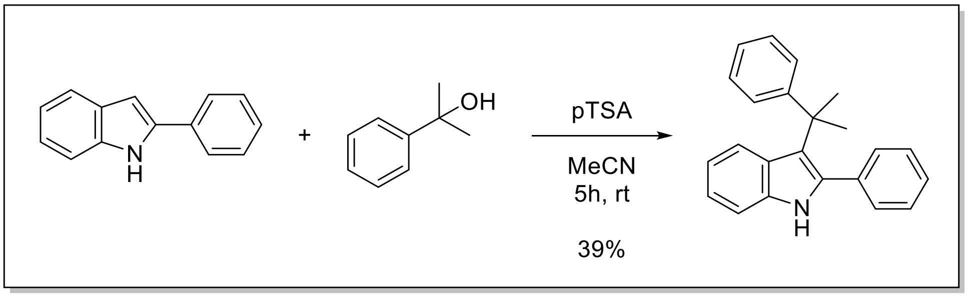

A solution of 2-phenyl-1*H*-indole (1.00 g, 5.17 mmol, 1.0 eq.), 2-phenyl-2-propanol (1.06 g, 7.76 mmol, 1.5 eq.) and *p*-toluenesulfonic acid monohydrate (0.20 g, 1.04 mmol, 0.2 eq.) in acetonitrile (20 mL) was stirred at room temperature under inert atmosphere. After five hours, the reaction went to completion (monitored via TLC, heptane:ethyl acetate 9:1, R_F = 0.26). The resulting mixture was filtered off and washed with acetonitrile (10 mL). The filtrate was diluted with ethyl acetate (80 mL) and washed with saturated aqueous sodium bicarbonate solution (2 x 10 mL). After drying over magnesium sulfate, the solvent was concentrated *in vacuo* to obtain a green oil. Finally, the 2-phenylpropene by-product was removed via column chromatography (silica, heptane:ethyl acetate 9:1, R_F = 0.26), giving indole **7** as a white solid (0.63 g – 39%). **^{1}H-NMR (400 MHz, DMSO-*d*$_6$):** *δ* (ppm) = 1.49 (s, 6H, C*H*$_3$), 6.69 (t, 1H, Ar*H*), 6.78 (d, 1H, Ar*H*), 6.95 (t, 1H, Ar*H*), 7.11 (m, 1H, Ar*H*), 7.23 (m, 3H, Ar*H*), 7.33 (m, 2H, Ar*H*), 7.41-7.48 (m, 3H, Ar*H*), 7.50-7.56 (m, 2H, Ar*H*), 10.96 (s, 1H, N*H*). **^{13}C-NMR (100 MHz, DMSO-*d*$_6$):** *δ* (ppm) = 32.04 (CH_3), 40.15 (C), 110.77 (CH), 117.95 (CH), 119.20 (C), 120.52 (CH), 120.86 (CH), 125.22 (CH), 125.97 (CH), 126.81 (C), 127.51 (CH), 127.89 (CH), 128.03 (CH), 130.66 (CH), 134.83 (C), 135.67 (C), 136.08 (C), 151.41 (C). **LC-MS (m/z):** 312.1 $[MH]^+$. **HRMS (m/z):** *calc.*: 312.1747, *found*: 312.1728 $[MH]^+$.

Synthesis of 3-(1,1-diphenylethyl)-2-phenyl-1*H*-indole

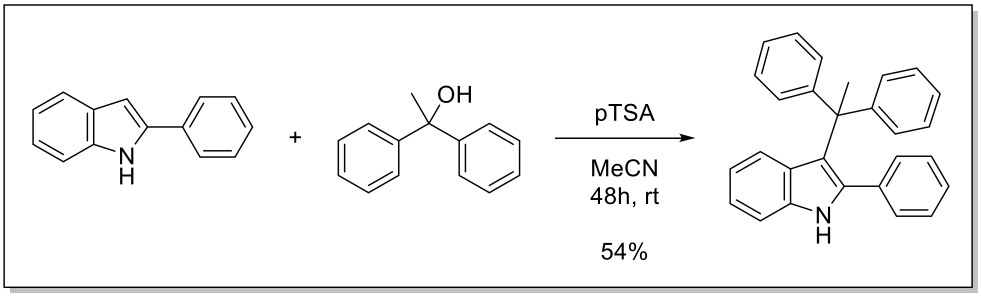

A solution of 2-phenyl-1*H*-indole (1.00 g, 5.17 mmol, 1.0 eq.), 1,1-diphenylethanol (1.54 g, 7.76 mmol, 1.5 eq.) and *p*-toluenesulfonic acid monohydrate (0.20 g, 1.04 mmol, 0.2 eq.) in acetonitrile (20 mL) was stirred at room temperature under inert atmosphere. After two days, the reaction went to completion (monitored via TLC, heptane:ethyl acetate 9:1, RF = 0.39). The resulting mixture was filtered off and washed with acetonitrile (10 mL). The filtrate was diluted with ethyl acetate (80 mL) and washed with saturated aqueous sodium bicarbonate solution (2 x 10 mL). After drying over magnesium sulfate, the solvent was concentrated *in vacuo*. Overnight drying of the residue in a vacuum oven at 40 °C gave 3-(1,1-diphenylethyl)-2-phenyl-1*H*-indole as a pale pink powder (1.05 g – 54%). **^{1}H-NMR (400 MHz, DMSO-*d$_6$*):** δ (ppm) = 1.87 (s, 3H, C*H*$_3$), 6.20 (d, 1H, Ar*H*), 6.61 (t, 1H, Ar*H*), 6.96 (t, 1H, Ar*H*), 7.06-7.31 (m, 16H, Ar*H*), 11.15 (s, 1H, N*H*). **^{13}C-NMR (100 MHz, DMSO-*d$_6$*):** δ (ppm) = 31.78 (CH_3), 48.33 (C), 110.94 (CH), 118.18 (CH), 118.85 (C), 120.48 (CH), 121.33 (CH), 125.71 (CH), 127.50 (CH), 127.68 (CH), 127.91 (CH), 128.16 (C), 129.58 (CH), 135.49 (C), 135.57 (C), 135.97 (C), 149.36 (C). **LC-MS (m/z):** 374.1 $[MH]^+$.

Synthesis of 2-phenyl-3-trityl-1*H*-indole

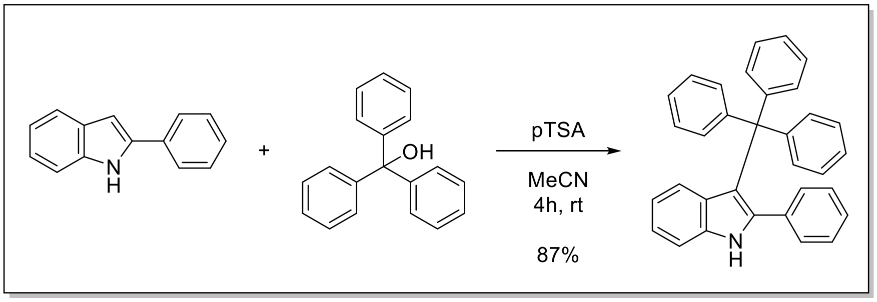

A solution of 2-phenyl-1*H*-indole (1.00 g, 5.17 mmol, 1.0 eq.), triphenylmethanol (2.02 g, 7.76 mmol, 1.5 eq.) and *p*-toluenesulfonic acid monohydrate (0.20 g, 1.04 mmol, 0.2 eq.) in acetonitrile (20 mL) was stirred at room temperature under inert atmosphere. After four hours, the reaction went to completion (monitored via TLC, heptane:ethyl acetate 9:1, R_F = 0.36). The resulting mixture was filtered off and washed with acetonitrile (10 mL). The filtrate was diluted with ethyl acetate (80 mL) and washed with saturated aqueous sodium bicarbonate solution (2 x 10 mL). After drying over magnesium sulfate, the solvent was concentrated *in vacuo* to obtain 2-phenyl-3-trityl-1*H*-indole as a white solid (1.98 g – 87%). **^{1}H-NMR (400 MHz, DMSO-*d$_6$*):** δ (ppm) = 6.51 (d, 1H, Ar*H*), 6.61 (t, 1H, Ar*H*), 6.92-7.14 (m, 21H, Ar*H*), 7.26 (d, 1H, Ar*H*), 11.09 (s, 1H, N*H*). **^{13}C-NMR (100 MHz, DMSO-*d$_6$*):** δ (ppm) = 59.79 (C), 110.77 (CH), 116.35 (C), 118.06 (CH), 120.74 (CH), 122.51 (CH), 125.55 (CH), 126.31 (CH), 126.82 (CH), 127.01 (CH), 127.49 (CH), 127.75 (CH), 128.17 (C), 129.26 (CH), 130.75 (CH), 135.22 (C), 135.34 (C), 136.93 (C), 145.99 (C). **LC-MS (m/z):** 436.1 $[MH]^+$.

Synthesis of methyl 2,3-diphenyl-1*H*-indole-5-carboxylate (8)

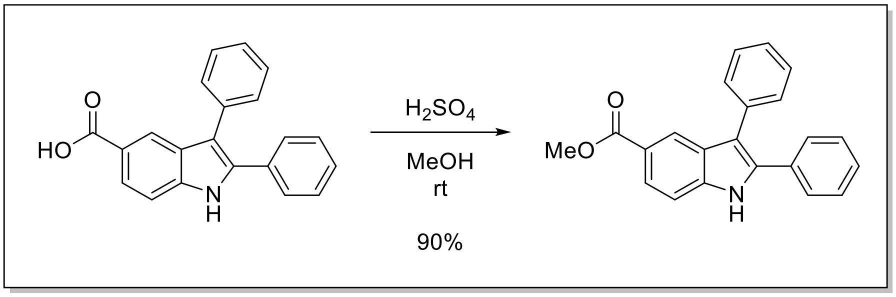

A mixture of 2,3-diphenyl-1*H*-indole-5-carboxylic acid (**6**) (0.300 g, 1.19 mmol) and 1 mL concentrated sulfuric acid in 10 mL methanol is stirred at room temperature until no starting component could be detected on TLC (heptane:ethyl acetate 9:1, R_F = 0.48). After extraction with 40 mL of ethyl acetate, the solution is washed with saturated aqueous sodium bicarbonate solution (2 x 10 mL) and dried over magnesium sulfate. Solvent removal *in vacuo* gave the desired title compound **8** (0.283 g – 90%). **^{1}H-NMR (400 MHz, DMSO-*d₆*):** δ (ppm) = 3.82 (s, 3H, CO-O-C*H*$_3$), 7.32-7.41 (m, 6H, Ar*H*), 7.41-7.50 (m, 4H, Ar*H*), 7.53 (d, 1H, Ar*H*), 7.81 (dd, 1H, Ar*H*), 8.13 (s, 1H, CO-C-C*H*-C), 11.99 (s, 1H, N*H*). **^{13}C-NMR (100 MHz, DMSO-d_6):** δ (ppm) = 51.71 (CH_3), 111.50 (CH), 114.46 (C), 121.05 (CH), 121.12 (C), 122.97 (CH), 126.62 (CH), 127.72 (C), 127.97 (CH), 128.16 (CH), 128.59 (CH), 128.84 (CH), 129.81 (CH), 131.73 (C), 134.44 (C), 135.74 (C), 138.61 (C), 167.06 (C). **LC-MS (m/z):** 328.1 $[M-H]^-$.

Synthesis of 4-(4-azobenzene)-1,2,4-triazoline-3,5-dione (TAD-dye) (12)

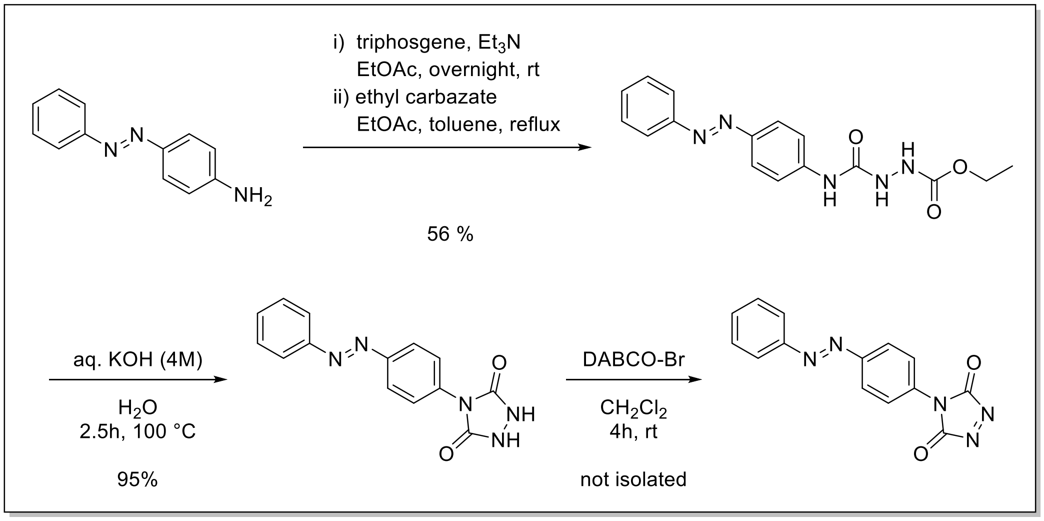

Triphosgene (5.70 g, 19.2 mmol, 0.35 eq) was dissolved in 100 mL ethyl acetate and cooled in an ice-bath. To this, a mixture of 4-aminoazobenzene (10.82 g, 55.0 mmol, 1.00 eq) and dry triethylamine (16.8 mL, 0.121 mol, 2.20 eq) in 40 ml ethyl acetate was slowly added. The ice-bath was removed and the solution was stirred overnight under inert atmosphere. The resulting reaction mixture was filtered directly into a mixture of ethyl carbazate (6.29 g, 60.4 mmol, 1.10 eq) in 20 mL ethyl acetate and the residue was washed with another 100 mL ethyl acetate. A precipitate is rapidly formed in the clear filtrate. After stirring for 5 minutes, the reaction mixture was evaporated *in vacuo*. To this crude, 200 mL toluene and 100mL ethyl acetate were added and the resulting mixture was heated to reflux. Another 100mL toluene was added, after which the mixture was cooled to room temperature and filtered. The residue was dried overnight in a vacuum oven to obtain 4-(4-azobenzene)-1-(ethoxycar-bonyl) semicarbazide as an orange powder (10.2 g – 56%). **^{1}H-NMR (400 MHz, DMSO-d_6)**: δ (ppm) = 1.21 (t, 3H, C*H*$_3$), 4.08 (q, 2H, C*H*$_2$), 7.48-7.61 (m, 3H, Ar*H*), 7.64-7.78 (m, 2H, Ar*H*), 7.81-7.89 (m, 4H, Ar*H*), 8.21 (s, 1H, Ar-N*H*), 8.63 + 9.01 (s, 1H, Ar-NH-C(O)-N*H*), 9.20 (s, 1H, Ar-NH-C(O)-NH-N*H*). **^{13}C-NMR (100 MHz, DMSO-d_6):** δ (ppm) = 14.54 (CH_3),

60.59 (CH_2), 122.22 (CH), 123.72 (CH), 129.37 (CH), 130.78 (CH), 143.21 (C), 146.66 (C), 152.08 (C). **LC-MS (m/z):** 328.20 $[MH]^+$. **HRMS (m/z):** *calc.*: 328.1404, *found*: 328.1395 $[MH]^+$. Next, 4-(4-azobenzene)-1-(ethoxycarbonyl) semicarbazide (1.00 g, 3.05 mmol, 1.0 eq.) in 2.5 mL of 4 M aqueous potassium hydroxide solution was placed under inert atmosphere and refluxed for 2.5h at 100 °C. the resulting dark orange-brown solution was cooled to room temperature and acidified to pH = 1 with 1 M aqueous hydrochloric acid solution. The obtained precipitate was filtered off, washed with water (3 x 5 mL) and dried in a vacuum oven overnight at 40 °C to yield 4-(4-azobenzene) urazole (**11**) as an orange powder (0.817 g – 95%). **^{1}H-NMR (400 MHz, DMSO-*d*$_6$)**: δ (ppm) = 7.55-7.66 (m, 3H, Ar*H*), 7.76 (dt, 2H, Ar*H*), 7.92 (m, 2H, Ar*H*), 8.00 (dt, 2H, Ar*H*), 10.65 (s, 2H, N*H*). **^{13}C-NMR (100 MHz, DMSO-*d*$_6$):** δ (ppm) = 122.64 (CH), 122.89 (CH), 126.23 (CH), 129.51 (CH), 131.73 (CH), 134.63 (C), 150.34 (C), 151.88 (C), 152.85 (C). **LC-MS (m/z):** 282.10 $[MH]^+$. **HRMS (m/z):** *calc.*: 282.0986, *found*: 282.0994 $[MH]^+$. Finally, a mixture of 4-(4-azobenzene) urazole (**11**, 500 mg, 1.78 mmol, 1.0 eq.) and DABCO-Br (559 mg, 0.356 mmol, 0.2 eq.) in 25 mL dry dichloromethane was placed under inert atmosphere and stirred at room temperature for 4h. The resulting dark red mixture was taken up with a syringe to remove the heterogenous oxidant with a syringe filter. The obtained dark red solution of 4-(4-azobenzene)-1,2,4-triazoline-3,5-dione (**12**) was used without isolation.

Synthesis of 2,3-diphenyl-1*H*-indole blocked TAD-dye (13)

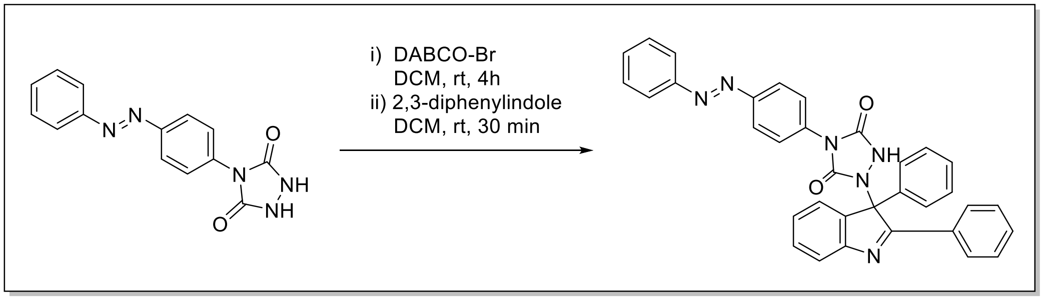

A mixture of 4-(4-azobenzene) urazole (**11**, 500 mg, 1.78 mmol, 1.0 eq.) and DABCO-Br (559 mg, 0.356 mmol, 0.2 eq.) in 25 mL dry dichloromethane was placed under inert atmosphere and stirred at room temperature for 4h. The resulting dark red mixture was taken up with a syringe and added to a solution of 2,3-diphenylindole (**5**, 479 mg, 1.78 mmol, 1.0 eq.) in 5 mL dry dichloromethane through a syringe filter. The syringe and filter were washed with an additional 5 mL dry dichloromethane. The resulting mixture was stirred under inert atmosphere at room temperature for 30 minutes, during which the red color completely disappeared. Solvent removal *in vacuo* (< 30 °C) gave a bright orange residue. Purification via column chromatography (silica, hexane:ethyl acetate 7:3 with a gradient to 1:1, R_F (hexane: ethyl acetate 1:1) = 0.25) – to remove unreacted indole – yielded 2,3-diphenylindole blocked TAD-dye **13** as a bright orange powder (859 mg – 88%). Obtained as an inseparable diastereomeric mixture (*de* = 77 %). **^{1}H-NMR (500 MHz, DMSO-*d*$_6$)**: Major diastereomer: δ (ppm) = 7.21-7.48 (m, 9H, Ar*H*), 7.49-7.64 (m, 7H, Ar*H*), 7.70 (d, 1H, Ar*H*), 7.87-7.92 (m, 2H, Ar*H*), 7.94 (d, 2H, Ar*H*), 8.16 (d, 2H, Ar*H*), 11.01 (s, 1H, N*H*); Minor diastereomer, some resolved resonances: δ (ppm) = 6.83 (d, 2H, Ar*H*), 6.90 (d, 2H, Ar*H*), 7.68 (d, 1H, Ar*H*), 8.10 (d, 2H, Ar*H*). **^{13}C-NMR (125 MHz, DMSO-*d*$_6$):** δ (ppm) = 80.91 (C), 121.11 (CH), 122.65 (CH), 122.98 (CH), 123.49 (CH), 126.25 (CH), 126.93 (CH), 127.16 (CH), 128.13 (CH), 128.41 (CH), 128.77 (CH), 128.85 (CH), 129.51 (CH), 130.43 (CH), 130.85 (CH), 131.84 (CH), 132.19 (C), 133.52 (C), 134.80 (C), 150.63 (C), 151.81 (C), 154.52 (C), 176.11 (C). **LC-MS (m/z):** 549.20 $[MH]^+$. **HRMS (m/z):** *calc.*: 549.2034 $[MH]^+$, *found*: 270.1267 $[\mathbf{5}H]^+$ (fragment ion).

Synthesis of 3-methyl-2-phenyl-1*H*-indole blocked TAD-dye (14)

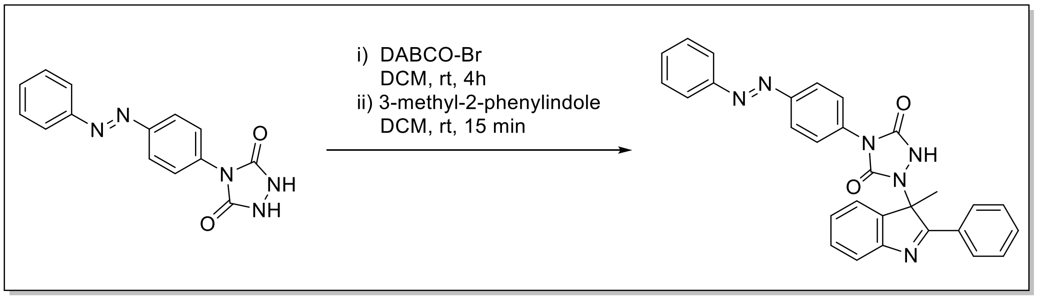

A mixture of 4-(4-azobenzene) urazole (**11**, 250 mg, 0.90 mmol, 1.0 eq.) and DABCO-Br (280 mg, 0.18 mmol, 0.2 eq.) in 10 mL dry dichloromethane was placed under inert atmosphere and stirred at room temperature for 4h. The resulting dark red mixture was taken up with a syringe and added to a solution of 3-methyl-2-phenylindole (**3**, 184 mg, 0.90 mmol, 1.0 eq.) in 3 mL dry dichloromethane through a syringe filter. The syringe and filter were washed with an additional 2 mL dry dichloromethane. The resulting mixture was stirred under inert atmosphere at room temperature for 15 minutes, during which the red color completely disappeared. Solvent removal *in vacuo* (< 30 °C) gave a bright orange residue. Purification via column chromatography (silica, hexane:ethyl acetate 1:1, R_F (hexane:ethyl acetate 1:1) = 0.29) – to remove unreacted indole – yielded 3-methyl-2-phenylindole blocked TAD-dye **14** as a bright orange powder (305 mg – 71%). Obtained as an inseparable diastereomeric mixture (*de* = 80 %). **^{1}H-NMR (400 MHz, DMSO-*d*$_6$)**: Major diastereomer: δ (ppm) = 1.82 (s, 3H, CH_3), 7.31 (t, 1H, Ar*H*), 7.44 (t, 1H, Ar*H*), 7.50-7.64 (m, 10H, Ar*H*), 7.85-7.94 (m, 4H, Ar*H*), 8.24-8.30 (m, 2H, Ar*H*), 11.34 (s, 1H, N*H*); Minor diastereomer, some resolved resonances: δ (ppm) = 1.77 (s, 3H, CH_3), 6.81 (m, 2H, Ar*H*), 6.87 (m, 2H, Ar*H*), 7.14 (m, 1H, Ar*H*), 8.19-8.24 (m, 2H, Ar*H*). **^{13}C-NMR (100 MHz, DMSO-*d*$_6$):** δ (ppm) = 22.96 (CH_3), 72.28 (C), 119.77 (CH), 120.40 (CH), 120.97 (CH), 121.27 (CH), 122.64 (CH), 122.93 (CH), 125.84 (CH), 126.03 (CH), 126.78 (CH), 128.02 (CH), 128.79 (CH), 128.94 (CH), 129.49 (CH), 129.70 (CH), 131.31 (CH), 131.47 (C), 131.82 (CH), 133.58 (C), 139.50 (C), 150.51 (C), 151.80 (C), 152.35 (C), 152.75 (C), 152.96 (C), 175.92 (C). **LC-MS (m/z):** 487.20 $[MH]^+$. **HRMS (m/z):** *calc.*: 487.1877, *found*: 487.1862 $[MH]^+$.

Synthesis of trivalent alkene

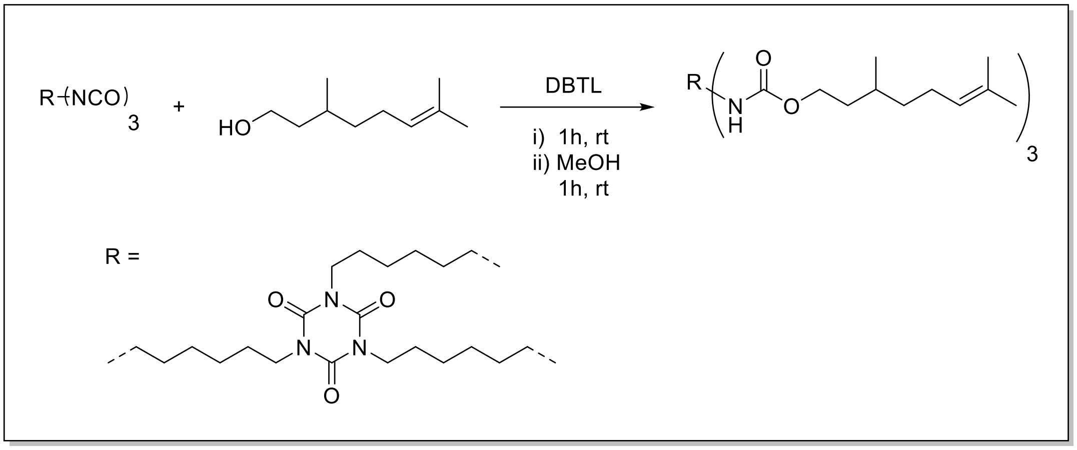

Desmodur XP 2489 polyisocyanate (5.20 g, 26.0 mmol NCO, 1.0 eq.) and citronellol (4.07 g, 26.0 mmol, 1.0 eq.) in ethyl acetate (5 mL) were mixed thoroughly until a homogeneous solution was obtained, after which dibutyltin dilaurate (DBTL, 100 µL) was added. The resulting solution was mixed for 1 hour. After this, methanol (5 mL) was added and stirred for an additional hour. The solvent was removed *in vacuo* followed by drying overnight in a vacuum oven at 40 °C, to yield the multivalent citronellol derivative as a viscous liquid (alkene equivalent: 2.8 mmol g^{-1}). **^{1}H-NMR (300 MHz, $CDCl_3$):** δ (ppm) = 0.92 (d, 9H, CH-CH_3), 1.19 (m, 3H, CH_3-C*H*), 1.28–1.72 (m, 54H, N-CH_2-(CH_2)$_4$-CH_2-NH + O-CH_2-CH_2 + CH_2-CH_2-CH + C-(CH_3)$_2$), 1.98 (m, 6H, CH_2-CH=C), 3.16 (q, 6H, NH-CH_2), 3.86 (t, 6H, N-CH_2), 4.09 (m, 6H, O-CH_2), 4.69 (s, 3H, N*H*), 5.09 (t, 3H, C*H*=C).

Synthesis of a TAD-reactive network by crosslinking of the trivalent alkene with MDI-derived bisTAD

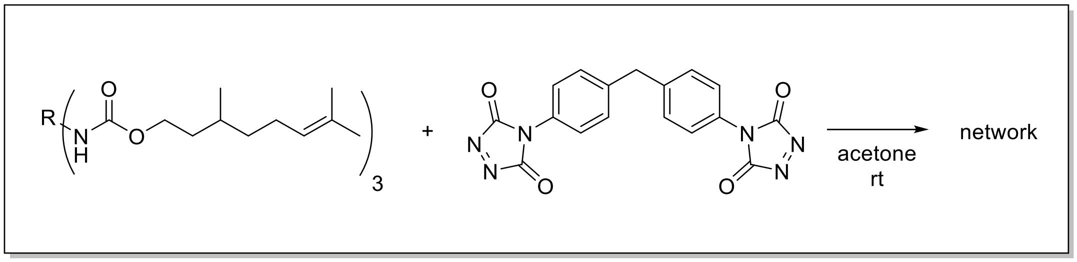

To a solution of the multivalent citronellol derivative (*vide supra*) (1.00 g, 2.80 mmol alkene, 1.0 eq.) in 3 mL acetone was added a solution of MDI-derived bisTAD (0.41 g, 1.12 mmol, 0.4 eq.) in 3 mL acetone. Once added, the mixture was quickly shaken and poured out into a petri dish. Gelation occurred within 5 seconds and complete curing was observed by the complete disappearance of the red color. In the course of one minute after mixing, a yellowish-white precipitate was formed. After drying to the air overnight at room temperature, an almost transparent material – containing an excess of TAD-reactive moieties – was obtained.

Synthesis of 2,3-diphenylindole end-functionalized PEG (15)

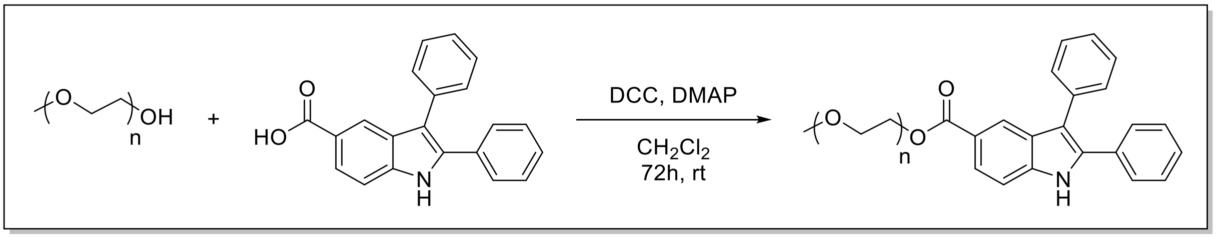

A mixture of 2,3-diphenyl-1*H*-indole-5-carboxylic acid (**6**, 0.940 g, 3.00 mmol, 6.0 eq.), PEG monomethyl ether 2000 (1.00 g, 0.50 mmol, 1.0 eq.) and 4-(dimethylamino)pyridine (DMAP, 0.061 g, 0.50 mmol, 1.0 eq.) in 5 mL anhydrous dichloromethane was placed under inert atmosphere and stirred at room temperature. To this, a solution of *N*,*N*'-dicyclohexylcarbodiimide (DCC, 0.516 g, 2.50 mmol, 5.0 eq.) in 5 mL anhydrous dichloromethane was added dropwise. After stirring for 72 hours at room temperature, the reaction mixture was filtered off directly into a 10-fold excess of cold diethyl ether. The resulting yellowish-brown precipitate was filtered off and washed with cold diethyl ether. The residue was then redissolved in a minimal amount of dichloromethane, precipitated a second time in cold diethyl ether (10-fold excess), filtered off and dried in a vacuum oven at 30 °C overnight to yield PEG-supported 2,3-diphenylindole (**15**) as a brown solid (0.896 g – 78 %). **^{1}H-NMR (400 MHz, DMSO-*d*$_6$)**: δ (ppm) = 3.24 (s, 3H, C*H*$_3$), 3.40-3.69 (m, 176 H, O-C*H*$_2$), 3.74 (t, 2H, C(O)-O-CH$_2$-C*H*$_2$), 4.37 (t, 2H, C(O)-O-C*H*$_2$), 7.28-7.40 (m, 5H, Ar*H*), 7.40-7.51 (m, 5H, Ar*H*), 7.54 (d, 1H, Ar*H*), 7.82 (dd, 1H, Ar*H*), 8.16 (s, 1H, O-C(O)-C*H*), 11.99 (s, 1H, N*H*).

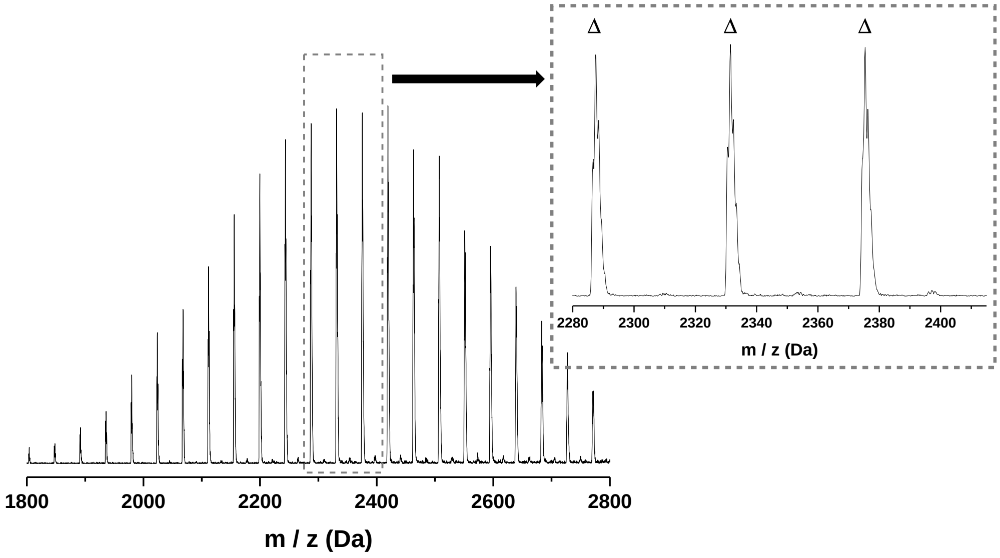

Figure S15. MALDI-TOF analysis of PEGylated 2,3-diphenylindole 15 (Δ).

Table S1. Theoretical and experimental masses of PEGylated 2,3-diphenylindole 15.

$\Delta = [15+Na]^+$	
**Theoretical m/z (Da)**	**Experimental m/z (Da)**
2287.2685	2287.4503
2331.2947	2331.4248
2375.3209	2375.3720

Synthesis of PEG-supported blocked TAD-dye (16)

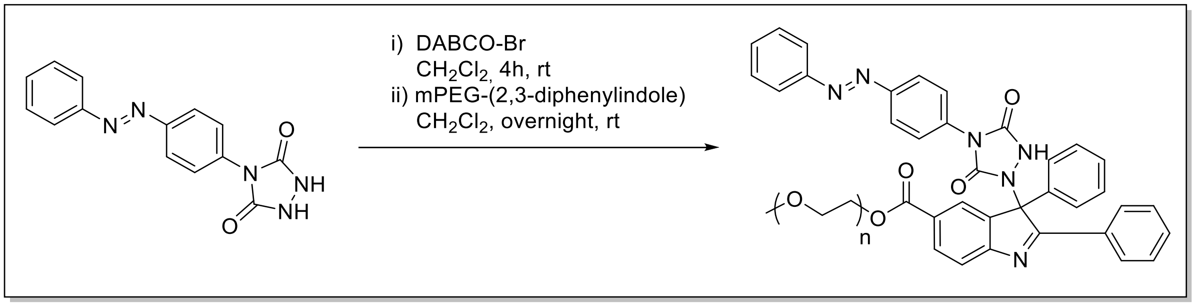

A mixture of 4-(4-azobenzene) urazole (**11**, 214 mg, 0.76 mmol, 3.0 eq.) and DABCO-Br (239 mg, 0.15 mmol, 0.2 eq.) in 10 mL dry dichloromethane was placed under inert atmosphere and stirred at room temperature for 4h. The resulting dark red mixture was taken up with a syringe and added to a solution of mPEG-(2,3-diphenylindole) **15** (574 mg, 0.25 mmol, 1.0 eq.) in 3 mL dry dichloromethane through a syringe filter. The syringe and filter were washed with an additional 2 mL dry dichloromethane. The resulting mixture was stirred overnight under inert atmosphere at room temperature. The orange-red mixture was then precipitated into cold diethyl ether (150 mL) and was filtered off. The resulting orange residue was redissolved in a minimal amount of tetrahydrofuran and purified by means of column chromatography (SEC Bio-Beads S-X1 Support, tetrahydrofuran, R_F (silica, heptane:ethyl acetate 1:1) = 0.00). The fractions containing polymeric material were concentrated *in vacuo* (< 30 °C) followed by precipitation in cold diethyl ether (10-fold excess), filtration and overnight drying to the air at room temperature to give PEG-supported blocked TAD-dye (**16**) as a dark orange residue (445 mg – 68%). **^{1}H-NMR (400 MHz, DMSO-*d*$_6$)**: δ (ppm) = 3.24 (s, 3H, C*H*$_3$), 3.41-3.70 (m, 176 H, O-C*H*$_2$), 3.74 (t, 2H, C(O)-O-CH$_2$-C*H*$_2$), 4.39 (t, 2H, C(O)-O-C*H*$_2$), 7.28 (m, 5H, Ar*H*), 7.40-7.49 (m, 3H, Ar*H*), 7.53 (d, 2H, Ar*H*), 7.57-7.64 (m, 3H, Ar*H*), 7.84 (dd, 1H, Ar*H*), 7.89 (m, 2H, Ar*H*), 7.94 (dt, 2H, Ar*H*), 8.11-8.23 (m, 4H, Ar*H*), 11.11 (s, 1H, N*H*).

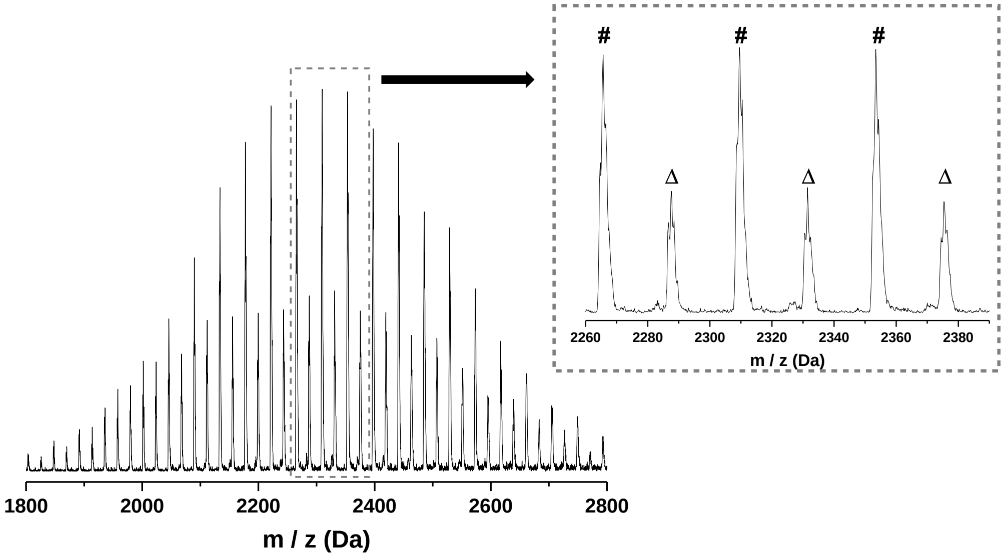

Figure S16. MALDI-TOF analysis after reaction with the TAD-dye gives a bimodal distribution, which can be assigned to the TAD-indole dye-PEG conjugate 16 after fragmentation (#) and the initial – unreacted or fragmented – PEGylated indole substrate 15 (Δ).

Table S2. Theoretical and experimental masses of dye-PEG conjugate 16.

# = $[15+H]^+$		Δ = $[15+Na]^+$	
**Theoretical m/z (Da)**	**Experimental m/z (Da)**	**Theoretical m/z (Da)**	**Experimental m/z (Da)**
2265.2860	2265.6200	2287.2685	2287.5962
2309.3122	2309.5318	2331.2947	2331.4248
2353.3348	2353.4211	2375.3209	2375.3720

COMPUTATIONAL METHODS AND THEORETICAL DATA

COMPUTATIONAL METHODOLOGY

The M06-2X/6-31+G(d,p) level of theory,[8,9] which has been shown successful to describe thermochemistry, was used for geometry optimizations. A thorough conformational analysis was performed on all reactants, transition states, intermediates and products to identify the most plausible conformers. The stationary points were characterized as minima (ground states) or first order saddle points (transition states) by normal modes analysis. IRC (intrinsic reaction coordinate) calculations[10-12] followed by geometry optimizations were used to verify the corresponding reactant and product complexes. Since the reactions under study take place in CH_2Cl_2 or DMSO, which cannot form hydrogen bonds with the reactive substrate, the solvent environment was taken into account by means of a continuum model.[13-16] All computations were carried out with the Gaussian 09 program package.[17]

CARTESIAN COORDINATES OF TRANSITION STATES

Cartesian coordinates, energy, imaginary and low frequencies of the optimized geometry [PCM (ε = 8.93) M06-2X/6-31+G(d,p)] of:

TS-1-MeTAD

C	-1.830380	0.023816	2.788336
C	-0.902476	0.335269	1.808438
C	-0.379582	-0.608667	0.915406
C	-0.789647	-1.937610	0.995744
C	-1.729554	-2.273630	1.975261
C	-2.241000	-1.311155	2.854308
N	-0.333166	1.573422	1.469203
C	0.459385	1.481701	0.409557
C	0.441950	0.101723	-0.068817
C	1.633469	-0.578893	-0.691050
C	1.179410	2.685746	-0.147560
C	0.972305	2.777619	-1.669621
C	0.651850	3.974911	0.496884
C	2.685197	2.550504	0.167904
N	-0.731174	0.139208	-1.603181
N	-0.992394	-1.113639	-1.962024
C	-2.295425	-1.417804	-1.618635
N	-2.884083	-0.255275	-1.079108
C	-1.947818	0.722355	-1.049563
O	-2.076866	1.874738	-0.673450
C	-4.232809	-0.138188	-0.570106
O	-2.902127	-2.459068	-1.823225
H	-4.737326	0.706578	-1.042877
H	-2.224512	0.774624	3.464365
H	-2.968151	-1.604922	3.603739
H	-2.069847	-3.300585	2.057954
H	-0.391322	-2.688104	0.320223
H	2.091688	0.071948	-1.442947
C	2.672518	-1.005303	0.355364
H	1.251741	-1.455889	-1.224548
H	3.191871	3.460197	-0.168581
H	2.857564	2.438205	1.242943
H	3.137191	1.701574	-0.350643
H	1.539907	3.637279	-2.038717
H	1.318516	1.883296	-2.189899
H	-0.084976	2.917143	-1.905041
H	1.156401	4.829633	0.039430
H	-0.423668	4.090600	0.326458
H	0.860850	4.016384	1.571871
H	-4.217262	0.005656	0.513655
H	-4.755059	-1.063911	-0.811921
H	-0.586732	2.449020	1.912222
C	3.999271	-1.467786	-0.257357
H	2.871350	-0.173232	1.043933
H	2.257496	-1.818437	0.967051
C	4.996732	-1.814611	0.848792
C	3.807018	-2.657953	-1.199833
H	4.409754	-0.629446	-0.840515
H	4.770990	-3.009929	-1.580314
H	3.330775	-3.490564	-0.667161
H	3.181294	-2.405934	-2.060718
H	5.965706	-2.102795	0.429597
H	5.155448	-0.966207	1.522468
H	4.625483	-2.655815	1.446438

Energy (m06-2X/6-31++G**) = -1148.006446 Hartree

Frequencies(cm-1): -421.6945, 33.9760, 46.7235,...

TS-2-MeTAD

C	-2.838762	-2.092119	1.254815
C	-1.987494	-1.551858	0.278032
C	-2.288799	-1.716600	-1.082718
C	-3.425988	-2.425732	-1.455644
C	-4.272146	-2.959115	-0.482497
C	-3.978633	-2.790015	0.871942
C	-0.782317	-0.831562	0.664868
N	-0.075705	-1.151522	1.746595
C	1.033965	-0.305927	1.896118
C	0.975935	0.634414	0.856551
C	-0.141371	0.278323	-0.023290
C	1.967114	1.606905	0.744309
C	3.005767	1.592457	1.679403
C	3.048373	0.638276	2.704199
C	2.053511	-0.334323	2.835240
C	-0.945768	1.345472	-0.721636
N	0.736330	-0.507011	-1.532265
N	1.502593	0.418678	-2.098966
C	2.827149	0.133483	-1.824119
N	2.871220	-1.077304	-1.102981
C	1.601569	-1.502094	-0.901954
O	3.817349	0.740937	-2.202359
C	4.060306	-1.723723	-0.592218
O	1.224611	-2.515712	-0.343497
H	4.095468	-2.761131	-0.930041
H	2.082102	-1.072809	3.629228
H	3.867478	0.655283	3.415188
H	3.793729	2.335390	1.613808
H	1.932878	2.351694	-0.044662
H	-1.614892	0.888705	-1.458106
C	-1.755275	2.189749	0.268356
H	-0.233969	1.965471	-1.275392
H	4.065823	-1.694739	0.500642
H	4.920752	-1.178685	-0.980172
H	-0.230072	-1.991602	2.293482
H	-1.607931	-1.322496	-1.830318
H	-3.650777	-2.564871	-2.507800
H	-5.162545	-3.504272	-0.778582
H	-4.641888	-3.195246	1.628648
H	-2.629674	-1.935776	2.309427
C	-2.615128	3.269864	-0.399100
H	-2.410383	1.528234	0.852378
H	-1.072156	2.669657	0.984308
C	-3.462287	3.985959	0.654149
C	-1.765886	4.278837	-1.175570
H	-3.293505	2.770842	-1.106284
H	-2.392001	5.080379	-1.579907
H	-1.019965	4.736295	-0.513423
H	-1.236472	3.817665	-2.013898
H	-4.116762	4.732027	0.192970
H	-4.088195	3.280098	1.209578
H	-2.816812	4.504177	1.373678

Energy (m06-2X/6-31++G**) = -1221.7909037 Hartree

Frequencies(cm-1): -439.1547, 22.4546, 27.2255,...

TS-3-MeTAD

C	0.065706	-0.186054	0.015834
C	0.050149	-0.112577	1.400019
C	1.211505	0.027697	2.174286
C	2.456430	0.086535	1.551645
C	2.492895	0.018885	0.155985
C	1.319137	-0.113410	-0.597214
N	-1.036110	-0.127596	2.286966
C	-0.635226	0.042850	3.544743
C	0.809459	0.216957	3.571514
C	1.655122	-0.315542	4.694673
C	-1.570528	0.087829	4.660066
N	1.069501	2.093598	3.843313
N	2.363990	2.359214	3.700781
C	2.555118	3.025631	2.504930
N	1.290745	3.239575	1.919051
C	0.350018	2.675687	2.712998
O	-0.857729	2.666793	2.561965
C	1.037504	3.880532	0.647186
O	3.602574	3.458234	2.048303
H	0.288592	4.665086	0.769536
H	-0.845898	-0.290291	-0.562563
H	1.382483	-0.164901	-1.678902
H	3.448339	0.067053	-0.355793
H	3.368225	0.184289	2.131893
H	1.238777	-0.063142	5.670607
H	1.734739	-1.403439	4.612778
H	2.655426	0.117745	4.615700
H	0.683290	3.147563	-0.082632
H	1.977423	4.315053	0.306914
H	-2.007314	-0.111780	1.995687
C	-1.291457	0.904363	5.767041
C	-2.199232	0.973874	6.818837
C	-3.377038	0.226465	6.781473
C	-3.655032	-0.590224	5.683681
C	-2.758860	-0.658696	4.623367
H	-0.387808	1.505130	5.771285
H	-1.988583	1.615581	7.667790
H	-4.077932	0.277972	7.608358
H	-4.566079	-1.178485	5.657822
H	-2.966486	-1.316560	3.784386

Energy (m06-2X/6-31++G**) = -1064.6060649 Hartree

Frequencies(cm-1): -462.1695, 41.4965, 52.6824,...

TS-4-MeTAD

C	0.112884	0.501719	0.523242	C	-0.153888	3.017081	2.514914
C	-0.097189	-0.057143	1.794269	N	-1.343380	2.770421	1.869772
C	1.017120	-0.310864	2.623623	O	0.809465	3.653994	2.107929
C	2.297127	-0.012230	2.188631	C	0.808052	2.546526	4.808612
C	2.493360	0.541797	0.917444	H	-0.983923	-1.722856	3.750613
C	1.404887	0.788816	0.088005	H	-3.147071	-2.549764	5.305143
C	-1.434723	-0.278100	2.304658	H	-5.580832	-2.072942	5.644483
C	-2.659590	0.476930	2.013307	H	-5.525770	0.894625	2.535912
C	-3.654893	-0.115975	2.932023	H	-2.467618	1.619781	0.190653
C	-3.013292	-1.078503	3.718579	H	-3.199327	-0.000293	-0.005762
N	-1.681177	-1.130597	3.317627	H	-4.122862	1.326408	0.726261
C	-5.002889	0.146380	3.121577	H	-0.723012	0.685200	-0.136964
C	-5.677761	-0.574367	4.112533	H	1.553970	1.218856	-0.896205
C	-5.014198	-1.538927	4.889388	H	3.497047	0.782541	0.582743
C	-3.664692	-1.810456	4.704636	H	3.144047	-0.190512	2.842653
C	-3.129840	0.880467	0.638122	H	0.878684	-0.689153	3.632714
C	-7.121304	-0.346234	4.380858	H	-8.599615	0.683894	3.826065
O	-7.764278	-0.934543	5.225084	H	1.112379	3.582986	4.962688
O	-7.663776	0.589886	3.586906	H	0.416165	2.131958	5.737808
N	-2.135525	2.089042	2.703279	H	1.671090	1.962744	4.476780
C	-1.441972	1.864950	3.958433	O	-1.886861	1.279445	4.929557
N	-0.242689	2.487518	3.817444				

Energy (m06-2X/6-31++G**) = -1253.1330364 Hartree

Frequencies(cm-1): -439.4892, 35.9543, 47.2502,...

TS-5-MeTAD

C	-0.023878	-0.361336	0.102669	N	-4.353390	1.508156	1.811162
C	-0.042126	-0.118374	1.478119	C	-3.886425	0.235273	1.922040
C	1.152048	0.201262	2.134941	C	-5.541984	2.036491	2.443728
C	2.345382	0.296449	1.423076	H	-2.642792	-0.036132	5.234408
C	2.357829	0.061494	0.048102	H	-2.999831	-2.778578	5.640114
C	1.172496	-0.270304	-0.607075	H	-2.672172	-4.956524	4.479558
C	-1.277841	-0.333746	2.296338	H	-1.650632	-5.058931	2.235602
C	-1.565979	-1.716343	2.707623	H	-0.918753	-2.976540	1.077548
C	-2.141240	-1.679381	3.981293	H	-2.967478	1.966306	5.315440
N	-2.194719	-0.345746	4.383398	H	-3.096762	4.397370	5.415174
C	-1.661239	0.470591	3.447481	H	-1.915739	5.795453	3.730100
C	-2.551184	-2.825048	4.653818	H	-0.583447	4.707916	1.940476
C	-2.364444	-4.036684	3.993068	H	-0.416551	2.261818	1.831050
C	-1.783361	-4.095423	2.715823	H	1.144225	0.378743	3.207698
C	-1.373365	-2.935588	2.062864	H	3.263120	0.551368	1.943289
C	-1.666445	1.914555	3.564792	H	3.286383	0.135712	-0.508881
C	-2.420416	2.547709	4.579760	H	1.175145	-0.454490	-1.676676
C	-2.505276	3.927116	4.636814	H	-0.949266	-0.603512	-0.408437
C	-1.841898	4.713810	3.684207	H	-6.178736	1.197896	2.726633
C	-1.094090	4.106423	2.684544	H	-5.274030	2.614016	3.333169
C	-1.005222	2.715513	2.615821	H	-6.063582	2.682104	1.736382
N	-2.665888	0.197767	1.115374	O	-3.686614	3.384321	0.622198
N	-2.472394	1.380095	0.567190	O	-4.384848	-0.704867	2.505541
C	-3.491298	2.234588	0.978445				

Energy (m06-2X/6-31++G**) = -1256.276726 Hartree

Frequencies(cm-1): -391.7794, 36.5124, 49.0666,...

TS-6-MeTAD

C	0.049629	0.019395	0.028403	C	2.635605	3.916854	-0.499759
N	0.052262	0.058768	1.491457	C	3.532950	3.804206	0.572515
N	1.294996	0.114764	1.924366	C	3.097726	3.302480	1.791355
C	2.147649	0.132214	0.823168	C	1.772257	2.898557	1.954817
N	1.358866	0.061720	-0.334267	H	-1.316025	3.226402	-0.778471
O	3.366061	0.115041	0.827021	H	-3.961117	2.467430	-1.248587
C	1.867103	0.050302	-1.688794	H	-5.764729	1.046933	-0.259757
O	-0.930783	-0.087461	-0.678934	C	-5.721740	-0.386843	1.953387
C	-0.988245	1.539291	1.990378	H	-3.223692	-0.043835	3.037040
C	-2.370604	1.276268	1.555255	H	0.657145	3.587517	-1.205200
C	-2.594785	1.977806	0.364167	H	2.975608	4.297709	-1.456909
N	-1.437159	2.680704	0.063657	H	4.567158	4.107009	0.445272
C	-0.483037	2.487308	1.008806	H	3.788413	3.205292	2.621702
C	-3.804344	1.923733	-0.323949	H	1.451823	2.511384	2.911686
C	-4.800226	1.131780	0.229238	H	-0.931644	3.777991	3.474131
C	-4.595537	0.427306	1.430360	H	-0.764605	3.974668	5.934795
C	-3.376538	0.497491	2.109162	H	-0.514314	1.932633	7.331936
C	-0.759469	1.629555	3.468529	H	-0.435513	-0.299863	6.246066
C	-0.629307	0.483344	4.255919	H	-0.578266	-0.489359	3.778639
C	-0.543069	0.595717	5.642579	H	2.744555	-0.595596	-1.733661
C	-0.586966	1.849023	6.252308	H	1.083989	-0.334150	-2.342674
C	-0.725842	2.995081	5.469395	H	2.144417	1.061894	-1.998516
C	-0.817952	2.884947	4.083954	O	-5.428437	-1.019974	3.100213
C	0.860638	3.007881	0.888075	O	-6.806364	-0.485114	1.417469
C	1.318621	3.524873	-0.346741	H	-6.209694	-1.526768	3.373102

Energy (m06-2X/6-31++G**) = -1444.8030372 Hartree

Frequencies(cm-1): -403.0700, 33.8011, 35.9222,...

Cartesian coordinates, energy, imaginary and low frequencies of the optimized geometry [PCM (ε = 46.7) M06-2X/6-31+G(d,p)] of:

TS-1-MeTAD

C	-1.861283	0.048375	2.763480	H	-3.006135	-1.576085	3.577775
C	-0.920304	0.353436	1.794092	H	-2.079181	-3.284738	2.064072
C	-0.382898	-0.597944	0.916519	H	-0.375749	-2.684402	0.345395
C	-0.789430	-1.927541	1.004571	H	2.101922	0.066345	-1.431670
C	-1.742500	-2.257119	1.973946	C	2.674459	-1.011364	0.368726
C	-2.269494	-1.287433	2.835689	H	1.254695	-1.458240	-1.211471
N	-0.348192	1.588995	1.451713	H	3.206902	3.453099	-0.156917
C	0.460941	1.488449	0.405536	H	2.857059	2.435692	1.254261
C	0.451048	0.105303	-0.059458	H	3.146375	1.694461	-0.335619
C	1.638545	-0.580843	-0.679851	H	1.565779	3.633643	-2.040585
C	1.191285	2.687521	-0.149292	H	1.347505	1.879315	-2.187726
C	0.995819	2.774939	-1.673127	H	-0.059724	2.913659	-1.917204
C	0.665352	3.981193	0.487306	H	1.179823	4.831495	0.032767
C	2.693947	2.546308	0.177764	H	-0.407906	4.104201	0.307587
N	-0.731565	0.133331	-1.622696	H	0.864044	4.022579	1.563956
N	-0.992659	-1.120731	-1.968617	H	-4.193803	-0.000635	0.534116
C	-2.293000	-1.427167	-1.609821	H	-4.748281	-1.075328	-0.780049
N	-2.879445	-0.266697	-1.073567	H	-0.599841	2.466923	1.891664
C	-1.946227	0.716725	-1.065984	C	4.000239	-1.479156	-0.241931
O	-2.079516	1.872782	-0.705536	H	2.875118	-0.179820	1.056992
C	-4.222841	-0.148844	-0.548695	H	2.254884	-1.823066	0.979508
O	-2.894202	-2.474545	-1.805960	C	4.992917	-1.833124	0.866233
H	-4.733458	0.693188	-1.019511	C	3.804742	-2.666673	-1.187092
H	-2.266115	0.803939	3.427891	H	4.416236	-0.641699	-0.822197

H	4.768190	-3.023221	-1.564698
H	3.321669	-3.497386	-0.657501
H	3.183981	-2.409042	-2.050064
H	5.961584	-2.125031	0.448840
H	5.153564	-0.986598	1.541717
H	4.615735	-2.673672	1.461143

Energy (m06-2X/6-31++G**) = -1148.010462 Hartree

Frequencies(cm-1): -408.9121, 33.9873, 46.1245,...

TS-2-MeTAD

C	-2.835514	-2.116394	1.249451
C	-1.999662	-1.550851	0.273583
C	-2.320444	-1.685090	-1.086236
C	-3.462216	-2.386677	-1.459811
C	-4.293574	-2.944126	-0.487194
C	-3.979856	-2.807159	0.866471
C	-0.789737	-0.837882	0.660634
N	-0.072489	-1.177994	1.729051
C	1.040438	-0.338117	1.881571
C	0.974041	0.620000	0.858143
C	-0.152540	0.281306	-0.013420
C	1.965050	1.593504	0.752882
C	3.014500	1.560365	1.675781
C	3.066791	0.587292	2.682200
C	2.070674	-0.385151	2.808186
C	-0.947212	1.357519	-0.706041
N	0.728908	-0.502469	-1.554841
N	1.501385	0.424425	-2.105795
C	2.824294	0.137845	-1.816357
N	2.861006	-1.073514	-1.102395
C	1.587913	-1.503038	-0.925817
O	3.816245	0.750800	-2.184226
C	4.042415	-1.722175	-0.574680
O	1.206727	-2.525617	-0.387766
H	4.909162	-1.167185	-0.933086
H	2.106286	-1.136670	3.589345
H	3.893850	0.589842	3.384085
H	3.802153	2.304260	1.616626
H	1.921217	2.355443	-0.019215
H	-1.625775	0.912551	-1.440876
C	-1.741860	2.209780	0.289878
H	-0.230414	1.972078	-1.259800
H	4.091841	-2.753598	-0.928388
H	4.021235	-1.709837	0.518201
H	-0.231452	-2.018731	2.273994
H	-1.651588	-1.273191	-1.834892
H	-3.702274	-2.500928	-2.511603
H	-5.187630	-3.483345	-0.783047
H	-4.630710	-3.232141	1.623041
H	-2.610246	-1.987506	2.304301
C	-2.589428	3.303395	-0.371322
H	-2.402906	1.555121	0.874490
H	-1.049317	2.678344	1.004123
C	-3.418913	4.030785	0.688276
C	-1.730001	4.300824	-1.151670
H	-3.279670	2.815680	-1.074862
H	-2.346266	5.113753	-1.548391
H	-0.971033	4.743517	-0.494302
H	-1.215447	3.833536	-1.995981
H	-4.065695	4.786482	0.231903
H	-4.050830	3.333328	1.247421
H	-2.760898	4.538885	1.403650

Energy (m06-2X/6-31++G**) = -1221.795122 Hartree

Frequencies(cm-1): -430.6288, 21.9928, 27.4484,...

TS-3-MeTAD

C	0.012508	0.001729	0.010565
N	0.001384	0.003591	1.468677
N	1.247190	0.002563	1.906209
C	2.106156	0.043921	0.815481
N	1.324191	0.016590	-0.349253
O	3.326882	0.010062	0.831608
C	1.822897	0.004385	-1.707244
O	-0.963338	-0.059749	-0.711001
C	-0.963860	1.586119	2.001729
C	-0.381839	2.500253	1.030252
N	-1.299496	2.761424	0.077139
C	-2.513590	2.139541	0.360960
C	-2.349665	1.419671	1.550069
C	-3.711433	2.174453	-0.344354
C	-4.768905	1.440681	0.186987
C	-4.627424	0.706776	1.376334
C	-3.419369	0.691320	2.069276
C	0.990387	2.965495	0.924685
C	1.527091	3.293657	-0.338725
C	2.857698	3.656705	-0.465794
C	3.681183	3.708681	0.665387
C	3.161227	3.402395	1.918555
C	1.826313	3.024684	2.051329
C	-0.665695	1.536235	3.475990
H	-1.134113	3.334638	-0.739558
H	-3.815634	2.738646	-1.264757
H	-5.722951	1.437259	-0.329899
H	-5.473491	0.145953	1.758841
H	-3.316448	0.119214	2.986021
H	-1.330543	0.806463	3.943078
H	0.361639	1.230076	3.671517
H	-0.851188	2.513210	3.931598
H	1.434236	2.807387	3.034800
H	3.793388	3.447838	2.798719
H	4.724391	3.989144	0.562478
H	3.261185	3.887120	-1.446011
H	0.915858	3.224132	-1.233938
H	1.330705	-0.790020	-2.270811
H	1.638384	0.965010	-2.194921
H	2.896027	-0.181338	-1.662325

Energy (m06-2X/6-31++G**) = -1064.610667 Hartree

Frequencies(cm-1): -407.7254, 35.5432, 46.8577,...

TS-4-MeTAD

C	0.067912	0.503691	0.461144
C	-0.110274	-0.012042	1.755112
C	1.025196	-0.250765	2.558255
C	2.295294	0.022502	2.077366
C	2.459733	0.534460	0.784719
C	1.348659	0.766166	-0.020154
C	-1.437389	-0.226156	2.301185
C	-2.666615	0.497548	2.014590
C	-3.647822	-0.100199	2.930731
C	-2.987121	-1.041622	3.731829
N	-1.659155	-1.074213	3.330604
C	-5.003250	0.137360	3.117944
C	-5.664510	-0.584690	4.114974
C	-4.981675	-1.527878	4.905544
C	-3.628059	-1.774424	4.726861
C	-3.130627	0.973007	0.663707
C	-7.113275	-0.385899	4.374191
O	-7.747892	-0.980858	5.221476
O	-7.673454	0.530550	3.570389
N	-2.146200	2.168520	2.751253
C	-1.475668	1.886107	4.016387
N	-0.229695	2.417784	3.894280
C	-0.108011	2.986468	2.618276
N	-1.321568	2.835339	1.962794
O	0.881911	3.585343	2.225861
C	0.814870	2.395321	4.895386
H	-0.954340	-1.666676	3.750933
H	-3.096469	-2.496825	5.335603
H	-5.538767	-2.065698	5.665024
H	-5.541037	0.865084	2.520015
H	-4.120327	1.422091	0.771776
H	-2.461318	1.726660	0.250399
H	-3.206900	0.127827	-0.025923
H	-0.784445	0.666938	-0.183669
H	1.472732	1.157503	-1.023982
H	3.455597	0.751334	0.412285
H	3.159203	-0.147537	2.711093
H	0.913280	-0.604868	3.579296
H	-8.613766	0.602854	3.800090
H	0.425818	1.879752	5.773590
H	1.686690	1.861764	4.508946
H	1.101584	3.414605	5.159953
O	-1.976523	1.334274	4.975905

Energy (m06-2X/6-31++G**) = -1253.137691 Hartree

Frequencies(cm-1): -428.6746, 36.5034, 46.8214,...

TS-5-MeTAD

C	0.020341	-0.307496	0.108354
C	-0.029370	-0.113201	1.490996
C	1.153948	0.156994	2.189035
C	2.366909	0.253591	1.511119
C	2.410499	0.068786	0.128943
C	1.236253	-0.215279	-0.567662
C	-1.289385	-0.334129	2.268625
C	-1.622086	-1.723569	2.611964
C	-2.237816	-1.724196	3.867598
N	-2.275764	-0.406901	4.321141
C	-1.693094	0.432751	3.438887
C	-2.695112	-2.885613	4.480316
C	-2.514552	-4.073951	3.776901
C	-1.893531	-4.095709	2.516811
C	-1.435904	-2.921183	1.924623
C	-1.665728	1.870890	3.618769
C	-2.443711	2.479742	4.630116
C	-2.489644	3.857364	4.749046
C	-1.760880	4.665488	3.864912
C	-0.990145	4.081436	2.868092
C	-0.942576	2.693460	2.735807
N	-2.640531	0.282673	1.059329
N	-2.414273	1.492629	0.588143
C	-3.421918	2.341191	1.035747
N	-4.314376	1.585737	1.805236
C	-3.878858	0.297080	1.837628
C	-5.504190	2.100877	2.448027
H	-2.730468	-0.123625	5.178399
H	-3.172769	-2.867441	5.453677
H	-2.857513	-5.005006	4.216140
H	-1.766092	-5.042493	2.003036
H	-0.949446	-2.934888	0.953800
H	-3.040463	1.883174	5.312864
H	-3.100170	4.309291	5.523423
H	-1.801462	5.745607	3.961430
H	-0.427220	4.700561	2.177981
H	-0.336499	2.257648	1.954283
H	1.122476	0.294869	3.267139
H	3.275653	0.470164	2.063414
H	3.354539	0.143646	-0.401227
H	1.263397	-0.362758	-1.642621
H	-0.895587	-0.515042	-0.434201
H	-6.129982	1.253931	2.729808
H	-5.234032	2.672774	3.340055
H	-6.039748	2.745728	1.750318
O	-3.584856	3.516542	0.748253
O	-4.412975	-0.669378	2.341326

Energy (m06-2X/6-31++G**) = -1256.281405 Hartree

Frequencies(cm-1): -390.8720, 30.6100, 38.0149,...

TS-6-MeTAD

C	-0.026054	0.003236	0.002254
N	0.003334	0.013538	1.464929
N	1.256335	0.025765	1.873632
C	2.086320	0.053615	0.756692
N	1.276557	0.033028	-0.386029
O	3.305549	0.008133	0.736906
C	1.761777	0.046266	-1.749737
O	-1.022613	-0.075296	-0.686098
C	-0.980402	1.539766	1.995120
C	-2.370975	1.340407	1.557451
C	-2.566290	2.069775	0.377630
N	-1.380052	2.727185	0.086753
C	-0.436392	2.481666	1.027804
C	-3.777403	2.078529	-0.310043
C	-4.805002	1.319490	0.231091
C	-4.629431	0.586422	1.420197
C	-3.408677	0.594962	2.099431
C	-0.744164	1.589482	3.473670
C	-0.657515	0.423000	4.237299
C	-0.564135	0.502925	5.626047
C	-0.558198	1.744169	6.261719
C	-0.654776	2.910795	5.502800
C	-0.753819	2.833280	4.115567
C	0.925677	2.957317	0.918159
C	1.404349	3.475316	-0.307939
C	2.732268	3.835263	-0.448122
C	3.618550	3.690194	0.629115
C	3.163043	3.183996	1.839371
C	1.827584	2.810160	1.988486
H	-1.240397	3.291248	-0.740926
H	-3.911456	2.645320	-1.224208
H	-5.772220	1.284659	-0.258578
C	-5.787909	-0.189484	1.931062
H	-3.278466	0.033077	3.018541
H	0.750399	3.564913	-1.169558
H	3.087725	4.218159	-1.398712
H	4.660061	3.971942	0.513158
H	3.845098	3.064065	2.674029
H	1.490960	2.418572	2.937917
H	-0.835189	3.742778	3.525288
H	-0.655520	3.881305	5.988320
H	-0.480821	1.802565	7.342656
H	-0.491840	-0.408522	6.210806
H	-0.648043	-0.541101	3.740310
H	2.522597	-0.725418	-1.874071
H	0.916850	-0.153534	-2.408792
H	2.190892	1.023817	-1.985065
O	-5.523019	-0.847195	3.069827
O	-6.874188	-0.238684	1.390452
H	-6.323489	-1.326556	3.337490

Energy (m06-2X/6-31++G**) = -1444.808225 Hartree

Frequencies(cm-1): -404.5819, 33.2862, 37.5771,...

REFERENCES

(1) Wang, Z. *Comprehensive Organic Name Reactions and Reagents*; John Wiley \& Sons, Inc.: **2010**.
(2) Robinson, G. M.; Robinson, R. *J. Chem. Soc., Trans.* **1924**, 125, 827.
(3) Radl, S. *Adv. Heterocycl. Chem.*; Elsevier: San Diego, **1997**; Vol. 67.
(4) Heravi, M. M.; Derikvand, F.; Ghassemzadeh, M.; Neumüller, B. *Tetrahedron Lett.* **2005**, 46, 6243.
(5) Billiet, S.; De Bruycker, K.; Driessen, F.; Goossens, H.; Van Speybroeck, V.; Winne, J. M.; Du Prez, F. E. *Nat. Chem.* **2014**, 6, 815.
(6) Cookson, R. C.; Gupte, S. S.; Stevens, I. D. R.; Watts, C. T. *Org. Synth.* **1988**, 50-9, 936.
(7) Ritzeler, O.; Stilz, H. U.; Neises, B.; Jaehne, G.; Haber-Mann, J. *WO 01/30774 A1* **1999**.
(8) Zhao, Y.; Truhlar, D. G. *Theor. Chem. Acc.* **2008**, 120, 215.
(9) Liu, L. P.; Malhotra, D.; Paton, R. S.; Houk, K. N.; Hammond, G. B. *Angew. Chem., Int. Ed.* **2010**, 49, 9132.
(10) Hratchian, H. P.; Schlegel, H. B. *J. Chem. Phys.* **2004**, 120, 9918.
(11) Hratchian, H. P.; Schlegel, H. B. *J. Chem. Theory Comput.* **2005**, 1, 61.
(12) Fukui, K. *Acc. Chem. Res.* **1981**, 14, 363.
(13) Cramer, C. J.; Truhlar, D. G. **1996**, p 1.
(14) Barone, V.; Cossi, M. *J. Phys. Chem. A* **1998**, 102, 1995.
(15) Cossi, M.; Rega, N.; Scalmani, G.; Barone, V. *J. Comput. Chem.* **2003**, 24, 669.
(16) Takano, Y.; Houk, K. N. *J. Chem. Theory Comput.* **2005**, 1, 70.
(17) Frisch, M. J.; Trucks, G. W.; Schlegel, H. B.; Scuseria, G. E.; Robb, M. A.; Cheeseman, J. R.; Scalmani, G.; Barone, V.; Mennucci, B.; Petersson, G. A.; Nakatsuji, H.; Caricato, M.; Li, X.; Hratchian, H. P.; Izmaylov, A. F.; Bloino, J.; Zheng, G.; Sonnenberg, J. L.; Hada, M.; Ehara, M.; Toyota, K.; Fukuda, R.; Hasegawa, J.; Ishida, M.; Nakajima, T.; Honda, Y.; Kitao, O.; Nakai, H.; Vreven, T.; Montgomery Jr., J. A.; Peralta, J. E.; Ogliaro, F.; Bearpark, M. J.; Heyd, J.; Brothers, E. N.; Kudin, K. N.; Staroverov, V. N.; Kobayashi, R.; Normand, J.; Raghavachari, K.; Rendell, A. P.; Burant, J. C.; Iyengar, S. S.; Tomasi, J.; Cossi, M.; Rega, N.; Millam, N. J.; Klene, M.; Knox, J. E.; Cross, J. B.; Bakken, V.; Adamo, C.; Jaramillo, J.; Gomperts, R.; Stratmann, R. E.; Yazyev, O.; Austin, A. J.; Cammi, R.; Pomelli, C.; Ochterski, J. W.; Martin, R. L.; Morokuma, K.; Zakrzewski, V. G.; Voth, G. A.; Salvador, P.; Dannenberg, J. J.; Dapprich, S.; Daniels, A. D.; Farkas, Ö.; Foresman, J. B.; Ortiz, J. V.; Cioslowski, J.; Fox, D. J.; Gaussian, Inc.: Wallingford, CT, USA, **2009**.
